# Self-adaptive Ni nanoparticles in perovskite LaNi_1-x_Al_x_O_3_/CaO for durable CO_2_ capture and in-situ conversion

**DOI:** 10.1038/s41467-026-75734-x

**Published:** 2026-07-21

**Authors:** Bin Shao, Zhonghao Jia, Sheng Dai, Jun Hu

**Affiliations:** 1 State Key Laboratory of Green Chemical Engineering and Industrial Catalysis, School of Chemistry and Molecular Engineering, East China University of Science and Technology, Shanghai, China; 2 Key Laboratory for Advanced Materials and Feringa Nobel Prize Scientist Joint Research Centre, School of Chemistry and Molecular Engineering, East China University of Science and Technology, Shanghai, China

**Keywords:** Carbon capture and storage, Heterogeneous catalysis, Sustainability

## Abstract

Integrated CO_2_ capture and conversion (iCCC) technology is promising for carbon neutrality, but the high-temperature deactivation of dual-functional materials (DFMs) limits its practicality. Herein, we develop adaptive metallic nano-catalysts via in-situ exsolution-dissolution in perovskite-based DFMs, enabling self-adjustment during cyclic CO_2_/CH_4_ redox switching. Al-doping induces the Jahn-Teller distortion in LaNiO_3_ perovskite, making the lattice contracted to enrich Ni^2+^ and oxygen vacancies; thereby tailoring the smooth exsolution-dissolution of Ni nano-catalyst and creating fast O^2-^ migration channels for facilitating CO_2_ adsorption. The optimized perovskite LaNi_0.8_Al_0.2_O_3_/CaO exhibits exceptional durability over 50 cycles, achieving a high CO_2_ capture capacity of 10.2 mmol g_DFM_^−1^ and both high conversions of 91.5% for CO_2_ and 93.5% for CH_4_. The mechanism study by the in-situ characterizations and surface energy calculations confirms that heteroatomic doping modulates the metal-support interactions, providing a solution for the long-sought deactivation problems of sintering and coking.

## Abstract (web summary)

Integrated CO₂ capture and conversion is promising for carbon neutrality, but the high-temperature deactivation of dual-functional materials (DFMs) limits its practicality. Here, the authors develop adaptive metallic nanocatalysts via in situ exsolution–dissolution in perovskite DFMs, enabling self-adjustment during cyclic CO₂/CH₄ redox switching and mitigating sintering and coking.

## Introduction

The development of integrated CO_2_ capture and conversion (iCCC) technology has proven to be one of the most effective approaches to alleviate the environmental and energy dilemmas, since it directly upcycles the captured CO_2_ into high-value chemicals and circumvents the energy-intensive and expensive processes of CO_2_ compression and transportation^1–3^. The successful iCCC technology lies in the development of stable dual-functional materials (DFMs), which contain both CO_2_ adsorptive sites and catalytic sites^4,5^. Conventionally, metal nanoparticles (NPs) (e.g., Ni, Pt and Ru) catalysts supported on various alkaline (Earth) metal oxides (e.g., CaO, MgO, Na_2_O and K_2_O) have been widely employed in design and fabrication of DFMs^6–8^. However, these DFMs often suffer from the deactivation through sintering^9,10^, especially for the high temperature iCCC, when taking place above the Tammann temperature of metals or oxides^11^.

Metal-support interactions (MSI) are critical determinants of sintering propensity^12–14^. Thus, rational tailoring MSI is a key to developing sinter-resistant DFMs for high-temperature applications^15,16^. Noteworthily, the motion-induced aggregations are also strongly affected by reaction parameters^17^. Intuitively, the intelligent strategy of in-situ exsolution-dissolution stands out as a promising approach^18,19^. Considering the high-temperature demands of iCCC, thermally robust perovskites with well-defined ABO_3_ architectures have emerged as a focal point among various intelligent candidates, as they can not only in situ generate nano-catalytic sites in reduction environments, but also reversely reintegrate back to parent perovskites in oxidation environments^20–22^. To tune the selective growth of uniform nanocatalysts through the exsolution-dissolution, some rational strategies have been developed, such as controlling nonstoichiometry to introduce defects, tailoring B-site compositions, and engineering the rough surface^23–25^. However, most of them are illustrated in artificially designed cycle redox environments, but have scarcely found real practical application scenarios.

Different from the conventional reduction or oxidation processes, the inherent redox cycling in iCCC switching between oxidative CO_2_ capture and reductive conversion may unlock the potential to achieve sinter resistance^26,27^. We selected a representative iCCC process featuring the dry reforming of methane as the conversion step (iCCC-DRM), as it can convert two greenhouse gases (CO_2_ and CH_4_) into useful industrial building blocks of syngas^10,28,29^. Ni/CaO has been recognized as the most effective DFM due to the excellent catalytic activity of Ni site for CH_4_ activation and theoretical CO_2_ capture capacity (17.8 mmol/g CaO)^30,31^. However, the most challenges lie in the dynamic volume expansion/contraction between CaO (16.9 cm^3^/g) and CaCO_3_ (36.9 cm^3^/g)^32,33^, inevitably aggravating sintering, meanwhile, compromising the MSI for the passive migration and coalescence of Ni species. Even more detrimentally, large Ni nanoparticles (>20 nm) are prone to coke deposition during high-temperature CH_4_ cracking^34,35^, which occludes active sites and consequently results in rapid deactivation. Spatial isolation strategies have been predominantly achieved by doping high Tamman temperature oxides or perovskite (e.g., MgO, Al_2_O_3_, CeO_2_, ZrO_2_, TiO_2_, CaTiO_3_, LaFeO_3_)^36–39^ to prevent the coalescence of CaO through the physical confinement effect^40,41^. Nevertheless, achieving simultaneous enhancement of the CO_2_ adsorption capacity and conversion efficiency remains challenging in iCCC-DRM. Although some approaches have been developed to simultaneously promote DFMs’ stability and reducibility^42^, the promising MSI regulation strategy remains far from well-studied. Specifically, a comprehensive understanding of the reversible exsolution-dissolution mechanism of catalytic sites in perovskites phase and the cooperation between active metal sites and adsorptive sites during iCCC represents a significant knowledge gap in rational design of DFMs.

**Fig. 1:**
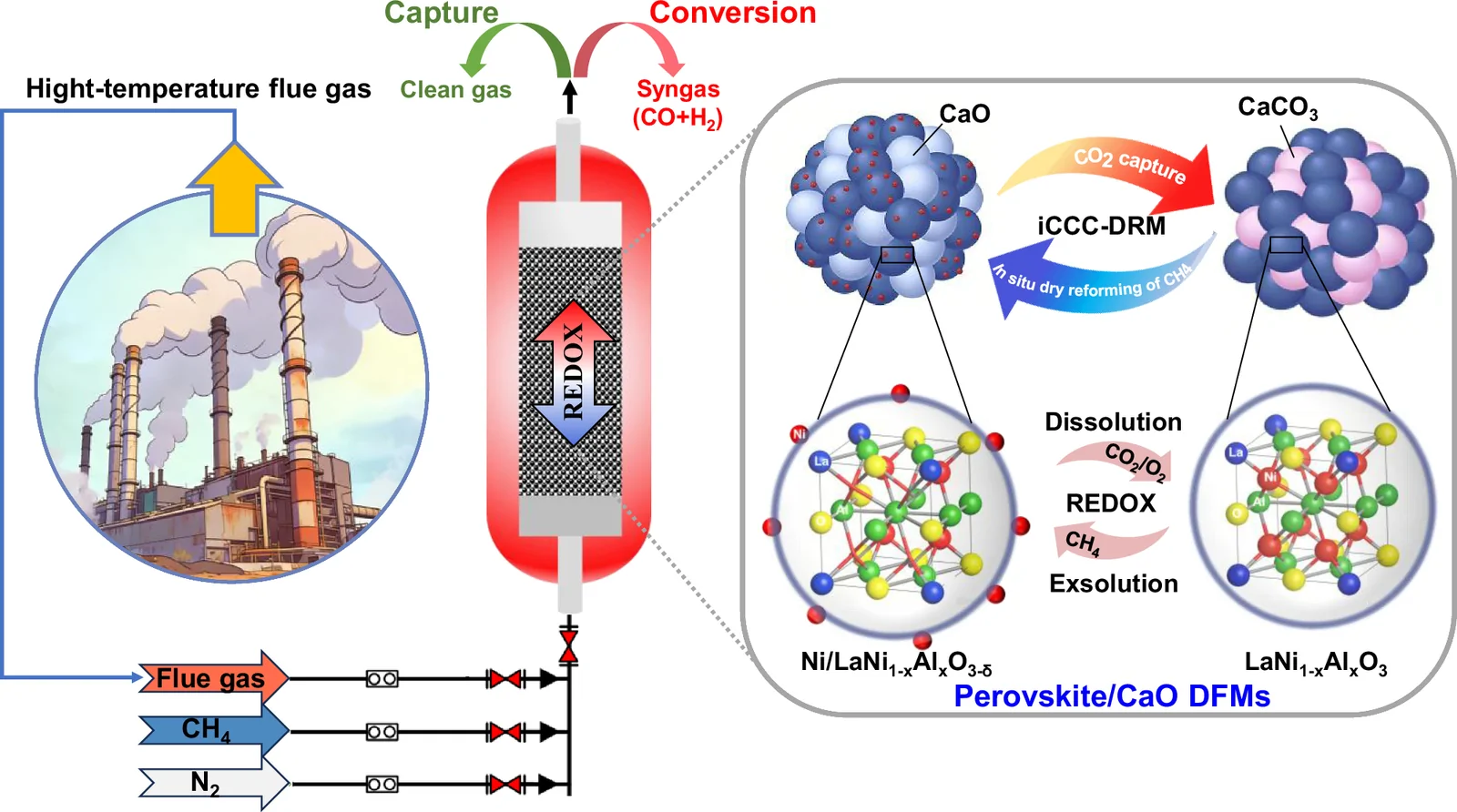
Schematic illustration of self-adaptive metallic nano-catalysts for high temperature iCCC-DRM process. In-situ reversible exsolution-dissolution process of perovskite LaNi_1-*x*_Al_*x*_O_3_/CaO DFMs during cyclic CO_2_/CH_4_ redox switching.

Herein, we proposed a self-adaptive metallic catalyst approach to develop long-life perovskite-based DFMs for durable iCCC-DRM redox cycles. The DFMs is consisted of highly dispersed LaNi_1-*x*_Al_*x*_O_3_ perovskite phase within CaO grains, where the Ni NPs are resolved into perovskite during oxidative CO_2_ capture step and exsolved during subsequent reductive DRM (Fig. 1). Through heteroatomic Al-doping in the B-site of LaNiO_3_, the preserved perovskite structure can enhance the MSI for high dispersion of Ni NPs, accommodate the reversible exsolution-dissolution under alternating redox atmosphere, produce abundant oxygen vacancies (Ov) via the lattices Jahn-Teller distortion. Accordingly, the redox pathway of in-situ exsolution-dissolution of Ni NP with proper surface energy (*E*_adh_) and chemical potential (*µ*), as well as the new pathway of O^2−^ ion migration channel for fast CO_2_ adsorption, can be well tailored to enhance the efficiency and stability of the cyclic iCCC-DRM process.

## Results

### Reversible exsolution-dissolution of Ni in perovskite DFMs for iCCC-DRM

**Fig. 2:**
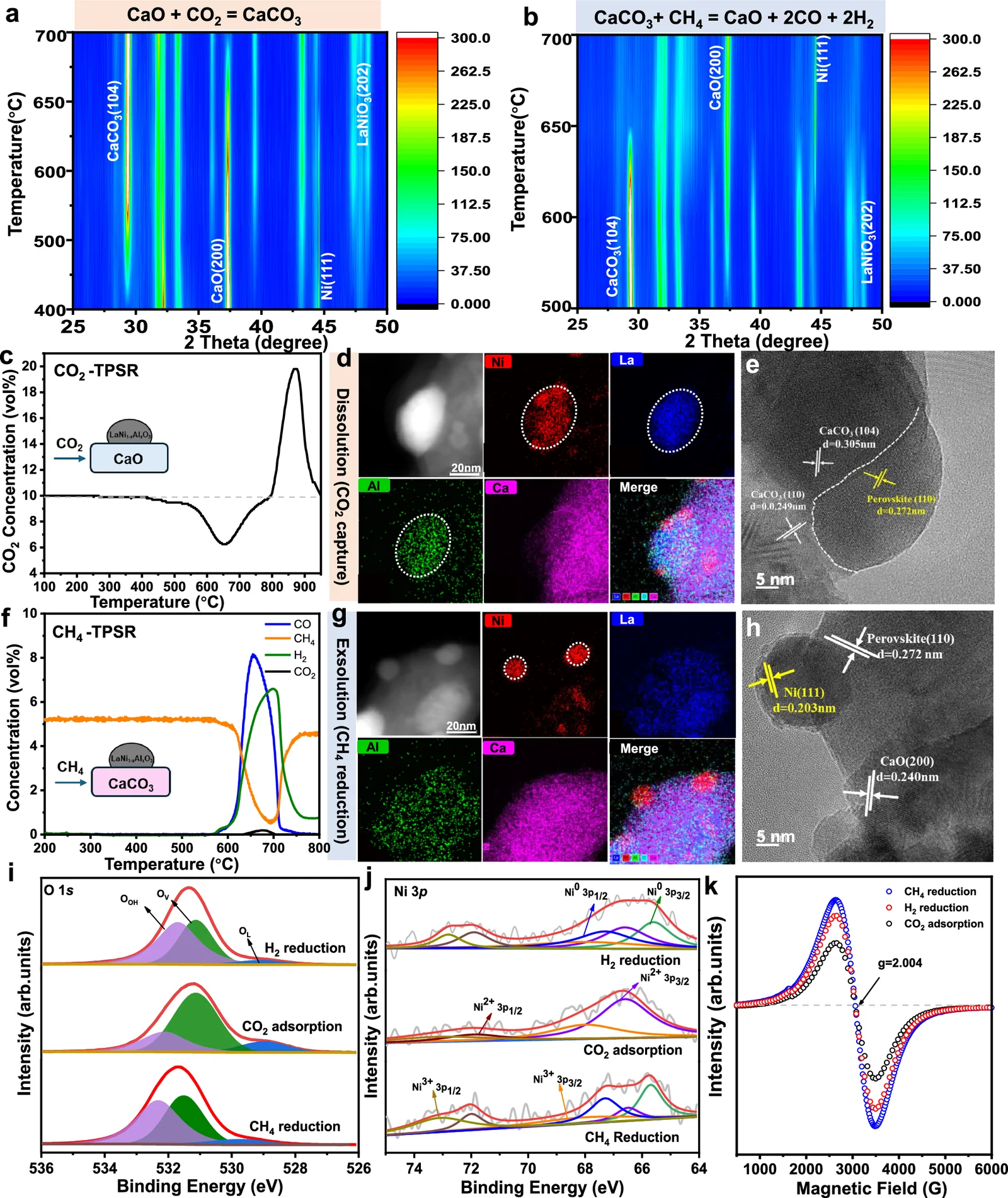
Reversible exsolution-dissolution performance on perovskite-type DFM (LaNi_0.8_Al_0.2_O_3_/CaO) during redox iCCC-DRM. In-situ XRD patterns during **a** CO_2_ capture after the H_2_ pretreatment and **b** subsequent DRM conversion. **c** CO_2_-TPSR profile of the LaNi_0.8_Al_0.2_O_3_/CaO. **d** STEM-HAADF and corresponding EDS elemental maps, **e** HRTEM images after CO_2_ capture. **f** CH_4_-TPSR profile of the LaNi_0.8_Al_0.2_O_3_/CaO after CO_2_ pre-adsorption. **g** STEM-HAADF and corresponding EDS elemental maps, **h** HRTEM images after subsequent DRM conversion. Quasi-in situ high-resolution XPS spectra of **i** O1*s* **j** Ni 3*p* and during each step. **k** EPR curves of LaNi_0.8_Al_0.2_O_3_/CaO DFM during iCCC-DRM process.

The iCCC-DRM technology includes two sequential steps: CO_2_ capture from flue gas under oxidative condition and subsequent methane reforming with adsorbed CO_2_ under reductive condition (Fig. 1). To make these tandem processes mutually inclusive in one integrated column, we fabricated DFMs composing of CaO adsorbent and LaNi_1-*x*_Al_*x*_O_3_ perovskite-type catalyst by a convenient one-pot sol-gel method (Supplementary Fig. 1). Specifically, The XRD pattern of the fresh LaNi_0.8_Al_0.2_O_3_/CaO DFM shows typical CaO (JCPD 48-1467) crystalline and multiple nickel species in the forms of LaNiO_3-δ_ (JCPD 34-1181) perovskite and a small amount of Ca(OH)_2_ phases (JCPD 44-1481), but no distinct characteristic peaks of any Al species (Supplementary Fig. 2), indicating the highly dispersed Al species. Owing to the selected calcination temperature of 800 °C, instead of the formation of CaAl_2_O_4_ (above 1100 °C)^43^, most Al can partially substitute B site of Ni in LaNiO_3_ perovskite lattice, which is highly anticipated to suppress the framework collapse of perovskite during the in-situ Ni exsolution. HAADF-STEM image shows that LaNi_0.8_Al_0.2_O_3_ perovskite particles are dispersed in CaO matrix, which will exert a spatial isolation effect to inhibit the sintering of CaO (Supplementary Fig. 3). While the EDS mapping images demonstrate the almost overlapped La, Ni, and Al elements in LaNi_0.8_Al_0.2_O_3_ perovskites. After the H_2_ pretreatment (Supplementary Fig. 4), HRTEM images illustrate the well-maintained dispersion state of perovskite particles in LaNi_0.8_Al_0.2_O_3_/CaO DFM (Supplementary Fig. 5). From the enlargement, we can see the exsolved Ni NPs (with lattice fringes of 0.203 nm) partially exsolve from the (110) surface (with the lattice of 0.272 nm) of LaNi_1-x_Al_x_O_3_ perovskite matrix. Then, the in-situ X-ray diffraction (XRD) was adopted to record the phase transformations during iCCC-DRM processes. At the step of CO_2_ capture from the simulated flue gas of 10 vol% CO_2_ and 2 vol% O_2_ balanced by N_2_, the diffraction intensity of the peaks of CaO (200) at 37.359° and Ni^0^ (111) at 44.507° exhibit a notable decrease, while the stepwise increase in intensity of peaks of CaCO_3_ (104) at 29.400° and LaNiO_3-δ_ (202) at 47.357°(Fig. 2a), revealing that the CaO phase transformed into CaCO_3_ through chemically adsorbed CO_2_ and the Ni NPs are resolved into LaNiO_3-δ_ perovskite under oxidative CO_2_ condition. Subsequently, at the step of in-situ conversion step under reductive CH_4_ atmosphere, the CaCO_3_ (104) peak starts to disappeared at 550 °C and CaO (200) peak reversely appears (Fig. 2b). Notably, accompanied by the appearance of the Ni^0^ (111) peak, the diffraction intensity of LaNiO_3-δ_ (202) peak decreases with the temperature, suggesting the Ni NPs are exsolved from the host Al-doped LaNiO_3_ perovskite.

More evidently, during the CO_2_ capture step, in line with the thermodynamic predictions of the high-temperature carbonation/decarbonization (Supplementary Fig. 6), the temperature programed surface reaction (CO_2_-TPSR) profile of LaNi_0.8_Al_0.2_O_3_/CaO exhibits a downward CO_2_ adsorption peak centered at 650 °C for the carbonation and an upward CO_2_ release peak at 850 °C for the decarbonization in an atmosphere containing 10 vol% CO_2_ (Fig. 2c). Significantly, an additional small downward peak appears at 500 °C, which can be attributed to the CO_2_ adsorption on oxygen vacancy of perovskite oxide induced by the pre-reduction^44,45^. This was verified by the quasi-in situ high-resolution O1*s* XPS spectra, that the intensity ratio of oxygen vacancies (O_V_) peak at 531.5 eV to that of lattice oxygen (O_L_) at 528.5–529.5 eV decreases significantly from 6.6 to 1.4 after the CO_2_ adsorption (Fig. 2i and Supplementary Table 1). Far beyond, evidenced by the Ni 3*p* XPS spectra, the Ni^0^ 3*p*3/2 peak around 65.6 eV diminishes with Ni^2+^ 3*p*3/2 and Ni^3+^ 3*p*3/2 peaks co-appearance at 66.7 eV and 68.3 eV^46,47^, respectively (Fig. 2j). Visually, the high-angle annular dark-field signal (HAADF) and energy dispersive spectrometer (EDS) elemental images provide clear evidence that Ni species are evenly distributed with La and Al in CaCO_3_ matrix (Fig. 2d and Supplementary Fig. 7), confirming the exsolved Ni NPs are re-incorporated into the host perovskite under the oxidative CO_2_ atmosphere after the capture step. However, some larger Ni NPs still existed in the perovskite due to the kinetic retardation during re-dissolution. Significantly, the LaNi_1-x_Al_x_O_3_ perovskite particles are in intimate contact with CaCO_3_ to form heterogeneous interfaces (Fig. 2e), successfully preventing sintering and agglomeration of Ni NPs and CaO during volumetric expansion.

Meanwhile during the subsequent DRM conversion step, CH_4_-TPSR profile of the LaNi_0.8_Al_0.2_O_3_/CaCO_3_ with pre-adsorbed CO_2_ shows that both CH_4_ consumption and H_2_ production started at 550 °C (Fig. 2f). Notably, the onset of CO generation is nearly overlapping with that of H_2_ and both peaks centered at 600–700 °C, much lower than the reported operating temperatures of 700–800 °C of the conventional DRM^48,49^. This well-matched activation kinetics between methane and adsorbed CO_2_ indicates that catalytic Ni NPs exsolve rapidly without any being inhibited by the volume expansion of CaCO_3_ grain^50^. Instead, the in-situ exsolved Ni NPs remain in close contact with CaCO_3_ matrix, facilitating H* species spilling over to the CO_3_^2−^ for more efficient DRM conversion, which can even inherently suppress carbon deposition by the synergistic activation pathways we illustrated before^51^. This was visually evidenced by the HAADF and HRTEM images, that the exsolved Ni NPs with the size of about 15 nm can be explicitly found on the surface of LaNi_0.8_Al_0.2_O_3_/CaO DFM (Fig. 2g and Supplementary Fig. 8), with the distinct lattice fringes of 0.203, 0.272, and 0.240 nm, corresponding to three phases of Ni NPs, LaNi_1-x_Al_x_O_3_ perovskite and CaO coexisted after the conversion (Fig. 2h). More evidently, the EDS images demonstrate that beside the exsolved Ni NPs, some Ni species are still remained in the parent LaNi_1-*x*_Al_*x*_O_3_ perovskite, which are all embodied in CaO matrix. In addition, the high-resolution Ni 3*p* XPS spectra show the surface Ni^2+^/Ni^3+^species are consumed, and a proportion of surface Ni^0^ species significantly increases to 45.85%, much higher than that of 34.50% in the H_2_ reduction pretreatment step (Fig. 2j and Supplementary Table 2). Moreover, the ratio of O_V_/O_L_ contrarily increases to 6.8 again during the reductive methane atmosphere, as lattice oxygen would react with methane molecules, promoting the in-situ exsolution of Ni NPs and the generation of O_V_s (Fig. 2i, k and Supplementary Table 1).

### Regulation activity and stability of perovskite-type DFMs through Al-doping

**Fig. 3:**
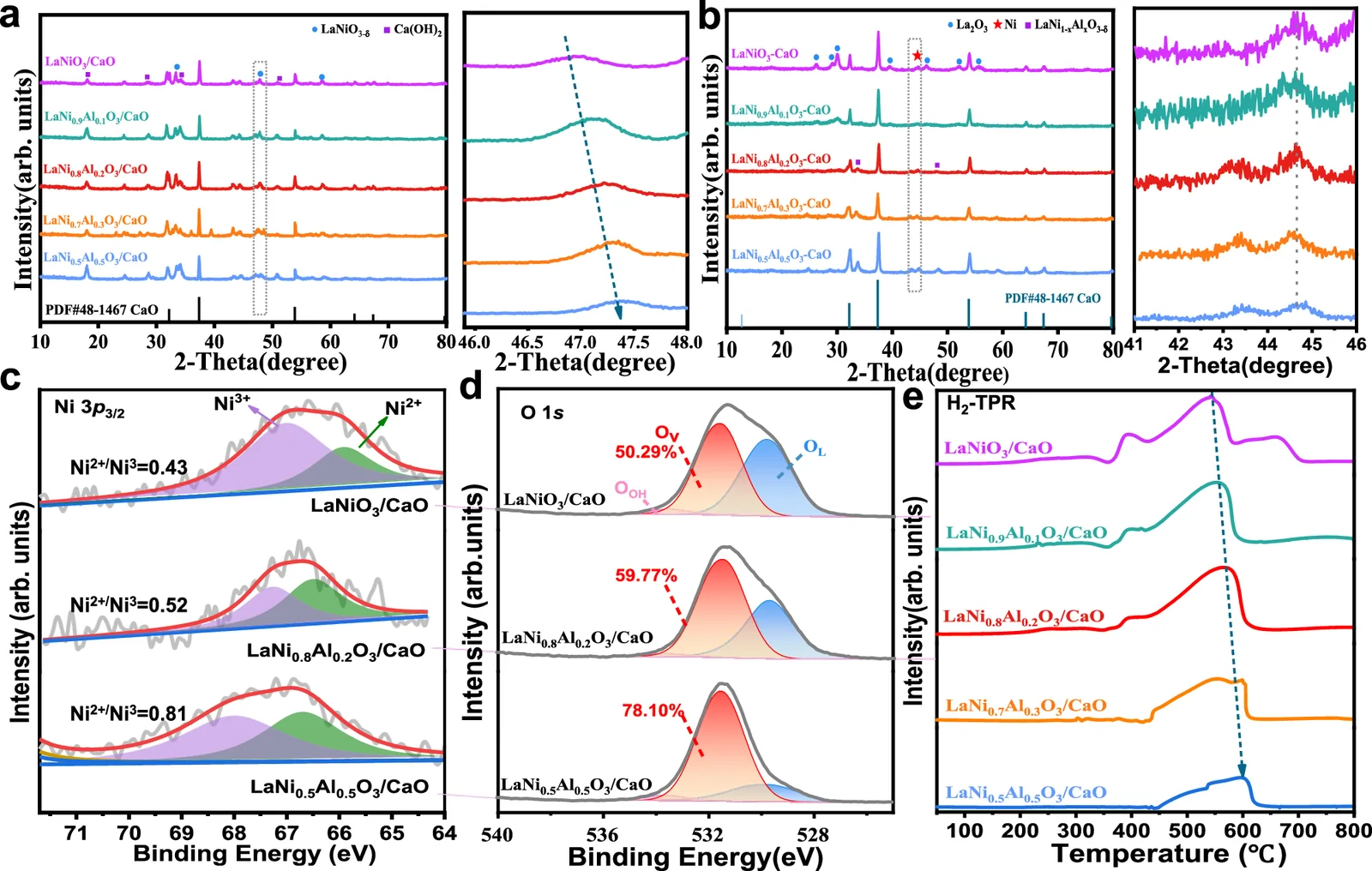
Synthesis and structural characterizations of Al-doped LaNiO3/CaO DFMs. XRD patterns and enlarged local XRD pattern of **a** as-prepared and **b** reduced LaNi_1-*x*_Al_*x*_O_3_/CaO (x = 0, 0.1, 0.2, 0.3, 0.5). XPS spectra corresponding to **c** Ni 3*p* and **d** O 1*s* of LaNiO_3_/CaO, LaNi_0.8_Al_0.2_O_3_/CaO, and LaNi_0.5_Al_0.5_O_3_/CaO. **e** H_2_-TPR profiles of the as-prepared LaNi_1-*x*_Al_*x*_O_3_/CaO.

To face the challenges associated with the stable activity of exsolved Ni NPs and the facile structural collapse of perovskite LaNiO_3_ during iCCC-DRM, we developed series of LaNi_1-*x*_Al_*x*_O_3_/CaO perovskite-type DFMs via modulating the molar ratio of Al^3+^ substituting for Ni^2+^/Ni^3+^ (*x* = 0.1, 0.2, 0.3, and 0.5), which was determined by Inductively Coupled Plasma Optical Emission Spectrometer (ICP-OES) and confirmed to be consistent with the stoichiometry ratios of designed perovskite compositions (Supplementary Table 3). Various amounts of Al-doping in LaNi_1-*x*_Al_*x*_O_3_/CaO DFMs lead no significant changes in their XRD patterns, that besides the typical peaks of CaO, only characteristic peaks of LaNiO_3-δ_ perovskites can be observed. Even when the *x* value of Al-doping is as high as 0.5, there is no characteristic peaks of any Al species appeared (Fig. 3a). Notably, the enlarged diffraction peaks of LaNiO_3-δ_ (202) show a shift to the larger 2-Theta, suggesting the contraction of LaNiO_3_ perovskite structure, thereby, Al^3+^ is successfully substituted in the lattice LaNiO_3_ perovskite owing to the smaller Shannon ion radii of Al^3+^ (0.054 nm) than Ni^2+/3+^ (0.069 nm/0.056 nm). Accordingly, the lattice parameters decrease with the amount of Al-doping (Supplementary Table 4), which breaks the typical orthorhombic symmetry via Jahn-Teller distortion, resulting in a transition to Trigonal structure (R$\bar{3}$ c) with lattice parameters reduced from (*a* = *b* = 5.451 Å, *c* = 6.564 Å) of LaNiO_3_ to (*a* = *b* = 5.430 Å, *c* = 6.541 Å) of LaNi_0.8_Al_0.2_O_3_, for example. Notably, without Al-doping, the Ni NPs also exsolved from the LaNiO_3_/CaO after the H_2_ pretreatment. However, the characteristic perovskite peaks disappear in the XRD pattern, leaving the La_2_O_3_ and CaO phases formed (Fig. 3b), which can hardly accommodate the re-dissolution of Ni. Differently, the perovskite phase is well preserved in LaNi_1-*x*_Al_*x*_O_3_/CaO without any phase separation, demonstrating the Al-doping in LaNiO_3_ lattice could prevent the destruction of perovskite structure during reduction step. Inevitably, the increase in the Al-doping level leads to the diffraction peaks of Ni^0^ peaks progressively decreased. Owing to the Jahn-Teller distortion in perovskite structure induced by the smaller Al^3+^ substitution, the contraction lattices result in enhanced binding energy (BE) of Ni-O with Al substitution rate increases, providing a shift towards higher binding energy in Ni 3*p* XPS spectra of LaNi_1-*x*_Al_*x*_O_3_/CaO (Fig. 3c). Considering the nature of the charge disproportionation between Ni^3+^ and Ni^2+^ in B-site of ABO_3_ perovskite, after Al-doping, the ratio of Ni^2+^/Ni^3+^ of 0.43 in pristine LaNiO_3_/CaO soars into 0.81 in LaNi_0.5_Al_0.5_O_3_/CaO, demonstrating Al^3+^ substituted most of the Ni^3+^ in B-site of LaNi_1-*x*_Al_*x*_O_3_ perovskite. Accordingly, Al substitution induces more lattice distortion to generate abundant oxygen vacancies, resulting in O_v_s significantly increases from 50.29% in LaNiO_3_/CaO to 78.10% in LaNi_0.5_Al_0.5_O_3_/CaO based on the O1*s* XPS spectra (Fig. 3d). This was further verified by the EPR spectra that the intensity of the O_v_ peak in LaNi_0.5_Al_0.5_O_3_/CaO is almost 9.2-times higher than that in LaNiO_3_/CaO and CaO (Supplementary Fig. 9).

The reduction capability of as-prepared LaNi_1-*x*_Al_*x*_O_3_/CaO DFMs was further illustrated by the H_2_-temperature programmed reduction (H_2_-TPR) (Fig. 3e), where two reduction bands occurred, one is for the reduction of free NiO to Ni at about 400 °C, another is for that of bonded Ni species in perovskite lattices at higher temperature of 550–600 °C. For the bonded Ni reduction, different from two-step consecutive reduction peaks in the pristine LaNiO_3_ perovskite of Ni^3+^ to Ni^2+^ and further to the exsolved metallic Ni^52^, Al-doping in perovskite results in almost one emerged reduction band due to the significantly enhanced Ni^2+^/Ni^3+^ ratio. Consistent with the strengthened Ni-O band caused the contraction of perovskite, the reduction bands of bonded Ni species in LaNi_1-*x*_Al_*x*_O_3_ shift towards even higher temperatures as the Al substitution ratio (*x*) rises, indicating the metal-support interaction (MSI) between exsolved Ni NPs and parent perovskite enhanced. Nevertheless, with increasing Al-doping (*x* ≥ 0.3), the intensity of the reduction bands exhibits a progressive attenuation, aligning with the gradual decrease in the total Ni content in the LaNi_1-*x*_Al_*x*_O_3_/CaO DFM. Thus, it is essential to modulate the Al-doping level in LaNi_1-*x*_Al_*x*_O_3_/CaO DFM to balance the CH_4_ activation capability and the structural stability of the reversible Ni dissolution-exsolution process over multicycle redox iCCC-DRM.

### Optimization of iCCC-DRM performance on LaNi_1-*x*_Al_*x*_O_3_/CaO DFM

**Fig. 4:**
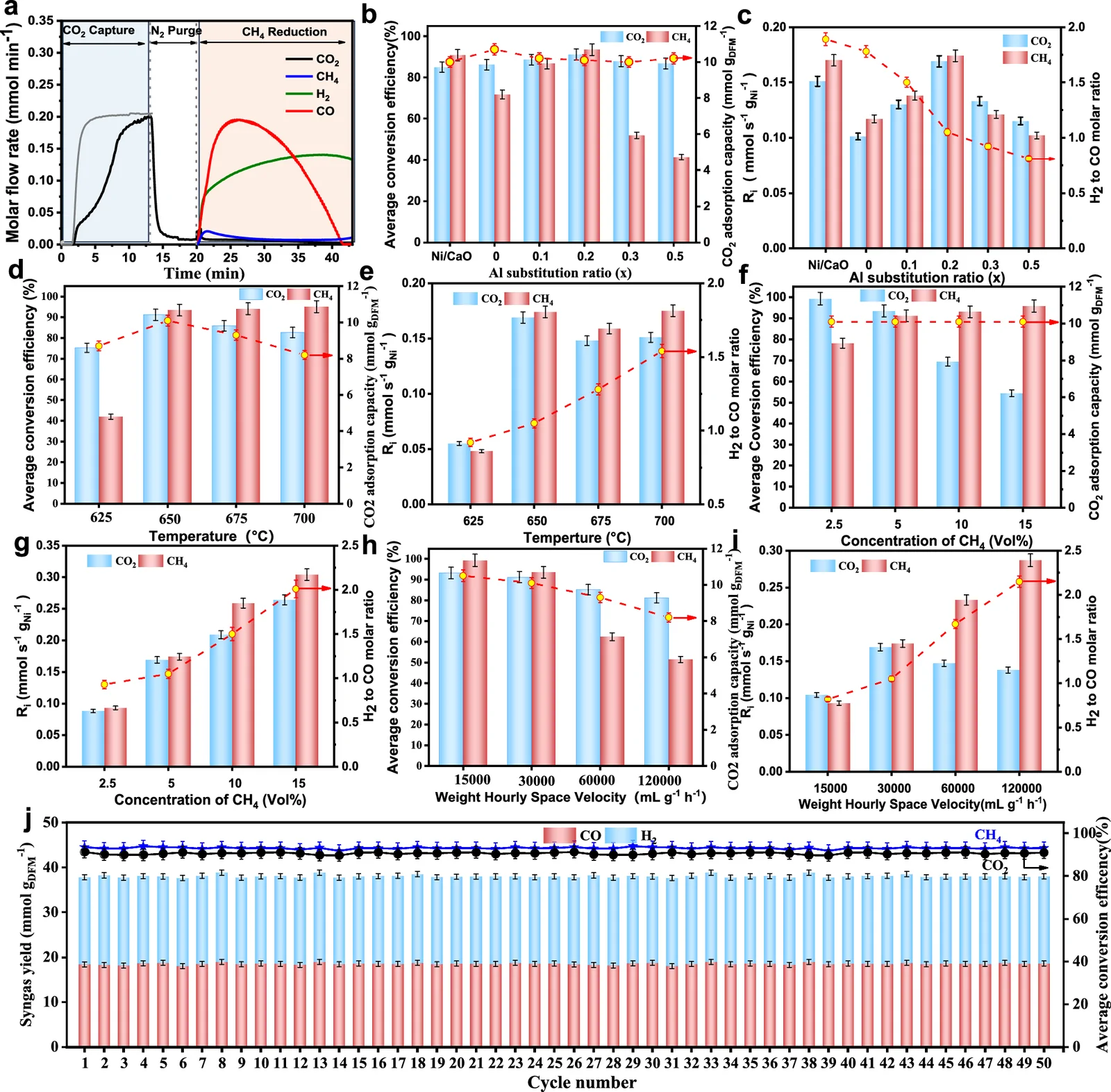
ICCC-DRM performance of LaNi_1-x_Al_x_O_3_/CaO DFMs. **a** Typical molar flow rate of each component in the effluent gas during one cycle of iCCC-DRM on LaNi_0.8_Al_0.2_O_3_/CaO DFM. The effects of Al-doping on **b** CO_2_ capture capacity and conversion efficiency, **c** syngas yield and the H/C ratio (*R*_H/C_) in the obtained syngas at 650 °C. Productivity and catalytic performance of iCCC-DRM under (**d**, **e**) different temperatures from 625 to 700 °C, (**f**, **g**) various concentrations of CH_4_ from 2.5 to 15 vol% and (**h**, **i**) various WHSVs from 15,000 to 120,000 mL g^−1^ h^−1^ at 650 °C. **j** Stability of LaNi_0.8_Al_0.2_O_3_/CaO DFM at 650 °C for 50 iCCC-DRM cycles. Error bars mean ± standard deviations calculated from three independent measurements.

The iCCC-DRM performance was systematically determined on the obtained LaNi_1-*x*_Al_*x*_O_3_/CaO (*x* = 0, 0.1, 0.2, 0.3, 0.5) DFMs in one fixed-bed reactor through consecutive tandem processes of CO_2_ capture, N_2_ purge, and conversion with methane at 650 °C (Fig. 4a and Supplementary Fig. 10). All the DFMs show similar high CO_2_ capacity of about 10.0 mmol g^−1^. While the in-situ DRM conversion efficiencies, particularly for methane conversion, show a volcano-type approach with the Al-doping level. Specifically, LaNi_0.8_Al_0.2_O_3_/CaO achieves the best iCCC-DRM performance with the highest CO_2_ and CH_4_ conversion efficiencies of (91.5%, 93.5%), much better than those of LaNiO_3_/CaO (86.2%, 71.7%) without Al-doping (Fig. 4b). Moreover, calculated in terms of per gram of Ni loading to improve the comparability in activity of among the LaNi_1-x_Al_x_O_3_/CaO DFMs with different Ni loadings, the LaNi_0.8_Al_0.2_O_3_/CaO exhibited the superior reaction kinetics with the highest CH_4_ and CO_2_ conversion rates of 0.169 mmolCO_2_ s^-1^ g_Ni_^−1^and 0.174 mmolCH_4_ s^−1^ gNi^−1^, respectively (Fig. 4c). In addition, the molar ratio of H_2_-to-CO (*R*_H/C_) in the outlet syngas produced is about 1.05, illustrating well-matched kinetics between CH_4_ dissociation and the reduction of adsorbed CO_2_ owing to the optimized exsolution of Ni NPs through the regulation of Al-doping in the perovskite matrix. In comparison, the much higher *R*_H/C_ in LaNiO_3_/CaO (*R*_H/C_ = 1.78) provide evidence of the deep dissociation of CH_4_, which eventually causes the carbon deposits and the catalytic deactivation (Supplementary Fig. 11). In contrast, too much higher *x* of Al-doping results in a significant decrease in exsolution of Ni NPs for CH_4_ activation, and hence a much lower *R*_H/C_.

To further optimize the iCCC-DRM performance, the reaction operation conditions were optimized on LaNi_0.8_Al_0.2_O_3_/CaO. As the temperature plays a crucial role in matching CO_2_ capture and in-situ catalytic conversion, we searched it in the temperature range of 625 °C to 700 °C. The optimal CO_2_ capture temperature is determined at 650 °C, with the capacity of 10.5 mmol g^−1^ (Figs. 4d, e). Meanwhile, the in-situ catalytic CH_4_ conversion efficiency continuously increases from 71.98% at 625 °C to 95.13% at 700 °C, owing to its thermodynamic favorable of high temperature. However, the CO_2_ conversion efficiency sharply decreases from 91.5% at 650 °C to 82.6% at 700 °C, since the dry reforming rate could not catch up with the fast CaCO_3_ decomposition at higher temperature. Overall, the temperature was optimally set at 650 °C.

In addition, the CH_4_ concentration and gas-weight hourly space velocity (WHSV) of the inlet gas at the conversion stage were also optimized. The CO_2_ conversion efficiency increased almost linearly from 78.2% in 2.5 vol% CH_4_ to 95.8% in 15 vol% CH_4_, but conversely bounded to even greater decline of the CH_4_ conversion efficiency from 99.3% to 54.4% (Fig. 4f, g). Excessive CH_4_ will certainly enhance the fast CO_2_ conversion, leaving insufficient time for CH_4_ conversion. Moreover, both the desorption rate of CaCO_3_ and the tandem DRM rate between desorbed CO_2_ and CH_4_ are strongly related to the WHSV of CH_4_. The CO_2_ conversion was kept similarly at about 90.0%, it takes 30 min, 21 min,15 min, and 11 mins to conduct the DRM when the WHSV was 15,000 mL g^−1^ h^−1^, 30,000 mL g^−1^ h^−^^1^, 60,000 mL g^−1^ h^−1^ and 90,000 mL g^−1^ h^−1^, respectively (Supplementary Fig. 12). The higher the WHSV, the faster the desorption and the DRM reaction rate, but the significantly decreased CH_4_ conversion efficiency when WHSV is above 30,000 mL g^−1^ h−^1^ (Fig. 4h, i).

Accordingly, the stability of iCCC-DRM was investigated on LaNi_0.8_Al_0.2_O_3_/CaO DFMs at 650 °C in a fixed-bed column under 10 vol% CO_2_ and 2 vol% O_2_ simulated flue gas followed by 5 vol% CH_4_ at GHSV of 30,000 mL g^-1^ h^-1^. After 50 cycles, LaNi_0.8_Al_0.2_O_3_/CaO shows a durable CO_2_ capture capacity of 10.2 mmol g^−1^, maintaining more than 98% stability due to the spatial confinement role of LaNi_0.8_Al_0.2_O_3_ perovskite in preventing CaO grains. Such stability of LaNi_0.8_Al_0.2_/CaO can be attributed to the perovskite’s high Tamman temperature (~800 °C) and its spatial confinement effect for separating CaO domains, which collectively prevent sintering and agglomeration during the redox iCCC-DRM process. More importantly, perfectly stable CO_2_ and CH_4_ conversion efficiencies of LaNi_0.8_Al_0.2_O_3_/CaO are observed as high as 91.5% and 93.5% without any decline at 650 °C (Supplementary Table 5), demonstrating the reversible feasibility of dissolution-exsolution of Ni NPs during the dynamically switching redox iCCC process (Fig. 4j).

To highlight the interfacial synergistic effect between CaO adsorption sites and perovskite catalytic sites of the DFM, the control samples with the fixed Ni content of pristine Ni/CaO, LaNiO_3_/CaO without Al-doping, as well as physical mixing DFM of LaNi_0.8_Al_0.2_O_3_/CaO-PM were also prepared and demonstrated (Supplementary Figs. 13–15). Significantly, after 25 cycles, the CO_2_ capture capacity of Ni/CaO DFM rapidly decreases from initial 10.7 mmol g_DFM_^−1^ to 4.8 mmol g_DFM_^−1^, with 55% decline, and the CH_4_ conversion efficiency decreases from 65.9% to 52.1% (Supplementary Figs. 16−18). During each cycle of conventional Ni/CaO DFM, the reversible phase transformation between CaO and CaCO_3_ weakens the metal-support interaction of Ni-CaO, thereby inducing severe sintering of Ni NPs and filamentous carbon depositions (Supplementary Fig. 19). Similarly, the LaNiO_3_/CaO without proper Al-doping also exhibited unsatisfactory cyclic stability, and the CO_2_ capture capacity of LaNiO_3_/CaO DFM decreases to 6.7 mmol g^−1^ with 33% decline and the CH_4_ conversion efficiency decreases from 71.7% to 43.0% after 25 cycles (Supplementary Figs. 16−18), which can be caused by the Ni NPs sintering due to the collapse of the LaNiO_3_ structure, and consequently a cascade of CaO sintering (Supplementary Fig. 20). For comparison, physical mixing LaNi_0.8_Al_0.2_O_3_/CaO-PM shows a significantly decay of 77.2% in adsorption capacity with a low CH_4_ conversion efficiency of 28.9 % after 25 cycles due to low Ni dispersion, which may induce carbon deposition for catalytic deactivation (Supplementary Figs. 16−18). This further demonstrates the crucial role of the interfacial interaction between intimate assembly of LaNi_0.8_Al_0.2_O_3_ and CaO for the enhanced iCCC-DRM performance.

## Discussion

### O^2−^ migration channels of perovskite-type DFMs for enhanced iCCC-DRM

**Fig. 5:**
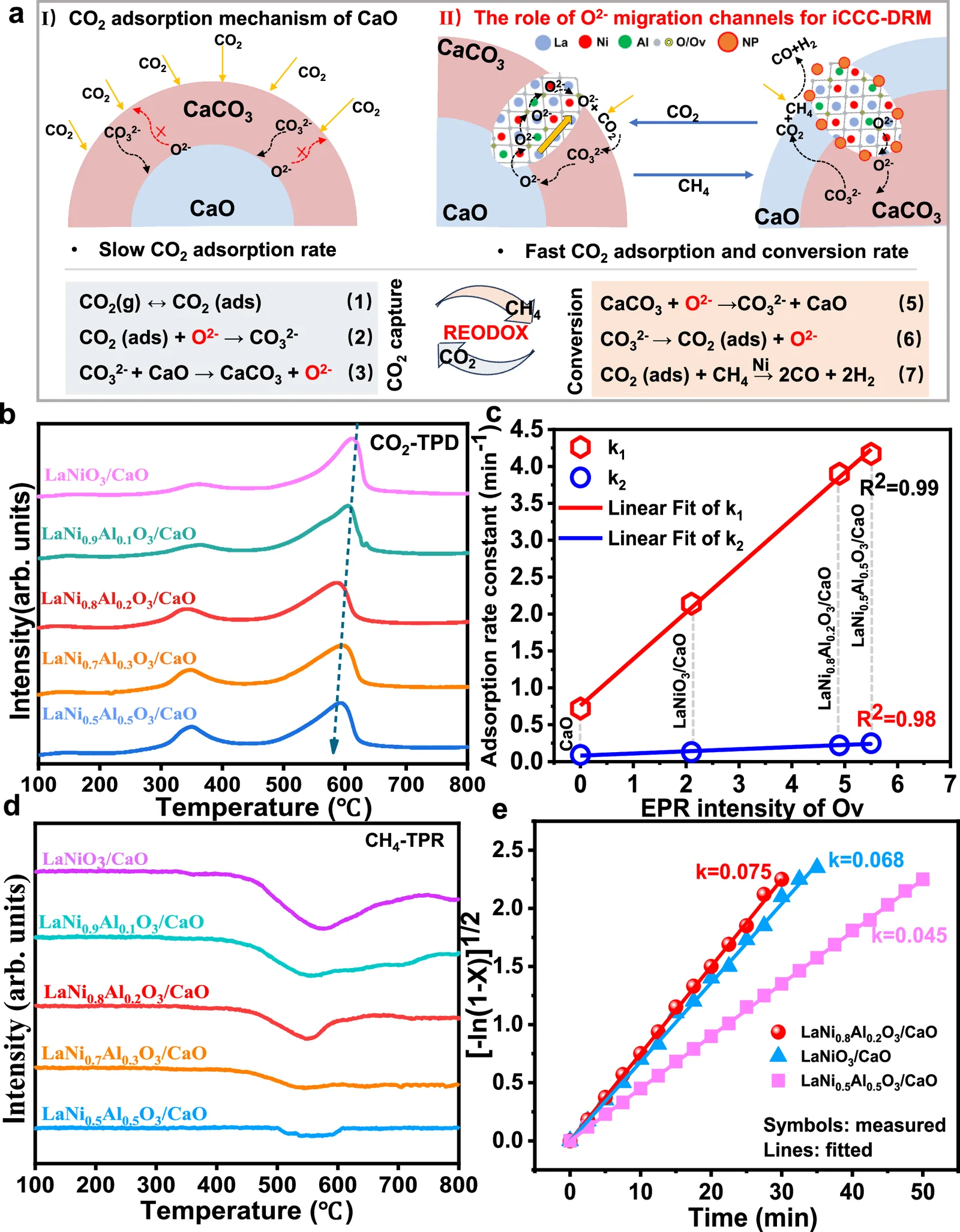
The O^2−^ migration channels generated in LaNi1-xAlxO3/CaO DFMs for enhancing the reactivity of iCCC-DRM. **a** Schematic diagrams of the role of O^2−^ migration channels in perovskite-type DFMs for iCCC-DRM. **b** CO_2_-TPD profiles of LaNi_1-x_Al_x_O_3_/CaO DFMs. **c** The regression fitting between *k*_1_(*k*_2_) and the intensity of O_v_ EPR signal of CaO and LaNi_1-*x*_Al_*x*_O_3_/CaO DFMs. **d** CH_4_-TPR profiles of LaNi_1-*x*_Al_*x*_O_3_/CaO. **e** Kinetic analysis of the in-situ DRM reaction on LaNi_1-x_Al_x_O_3_/CaO DFMs after CO_2_ adsorption (test conditions: 650 °C, 5 vol% CH_4_ /N_2_).

The conventional carbonation consists of three steps: ➀ surface adsorption of CO_2_, ➁ reaction with O^2−^ to generate CO_3_^2−^, and ➂ ion exchange with CaO to form CaCO_3_. Naturally, O^2−^ species will migrate outward to accommodate CO_2_ molecules. However, such O^2−^ migration proceeds sluggishly owing to lack of driving force, resulting in the serious retarded CO_2_ diffusion caused by the in situ formed dense CaCO_3_ layer over CaO (Fig. 5a, Ⅰ). It has been reported that oxygen vacancies (O_v_) can facilitate the CO_2_ adsorption via the O_v_ hopping mechanism^53–55^, which accelerates the O^2-^ migration through the CaCO_3_ product layer. Accordingly, introducing O_v_ in CaO-based adsorbent can serve as a promising strategy to address the challenge of sluggish CO_2_ diffusion through the CaCO_3_ layer.

Notably, with increasing the amount of Al substitution, the octahedron factor (*µ*) of LaNi_1-x_Al_x_O_3_ perovskite in DFMs decreases from 0.400 to 0.391 (Supplementary Table 4), revealing more lattice distortion and hence more O_v_s. Thanks to the intimately contacted between LaNi_1-x_Al_x_O_3_ perovskite and CaO (Supplementary Fig. 21), the abundant O_v_s generated in LaNi_1-*x*_Al_*x*_O_3_ can act as the driving force to induce reactive O^2−^ migration. Specifically, O^2−^ migrates to adjacent Oᵥ, leaving a new Oᵥ at the original lattice site and subsequently reoccupied by surrounding O^2−^. In this way, Oᵥs are dynamically occupied and regenerated, providing O^2−^ transport channels for CO_2_ to diffuse across the CaCO_3_ layer and react with inner CaO. This results in a revolution of the conventional layered-progression CO_2_ adsorption into a facilitating O^2−^ migration channels via the oxygen vacancy for efficient CO_2_ capture (Fig. 5a, Ⅱ). The CO_2_-TPD profile of LaNi_1-*x*_Al_*x*_O_3_/CaO DFMs provides intuitive evidence of an additional enhanced desorption peak at the medium-temperature range of 300-400°C, which can be attributed to the contribution of O_v_s on CO_2_ capture (Fig. 5b). Moreover, the shifts toward lower onset temperature of the CO_2_ desorption with increasing Al-doping further demonstrate the promotion effect of O_v_. This enhanced CO_2_ adsorption kinetic was illustrated by the double exponential model (Supplementary Eq. 1, see in the supplementary information), that at 650 °C, the initial fast chemical adsorption (carbonation) rate constant (*k*_1_) and subsequent CO_2_ diffusion-controlled rate constant (*k*_2_) of LaNi_0.5_Al_0.5_O_3_/CaO (4.17 min^−1^, 0.25 min^−1^) are 3–6 times larger than those of CaO (0.72 min^−1^, 0.09 min^−1^), respectively (Supplementary Fig. 22 and Supplementary Table 6). Importantly, we have identified a remarkable linear relationship between *k*_1_, or *k*_2_ and the intensity of O_v_ EPR signal (Fig. 5c), verifying the concentration of Oᵥ directly governs the O^2−^ migration barrier and accelerates CO_2_ diffusion. Therefore, LaNi_1-x_Al_x_O_3_ perovskite phase in DFMs boosts the CO_2_ adsorption kinetics through providing abundant O^2−^ migration channels in CaO matrix. What’s more, the O^2−^ ions diffusing and jumping in the lattice of perovskite is also conducive to the oxidation of Ni^0^ NPs and resolution back into the perovskite lattice structure (Supplementary Fig. 23).

Besides, the O^2−^ migration channel also accelerate the reaction rate of in situ DRM reaction after CO_2_ adsorption. The dynamic change of conversion efficiency of CaCO_3_, i.e., the adsorbed CO_2_ (*X*), was calculated by integrating the sum of the amounts of CO and CO_2_ in the effluent curve (Supplementary Fig. 24), which also exhibited a fast to slow two-stage characteristic due to the continuous consumption of adsorbed CO_2_ (in the form of CaCO_3_). Fitted well by the random nucleation and growth model of the Avrami-Erofeev (A2) equation^56^ (Supplementary Eq. 4), the slop of the linear plotting provides the reaction constant *k*, that LaNi_0.8_Al_0.2_O_3_/CaO (0.075) shows a faster DRM conversion than LaNiO_3_/CaO (0.068), although the total amount of reducible Ni sites in DFM is less (Fig. 5d, e). However, when *x* further increases, the DRM conversion rate shows a significant decrease on LaNi_0.5_Al_0.5_O_3_/CaO (0.045). This was further illustrated by their profiles of CH_4_-TPR that the CH_4_ consumption decreases with the *x* of Al-substitution owing to the decline in the total amount of reducible Ni sites (Fig. 5d). Therefore, the reaction rate of in-situ DRM reaction requires a rational Al substitution ratio in LaNi_1-*x*_Al_*x*_O_3_/CaO to match the CaCO_3_ desorption rate and DRM reaction rate.

### Mechanism of sinter-resistant Al-doped LaNiO_3_/CaO via tunning MSI

**Fig. 6:**
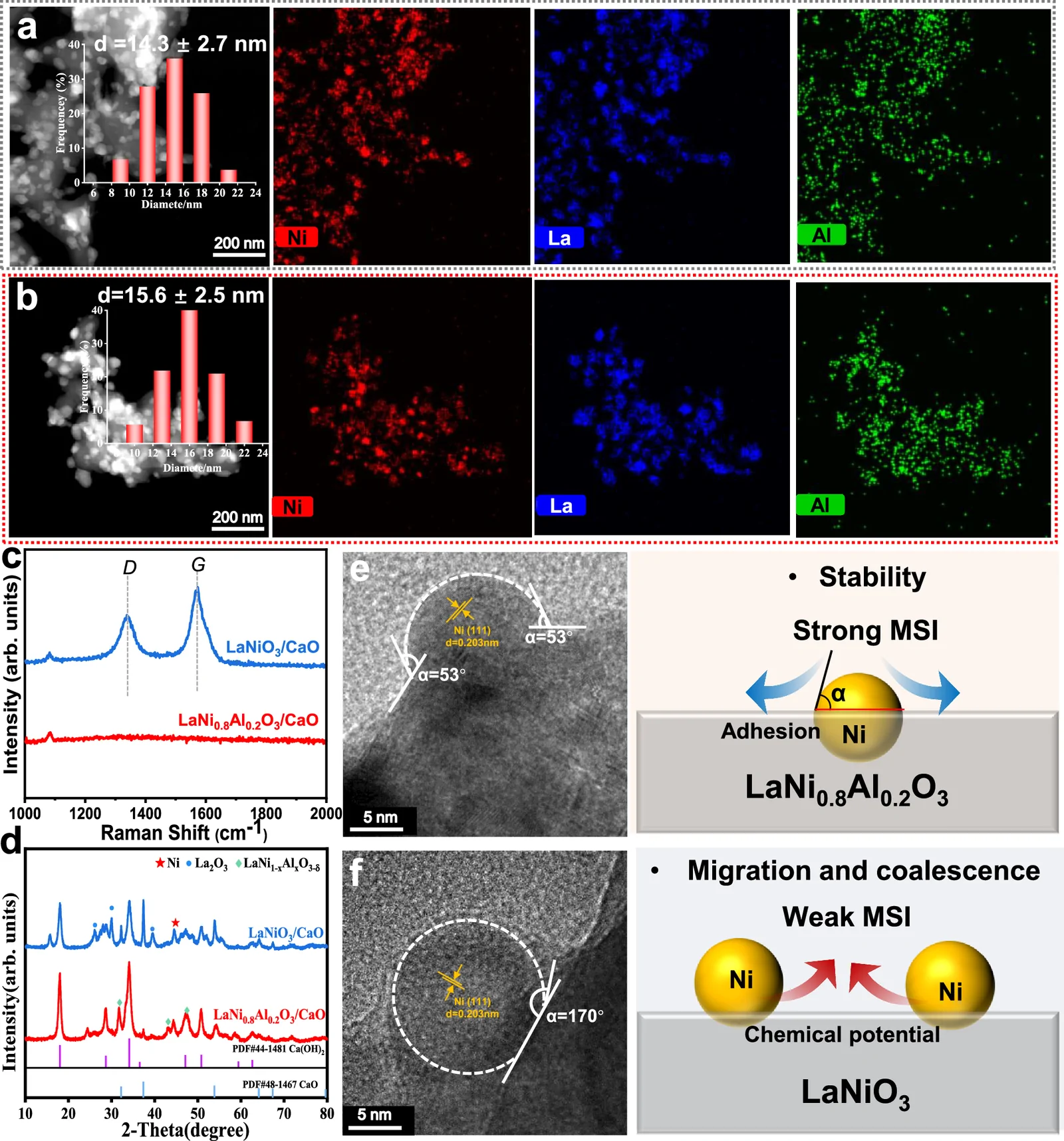
MSI governing reversible exsolution-dissolution of Ni NPs in Al-doped LaNiO3/CaO DFMs for the enhanced durability. HAADF-STEM images and corresponding size distribution of LaNi_0.8_Al_0.2_O_3_/CaO after **a** H_2_-pretreatment and **b** 50 iCCC-DRM cycles. Comparisons of **c** Raman spectra and **d** XRD patterns of spent LaNiO_3_/CaO and LaNi_0.8_Al_0.2_O_3_-CaO DFMs after the iCCC-DRM stability test. Illustration of the MSI between the exsolved Ni NPs and LaNi_1-*x*_Al_*x*_O_3_ perovskite (yellow spheres and gray rectangles represent Ni particles and perovskite phase, respectively), and the measured contact angles on their HR-TEM images of **e** LaNi_0.8_Al_0.2_O_3_/CaO and **f** LaNiO_3_/CaO after first iCCC-DRM cycle.

To further understand the mechanism of effective sinter-resistant governing the in-situ exsolution-dissolution of Ni NPs during the redox iCCC-DRM, the characteristic structure properties of spent LaNi_1-*x*_Al_*x*_O_3_/CaO DFMs were further investigated. Specifically, the HAADF-STEM images of the spent LaNi_0.8_Al_0.2_O_3_/CaO DFM after 50 cyclic iCCC-DRM indicate that the mean size of Ni NPs is properly maintained as 15.6 nm, with a neglectable particles sintering in compared with the Ni NPs of 14.3 nm after the first cycle (Fig. 6a, b). Moreover, the tolerance factor (*t*) of LaNi_1-x_Al_x_O_3_ is increased from 0.976 to 0.992 with the increase of Al doping (Supplementary Table 4), confirming the Al-doping can provide the geometric compatibility and structural stability of the LaNi_1-*x*_Al_*x*_O_3_ perovskite. Conversely, although LaNiO_3_/CaO shows a superior initial CH_4_ activation ability due to its readily exsolved Ni NPs, the catalytic activity decays very fast. The spent LaNiO_3_/CaO after 25 cyclic iCCC-DRM indicate the significantly increased size of Ni NPs from 14.4 nm to 36.07 nm (Supplementary Fig. 25). Consequently, confirmed by the presence of *G* band (~1580 cm^−1^) and *D* band (~1330 cm^−1^) in the Raman spectra of spent LaNiO_3_/CaO (Fig. 6c), the significant coking was formed on the enlarged Ni sintering particles, resulting in the low durability of iCCC-DRM. In contrast, there is no obvious carbon deposition on the spent LaNi_0.8_Al_0.2_O_3_/CaO DFM. This severe sintering of Ni NPs of LaNiO_3_/CaO can be caused by the structure collapse of LaNiO_3_ perovskite to La_2_O_3_ during Ni-exsolution (Fig. 6d).

We quantitively calculated the interfacial adhesion energy (*E*_adh_) of the exsolved Ni NPs with the LaNi_1-*x*_Al_*x*_O_3_/CaO DFM supports and their chemical potential (*µ*) to further elucidate the sintering-resistance effect (Supplementary Eqs. 7 and 10). As displayed in high-resolution TEM images, the exsolved Ni NP on LaNi_0.8_Al_0.2_O_3_/CaO and LaNiO_3_/CaO show similar mean size of 14.3 -14.4 nm, but absolutely different contact angle of 53° and 170°, respectively (Fig. 6e, f). Consistent with the smaller the contact angle, the higher the *E*_adh_^14^, the exsolved Ni NPs from LaNi_0.8_Al_0.2_O_3_/CaO exhibit the highest *E*_adh_ of 2.26 J m^−2^. This proper MSI enables the easy resolution of Ni atoms back into the well preserved LaNi_1-x_Al_x_O_3_ perovskite lattice under oxidative CO_2_ capture step, meanwhile, successfully inhibits the coalescence of Ni NPs through the Ostwald ripening pathway. More significantly, the Ni NPs sites produced by the in situ exsolution are partially embedded in perovskites, which display more resilient to coalescence than the deposited ones^57,58^, thereby exhibits significant stability potential in iCCC processes. Furthermore, the calculated chemical potential (*µ*) values of the exsolved Ni NPs from LaNiO_3_/CaO (227.7 kJ mol^−1^) are much higher than that from LaNi_0.8_Al_0.2_O_3_/CaO (171. kJ mol^−1^) (Supplementary Fig. 26). As expected, the Ni NPs with higher surface potential tends to aggregate on the surface of LaNiO_3_/CaO through the particles migration coalescence (PMC) pathway, resulting in the sintering, and, consequently the coking and deactivation.

Accordingly, the valence changes of Ni^0^, Ni^2+^, and Ni^3+^ at the B-site of LaNi_1-x_Al_x_O_3_ induced by the alternating reductive-oxidative (redox) atmosphere are the core driving force for Ni NPs exsolution and resolution (Supplementary Table 2). Without Al-doping to support the perovskite skeleton, the exsolution of Ni^0^ induced by CH_4_ reduction will trigger the structural collapse. Even though Ni species can be re-oxidized during CO_2_ capture stage, it can hardly reassemble with La_2_O_3_ species to reconstruct the perovskite crystal structure under iCCC-DRM reaction conditions (Supplementary Fig. 27a). Thus, the core purpose of Al doping in LaNiO_3_/CaO is to preserve the structural stability of the perovskite lattice for the accommodation of Ni dissolution and exsolution. The well matched CH_4_ and adsorbed CO_2_ activation owing to the proper MSI between the exsolved Ni NPs and LaNi_1-x_Al_x_O_3_ perovskite sustains the durable perovskite structure for dynamic Ni NPs exsolution-dissolution during the reversible redox switching of iCCC-DRM, suppressing the notorious factor of catalyst NPs sintering and carbon deposition (Supplementary Fig. 27b).

In summary, rationally combining the two strategies of the intelligent exsolution-dissolution with the inherent redox iCCC processes is bound to bring about extraordinary synergistic interactions. We demonstrated this proposal by developing perovskite-type LaNi_1-*x*_Al_*x*_O_3_/CaO DFMs for efficient and durable redox iCCC-DRM. From a crystallographic perspective, the substitution of Ni^2+^/Ni^3+^ by smaller Al^3+^ in B-site of perovskite mainly stabilizes the perovskite lattice to inhibit excessive Ni agglomeration, while Ni exsolution and re-integration are driven by B-site Ni valence change under alternating redox atmosphere. Apparently, the Ni^2+^/Ni^3+^ molar ratio was significantly enlarged, readily facilitating the formation of abundant O_v_s to balance the charges. As a result, O^2−^ ion migration channels of LaNi_1-*x*_Al_*x*_O_3_ perovskite were tailored in the conjunct CaO for fast CO_2_ adsorption. More significantly, Ni NPs are freely exsolved in the reduction DRM convention step and resolution in the tandem oxidation CO_2_ capture step owing to the preserved intact LaNi_1-x_Al_x_O_3_ perovskite structure. By tailoring the surface chemical potential and the adhesive energy between exsolved Ni NPs and the LaNi_1-x_Al_x_O_3_/CaO DFM through exsolution height and contact angle, the CH_4_ activation can be precisely matched with the DRM of CO_2_, successfully overcoming the bottlenecks of deactivation of Ni sintering and carbon deposit, simultaneously. Specifically, after the optimization, LaNi_0.8_Al_0.2_O_3_/CaO DFM achieved a stable CO_2_ and CH_4_ conversions of 91.5% and 93.5%, respectively, along with a high CO_2_ capture capacity of 10.2 mmol g^−1^ during 50 consecutive iCCC-DRM cycles. We anticipate that our findings regarding the reversible exsolution-dissolution strategy of perovskite-type DFMs will guide the design of highly durable intelligent catalysts, particularly for the redox iCCC processes.

## Methods

### Chemicals

Calcium nitrate tetrahydrate (Ca(NO_3_)_2_·4H_2_O, > 99%), nickel nitrate hexahydrate (Ni(NO_3_)_2_·6H_2_O, >99%), aluminium nitrate nonahydrate (Al(NO_3_)_3_·9H_2_O, >98%), Cerium nitrate hexahydrate (La(NO_3_)_3_) ·6H_2_O and citric acid monohydrate (C_6_H_10_O_8_, 99.99%) were purchased from Sinopharm. All chemicals were used directly without purification. Deionized water was used in all synthesis and washing processes.

### Synthesis of perovskites LaNi_1-x_Al_x_O_3_/CaO DFMs

Perovskite LaNi_1-*x*_Al_*x*_O_3_/CaO DFMs (*x* = 0, 0.1, 0.2, 0.3 and 0.5) were synthesized by sol-gel method. Adequate amount of Al(NO_3_)_3_·9H_2_O, Ni(NO_3_)_2_·6H_2_O, Ca(NO_3_)_2_·4H_2_O, La(NO_3_)_3_) ·6H_2_O and citric acid (molar ratio of citric acid to metal cation was 1:1) were added into 20 mL of deionized H_2_O with thoroughly stirring for 0.5 h at room temperature (Molar ratio: La^3+^: Ni^2+^: Al^3+^: Ca^2+^=1: (1-x): x :10). The solution was then evaporated to translucent pale green gel at 90 °C with constant stirring, followed by aged at 120 °C in oven overnight. The foamy sample was then ground and calcined at 800 °C for 2 h with a heating rate of 5 °C min^−1^ in stair air. For comparing, Ni/CaO DFM was also synthesized by using the impregnation method without the introduction of La and Al, and the Ni content was maintained at 5 wt%. The Al-doped LaNiO_3_ (LaNi_0.8_Al_0.2_O_3_) catalyst and CaO sorbent were synthesized by the sol-gel method, respectively. And subsequently physically mixed them together to keep Ni loading at about 5.0 wt%, namely LaNi_0.8_Al_0.2_O_3_/CaO-PM DFM.

### Characterizations

The power X-ray diffraction (D8 Advance, Bruker) operated at 40 kV and 40 mA and the pattern was recorded in the range of 10–80°. The materials were characterized by isothermal N_2_ adsorption-desorption at 77 K on Micromeritics ASAP-2020. The surface area and pore size distribution were calculated by the Brunauer-Emmett-Teller (BET) and Barrett-Joyner-Halenda (BJH) methods using the desorption branch, respectively. The metal content of the DFMs was determined by inductively coupled plasma-optical emission spectrometry (ICP-OES, Agilent ICPOES 730) after nitric acid digestion. Scanning electron microscopy (SEM) was conducted on a Helios G4 UC SEM-FIB (15 kV). Samples were pre-treated via Pt sputtering before the observation. Transmission electron microscopy (TEM) and scanning transmission electron microscopy (STEM) images were performed on a Thermo-Fisher Talos F200X (FETEM, 200 kV) equipped with an energy dispersive spectrometer (EDS) for determining the elemental distribution. High angle annular dark field (HAADF)-STEM images were recorded by using a convergence semi angle of 11 mrad and inner and outer collection angles of 59 and 200 mrad, respectively. X-ray photoelectron spectroscopy (XPS) of the samples was recorded on a Thermo-Scientific K-Alpha Spectrometer. All peaks were corrected by setting the C 1 s peak of 284.6 eV as the reference. Room-temperature electron paramagnetic resonance (EPR) was carried out on Bruker EMX plus. Carbon deposition on spent DFMs was characterized using Raman spectrometer (Thermo Scientific DXR2) equipped with a 532 nm diode laser (10 mW). The spectrums of each material were generated from three different sample areas.

Hydrogen and methane temperature-programmed reduction (TPR) was performed by Auto Chem 2720 Chemisorbent. Typically, 50 mg of the calcined sample was loaded in the sample tube and then heated to 300 °C in the nitrogen atmosphere (50 mL min^−1^) fo1h. After then the temperature was reduced to 50 °C, and the gas was switched to 10 vol.% H_2_ balanced with Ar (50 mL min^−1^). Subsequently, the temperature was raised from 50 °C to 800 °C with a heating rate of 10 °C min^−1^. TCD was used to detect the change of hydrogen consumption during the reduction process. The test process of CH_4_-TPR was similar to that of H_2_-TPR.

CO_2_-temperature programmed desorption (TPD) process was performed by the Auto Chem 2720 Chemisorbent. Typically, 50 mg of the sample was pretreated at 200 °C in N_2_ atmosphere for 0.5 h. After cooling to 50 °C, the gas was switched to CO_2_ for adsorption of 0.5 h, and then purged with He for 1 h. Under He atmosphere, the temperature was raised from 50 °C to 800 °C with a heating rate of 10 °C min^−1^.

The temperature programmed surface reaction (TPSR) was performed on Autochem 2720. The performance of the CO_2_ capture process, the CH_4_ dehydrogenation, and the integrated CO_2_ capture and conversion of iCCC-DRM processes was investigated by the CO_2_-TPSR, CH_4_-TPSR, and CH_4_-TPSR with CO_2_ pre-adsorption, respectively. Prior to each test, the sample (0.1 g) was pretreated under 10 vol.% H_2_ balanced with N_2_ at 700 °C for 30 min. For the CO_2_ capture process determined by the CO_2_-TPSR test, samples were heated from 200 to 800 °C with a heating rate of 10 °C min^−1^ under the gas flow of 10 vol.% CO_2_ balanced with N_2_ at a rate of 50 ml min^−1^. For the CH_4_ dehydrogenation process determined by the CH_4_-TPSR test, the operation conditions are the same except the gas flow changed into 5 vol.% CH_4_ balanced with N_2_. For the iCCC-DRM processes, the sample was exposed to a gas flow of 10 vol.% CO_2_ balanced with N_2_ at a rate of 50 ml min^−1^ for 30 min at 650 °C to make DFMs saturated with CO_2_. Then the sample was cooled down to 50 °C. Afterwards, the CH_4_-TPSR test was carried out under the same conditions as above. The nondispersive infrared analyzer (Smart Pro, Shandon) was used to monitor the concentration changes of each substance (CO, CO_2_, CH_4_ and H_2_) participated during all TPSRs in effluents.

### CO_2_ capture and in-situ conversion test

The CO_2_ capture and in-situ conversion (iCCC-DRM processes) were conducted in one fixed-bed column. The flow rates of N_2_, CO_2_, H_2_ and CH_4_ were controlled by the mass flow controllers (Horiba Metron), respectively. The products in the outlet gas were analyzed by the gas chromatography (GC, Haixin 950) with a thermal conductivity detector (TCD) and a flame ionization detector (FID). In addition, an in-situ nondispersive infrared analyser (Smart Pro, Shandon) was used to monitor the concentration changes of CO, CH_4_, H_2_ and CO_2_ continuously. In a typical experiment, approximately 0.1 g DFMs was added to a quartz tube (*Φ*10 × 150 mm) and packed with height of about 10 mm, then placed in the reactor furnace. The first step was the sample prereduction, which was carried out at 700 °C in the gas flow of 10 vol.% H_2_ balanced with N_2_ at a rate of 50 ml min^−1^ for 2 h. The second step was the CO_2_ capture, in which the gas was switched to the simulated flue gas of 10 vol% CO_2_/2 vol%O_2_ balanced with N_2_ at 50 ml min^−1^ (WHSV = 30 L g^−1^ h^−1^) and at a specific temperature such as 625, 650, 675, and 700 °C The third step was the purge. The pipeline was purged with pure N_2_ for 5 min. The fourth step was the in-situ conversion, in which the temperature was kept the same as in CO_2_ capture, and the gas was switched to 5 vol.% CH_4_ balanced with N_2_ at a flowrate of 50 ml min^−1^. Moreover, we performed the blank test in the same fixed bed under the same operating conditions by using an inert material (SiO_2_) with similar particle size to that of DFMs.

The CO_2_ adsorption capacity (*q)* was calculated by Eq. (1).

1 $$q=\frac{{\int }_{0}^{{{{\rm{ts}}}}}[{F}_{{{{{\rm{CO}}}}}_{2},{{{\rm{in}}}}}-{F}_{{{{{\rm{CO}}}}}_{2},{{{\rm{out}}}}}({{{\rm{t}}}})]dt}{{M}_{0}}$$

where *F*_CO2,in_ is the CO_2_ molar flow rate in the inlet gas of the simulated flue gas, *F*_CO2,out_ is the CO_2_ molar flow rate in the outlet gas, *t*s is the duration time of the capture step, and *M*_0_ is the sample mass.

The CO_2_ and CH_4_ conversion efficiency was calculated by Eq. (2).

2 $${{{{\rm{X}}}}}_{i}=\frac{{\int }_{0}^{{{{\rm{ts}}}}}[{F}_{{{{\rm{i}}}},{{{\rm{in}}}}}-{F}_{{{{\rm{i}}}},{{{\rm{out}}}}}({{{\rm{t}}}})]dt}{{{{{\rm{F}}}}}_{{{{\rm{i}}}},{{{\rm{in}}}}}\times ts}\times 100\%$$

where X_i_ (%) is the conversion of CO_2_ or CH_4_, *F*_i,in_ and *F*_i,out_, represent the molar flow rate of CO_2_ or CH_4_ in the inlet gas and the outlet gas, respectively. *t*s is the duration time of the in-situ conversion step.

The mass activity of the average conversion rate (*R*_i_, mmol s^−1^ g_Ni_^−1^) of CO_2_ or CH_4_ in terms of the mass of Ni loading is calculated by the following Eq. (3).

3 $${R}_{i}=\frac{{\int }_{0}^{{{{\rm{ts}}}}}[{F}_{{{{\rm{i}}}},{{{\rm{in}}}}}-{F}_{{{{\rm{i}}}},{{{\rm{out}}}}}({{{\rm{t}}}})]dt}{{m}_{{\mbox{Ni}}}\times ts}$$

Where *F*_i,in_ and *F*_i,out_ represents the molar flow rate of CO_2_ or CH_4_ in the inlet and outlet gas, *t*_s_ is the duration time of the in-situ conversion step, and *m*_*Ni*_ is the mass of Ni loading in DFMs.

The H_2_/CO molar ratio (*R*_H/C_) of syngas was determined by the ratio between H_2_ and CO concentrations in the outlet gas.

Kinetics analysis of iCCC-DRM and the calculation of interfacial adhesion energy (*E*_adh_) and chemical potential (*µ*) are provided in the Supplementary Information (SI).

## Supplementary information

Supplementary Information

Transparent Peer Review file

## Data Availability

The data that support the findings of this study are available within the article and Supplementary Information or from the corresponding authors upon request.
