# Supplementary material for: Self-adaptive Ni nanoparticles in perovskite LaNi1-xAlxO3/CaO for durable CO2 capture and in-situ conversion
Source: Nat Commun. 2026 Jul 21;17:8903. doi: 10.1038/s41467-026-75734-x (PMC13503938; doi:10.1038/s41467-026-75734-x)
Supplement: Supplementary file 1 [file 41467_2026_75734_MOESM1_ESM.pdf]

Supplementary Information for

Self-Adaptive Ni Nanoparticles in Perovskite $LaNi_{1-x}Al_xO_3$/CaO for Durable CO_2 Capture and in-situ Conversion

Bin Shao[1], Zhonghao Jia[1], Sheng Dai[1,2] & Jun Hu[1*]

Affiliations

[1] State Key Laboratory of Green Chemical Engineering and Industrial Catalysis, School of Chemistry and Molecular Engineering, East China University of Science and Technology, 130 Meilong Road, Shanghai, 200237, China.

[2] Key Laboratory for Advanced Materials and Feringa Nobel Prize Scientist Joint Research Centre, School of Chemistry and Molecular Engineering, East China University of Science and Technology, Shanghai 200237, China.

Corresponding authors

*Correspondence to: Jun Hu

*E-mails: junhu@ecust.edu.cn

Supplementary Methods

1.1 Adsorption kinetics

For $LaNi_{1-x}Al_xO_3/CaO$ DFMs, 650 °C was selected as the optimal adsorption temperature. The CO_2 adsorption curves of CaO-based adsorbents were fitted using the following double exponential model:

$$q = A_1 + A_2 \exp(-k_1 \times t) + A_3 \exp(-k_2 \times t) \tag{1}$$

where q denotes the CO_2 adsorption capacity, t represents the adsorption time, k_1 and k_2 are the rate constants corresponding to the reaction-controlled stage and diffusion-controlled stage, respectively; A_1 is a constant, while A_2 and A_3 are pre-exponential factors. The Levenberg-Marquardt algorithm was employed for the regression of parameters k_1, k_2, A_1, A_2 and A_3.

1.2 Kinetics details of in-situ DRM reaction

The conversion efficiency of adsorbed CO_2 (X) could be expressed as

$$X = \frac{0.5 n_{CO} + n_{CO_2,desorb}}{n_{CO_2,adsorb}} \tag{2}$$

Where n_{CO} and $n_{CO_{2,desorb}}$ were the moles of the produced CO and desorbed CO_2 from beginning to t minutes CO_2, respectively. $n_{CO_{2,adsorb}}$ was the moles of adsorbed CO_2 at the CO_2 capture step.

Here, we chose the nucleation and subsequent growth model (n=2) to describe the reaction of in-situ DRM reaction (namely, calcium carbonate reforming with methane). The function $f(X)$ was expressed by Avrami-Erofeev(A2) equation.

$$f(X) = 2(1-X)[-\ln(1-X)]^{\frac{1}{2}} \tag{3}$$

In which, X means the conversion efficiency of adsorbed CO_2 (X_{CO2}) with time. So the integral form of the dynamic equation could be expressed as:

$$[-\ln(1-X)]^{\frac{1}{2}} = kt \tag{4}$$

Where the k is the reaction constant.

1.3 Calculation of interfacial adhesion energy (E_{adh}) and chemical potential (μ)

The metal nanoparticles (NPs) are treated with the approximation of spherical shape, which simplifies the theoretical derivation process. Based on the spherical model with contact angle α (0° < α <180°), the observed radius of nanoparticles (NPs) (r) is smaller than the curvature radius (R), whose relationship is displayed as following:

$$\gamma = R \times \sin(\alpha) \tag{5}$$

Thus, the relationship between curvature radius (R) and observed diameter (NPs size, d) follows:

$$R = \frac{d}{2 \times \sin(\alpha)} \tag{6}$$

The adhesion energy ($E_{adh,}$ in J m^{-2}) of Ni was calculated according to the Young-Dupre equation.

$$E_{\text{adh}} = \gamma_{Ni}(R) \times (1 + \cos(\alpha)) \tag{7}$$

Where γ_{Ni} (R), R, and α represent the surface energy, curvature radius of Ni NP, and contact angle, respectively. The relationship between surface energy $\gamma(R)$ and curvature diameter R is described as follows. For Ni NPs, the surface energy γ_{Ni} (R) decreases with the increase in size due to the increase of surface coordination number C_{Ni}. By assuming a spherical model, the surface coordination number (C_{Ni}) can be formulated as following.

$$C_{Ni}(R) = \frac{1}{1.18R^{-2.28} + 0.13} \tag{8}$$

The surface energy γ_{Ni}(R) was further approximated as the following:

$$\gamma_{Ni}(R) = \frac{[C_{Ni}(R) - 12]}{24} \times \frac{\text{E}_{\text{c,Ni}}}{\text{S}_{\text{Ni}} \times \text{N}_{\text{A}}} \tag{9}$$

where $E_{c,Ni}$ is the cohesive energy of Ni and S_{Ni} is the area per Ni atom.

$$\mu(R) = \frac{2\Omega\lambda_{\text{Ni}}(R)}{R} \tag{10}$$

where μ is the chemical potential, and Ω is the bulk metal volume per atom.

Supplementary Figures and Tables

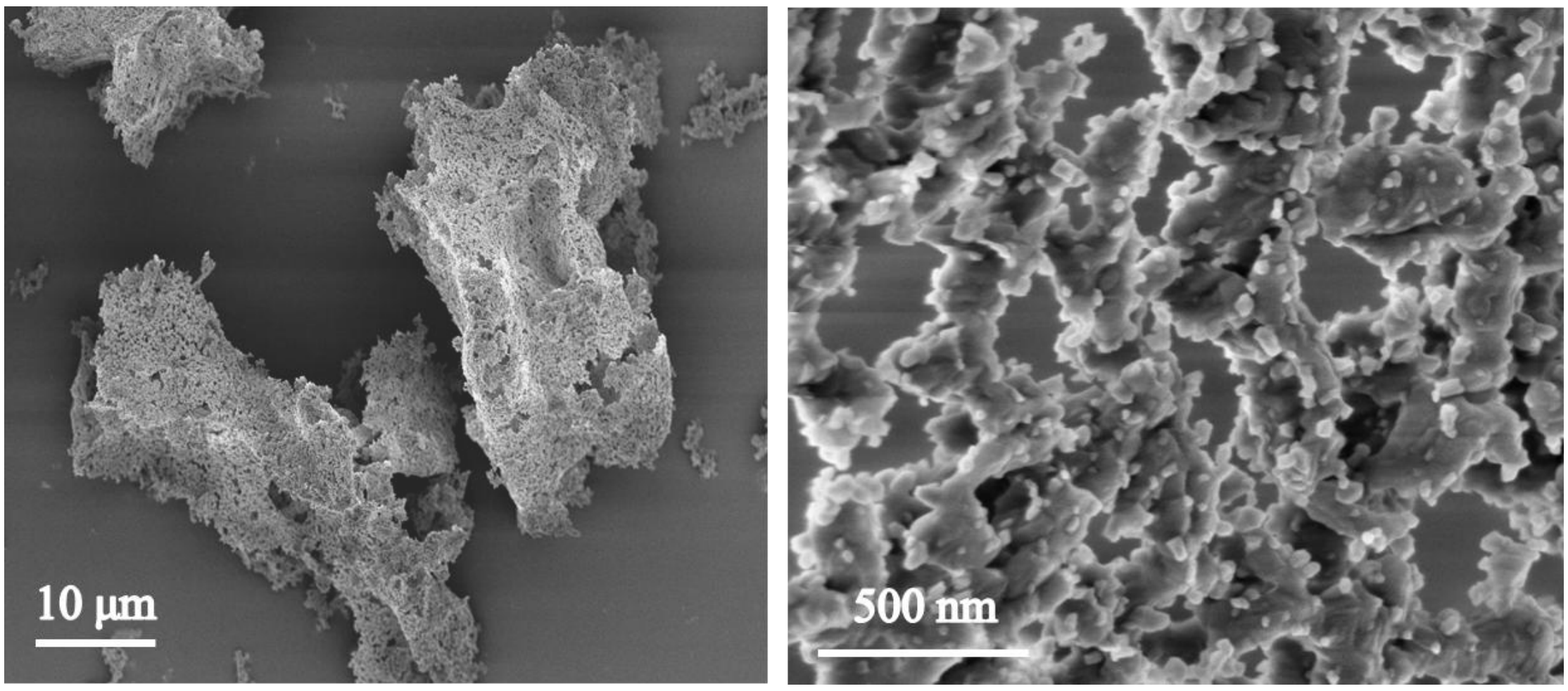

Supplementary Fig. 1 Morphological characterizations. SEM images of as-synthesized $LaNi_{0.8}Al_{0.2}O_3$/CaO DFM

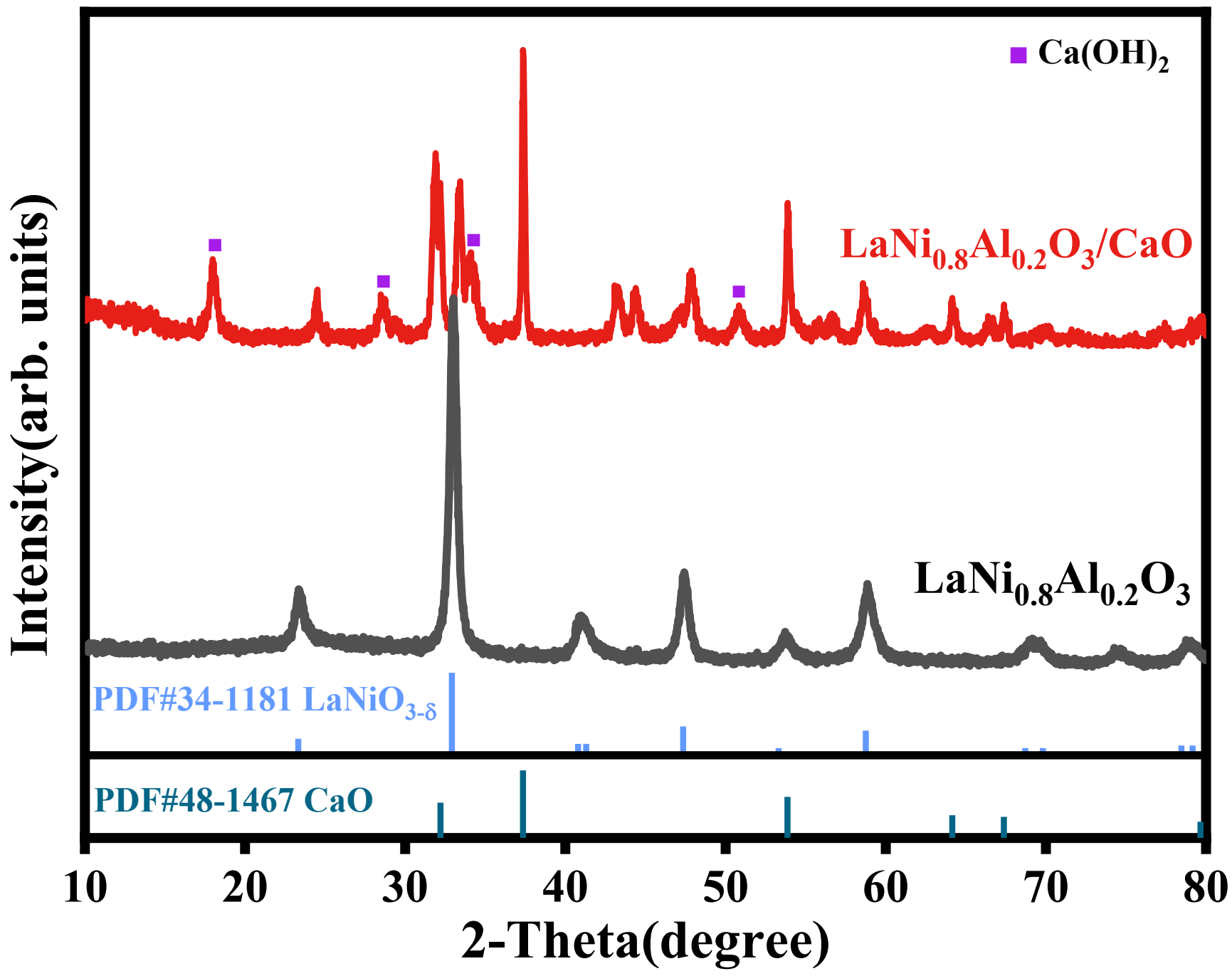

Supplementary Fig. 2 Structural characterizations. XRD patterns of the as-synthesized $LaNi_{0.8}Al_{0.2}O_3$ catalyst and $LaNi_{0.8}Al_{0.2}O_3$/CaO DFM.

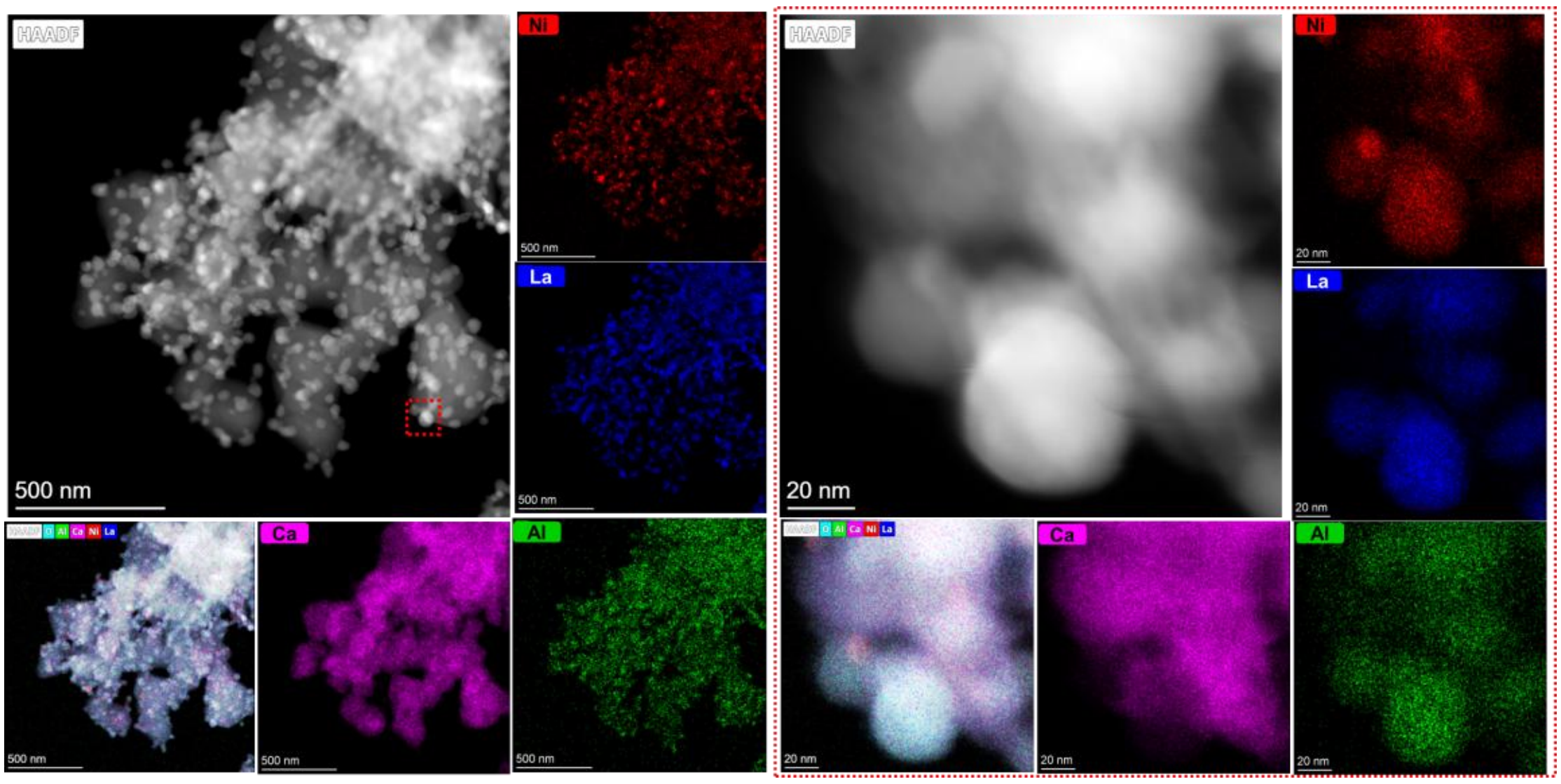

Supplementary Fig. 3 Morphological characterizations. STEM-HAADF and EDS elemental mapping images of the as-synthesized $LaNi_{0.8}Al_{0.2}O_3$/CaO DFM.

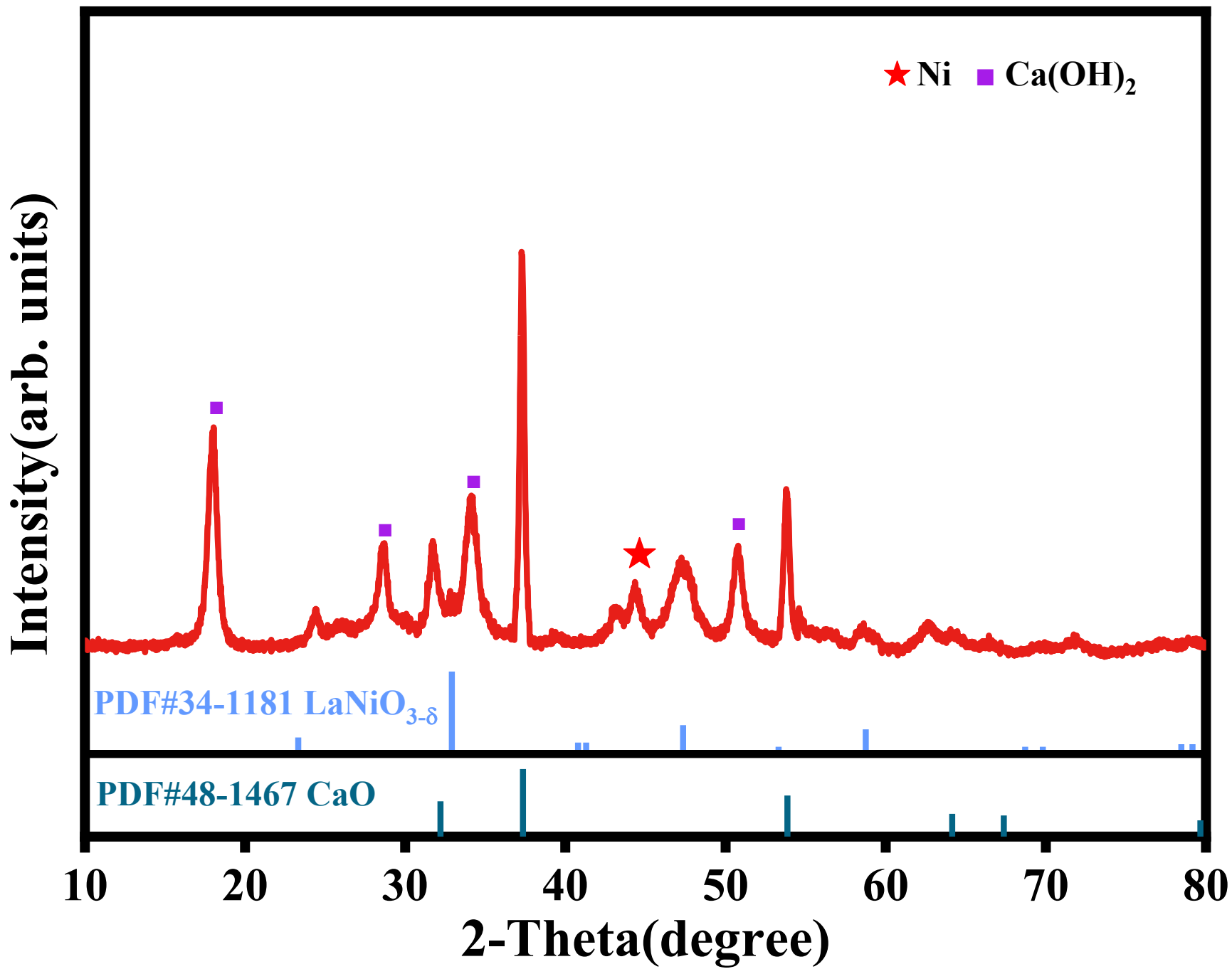

Supplementary Fig. 4 Structural characterizations. XRD patterns of the reduced $LaNi_{0.8}Al_{0.2}O_3$/CaO DFM at 700°C under hydrogen atmosphere.

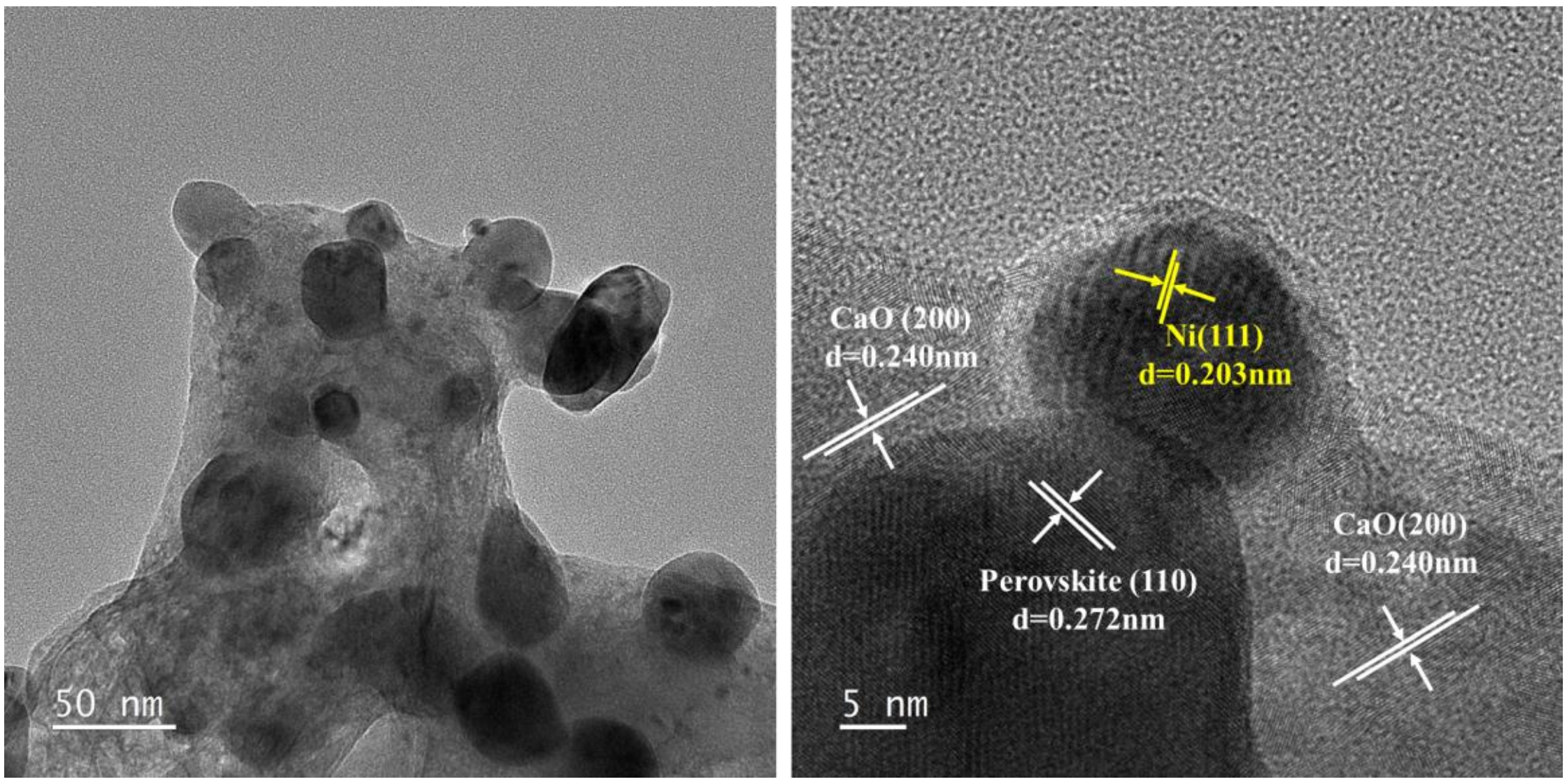

Supplementary Fig. 5 Morphological characterizations. HRTEM images of the reduced $LaNi_{0.8}Al_{0.2}O_3$/CaO DFM

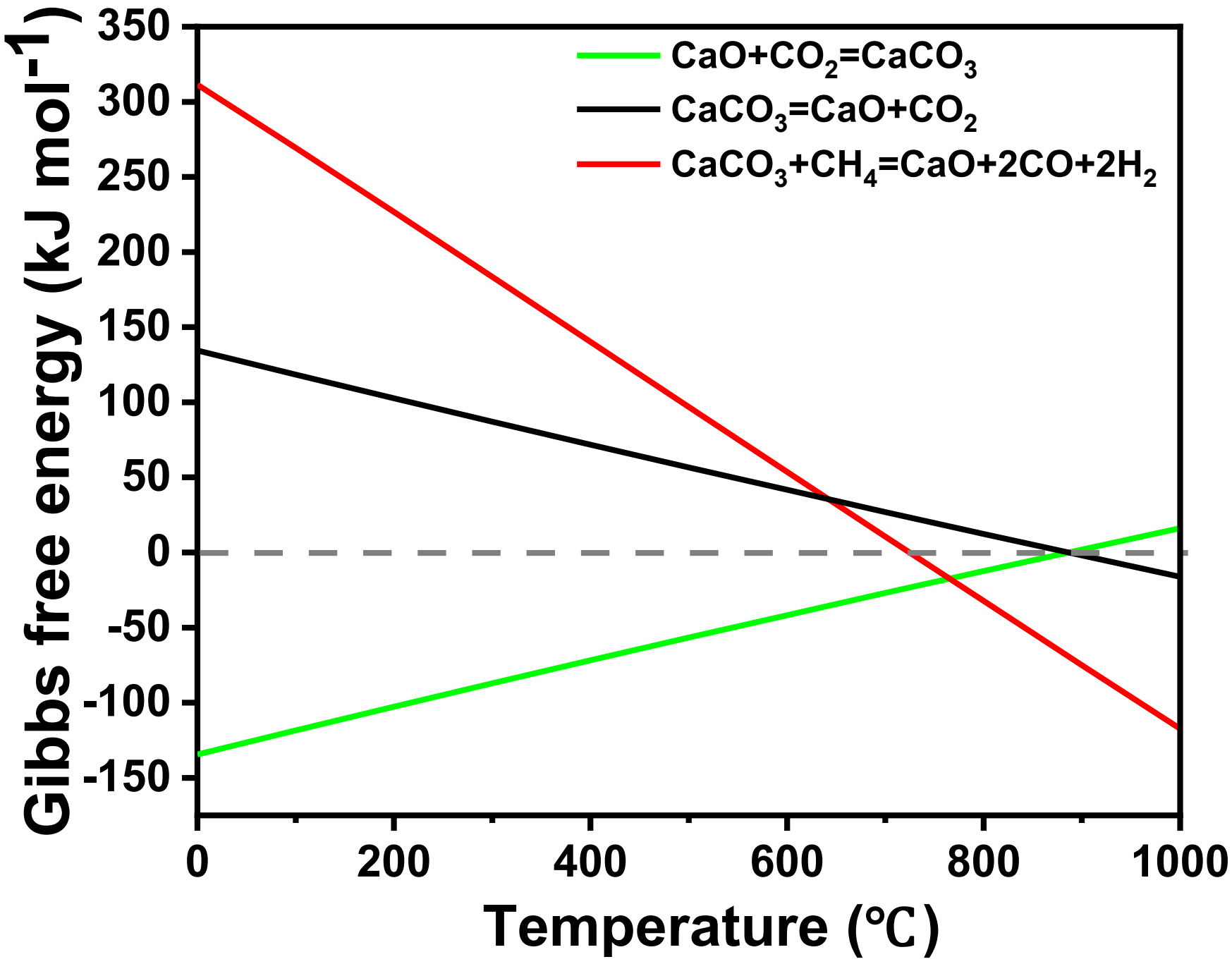

Supplementary Fig. 6 Thermodynamic calculation of the reaction. Gibbs free energy of the related reaction as a function of temperature.

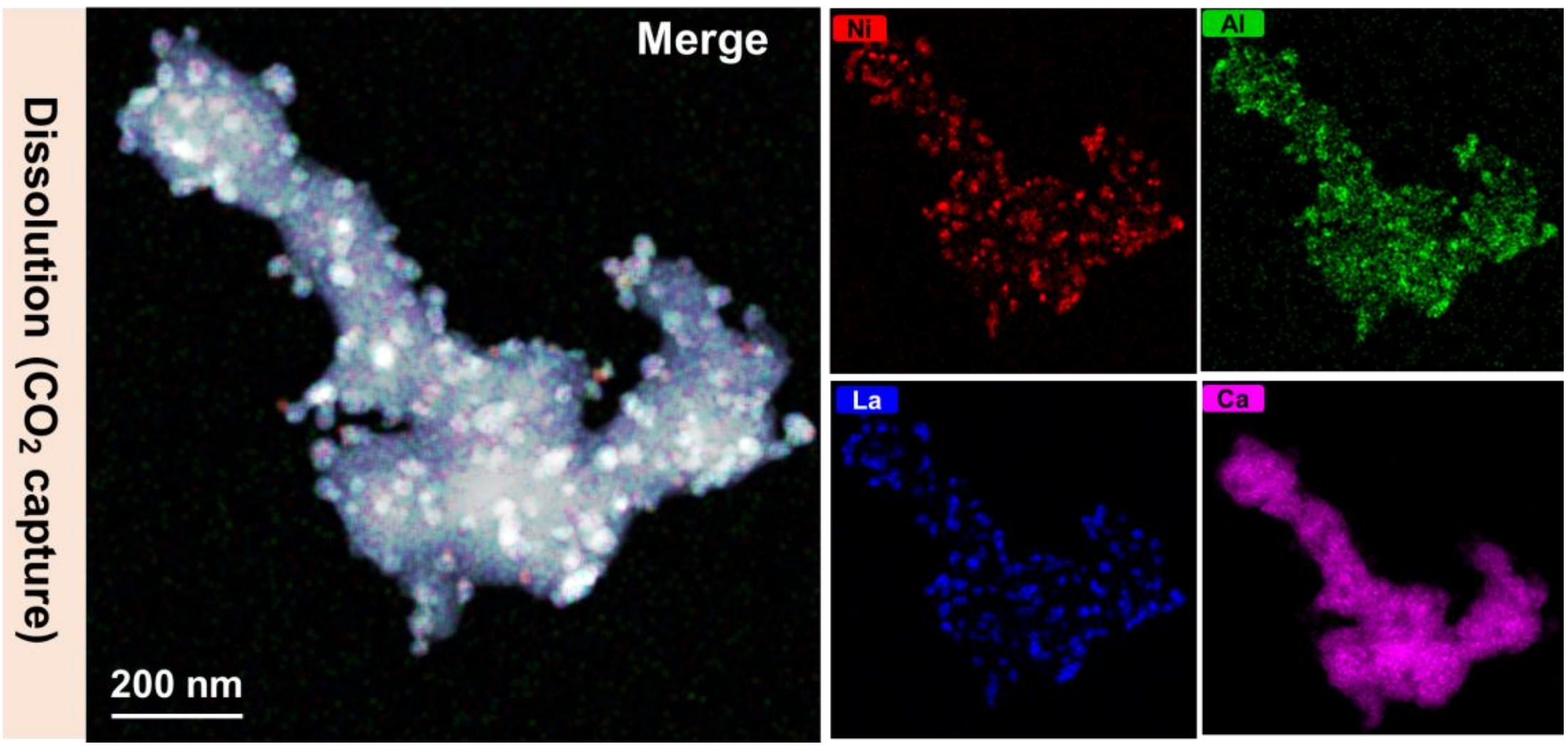

Supplementary Fig. 7 Morphological characterizations. STEM-HADDF images of $LaNi_{0.8}Al_{0.2}O_3/CaO$ DFM at the stages of CO_2 adsorption.

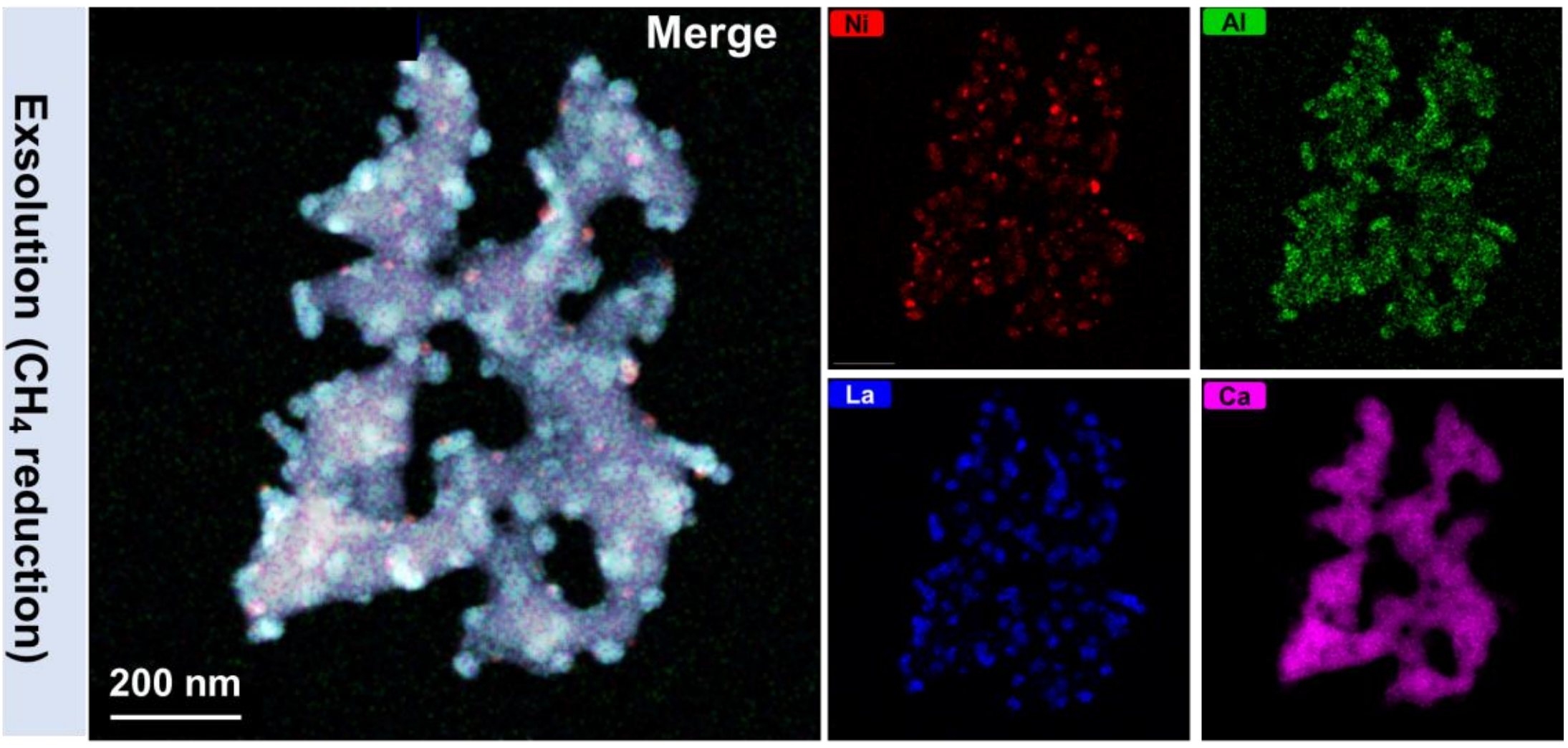

Supplementary Fig. 8 Morphological characterizations. STEM-HADDF images of $LaNi_{0.8}Al_{0.2}O_3$/CaO DFM at the stages of subsequently DRM conversion by CH_4.

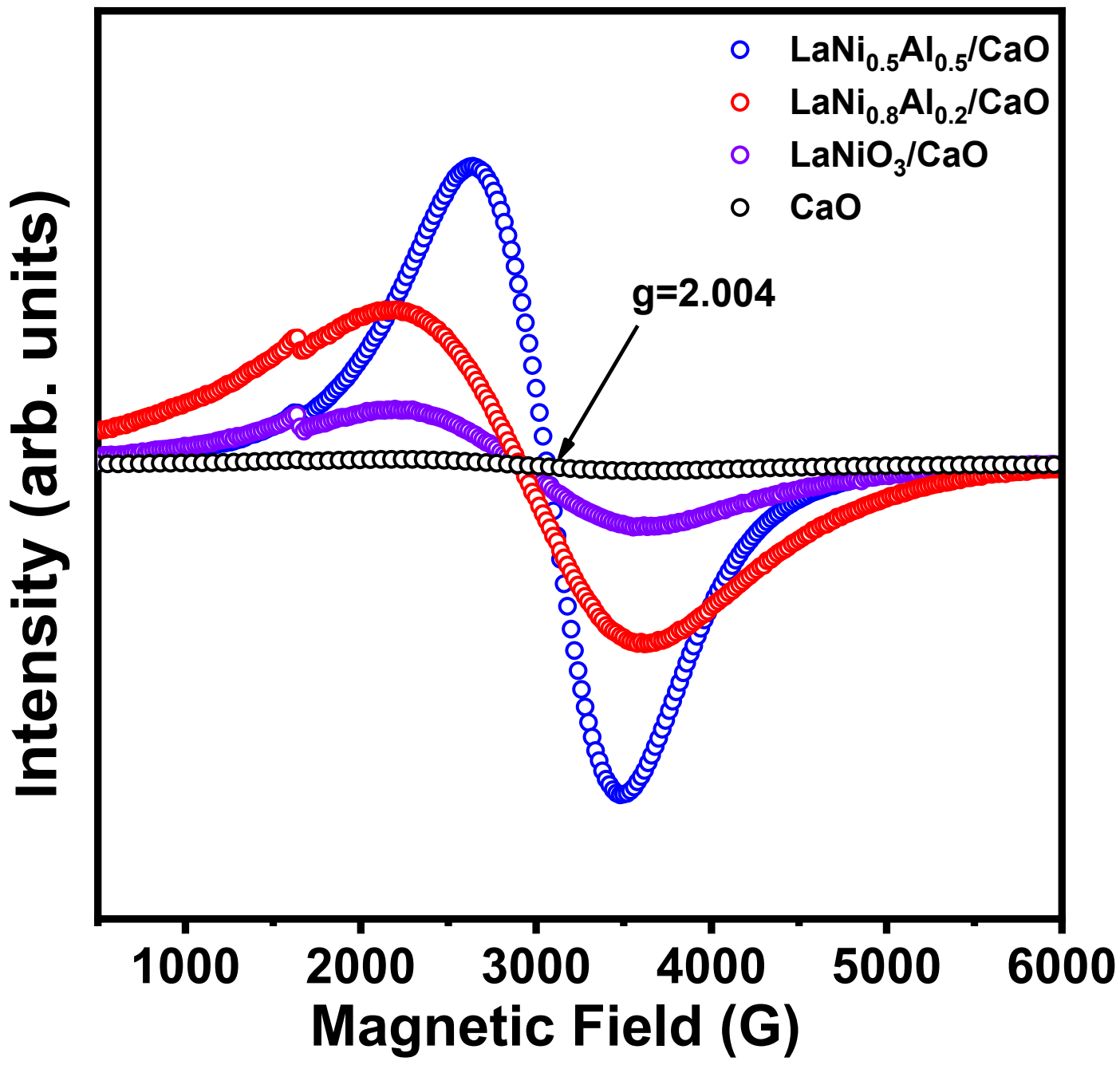

Supplementary Fig. 9 Structural characterizations. EPR curves of CaO sorbent and $LaNi_{1-x}Al_xO_3$/CaO DFMs.

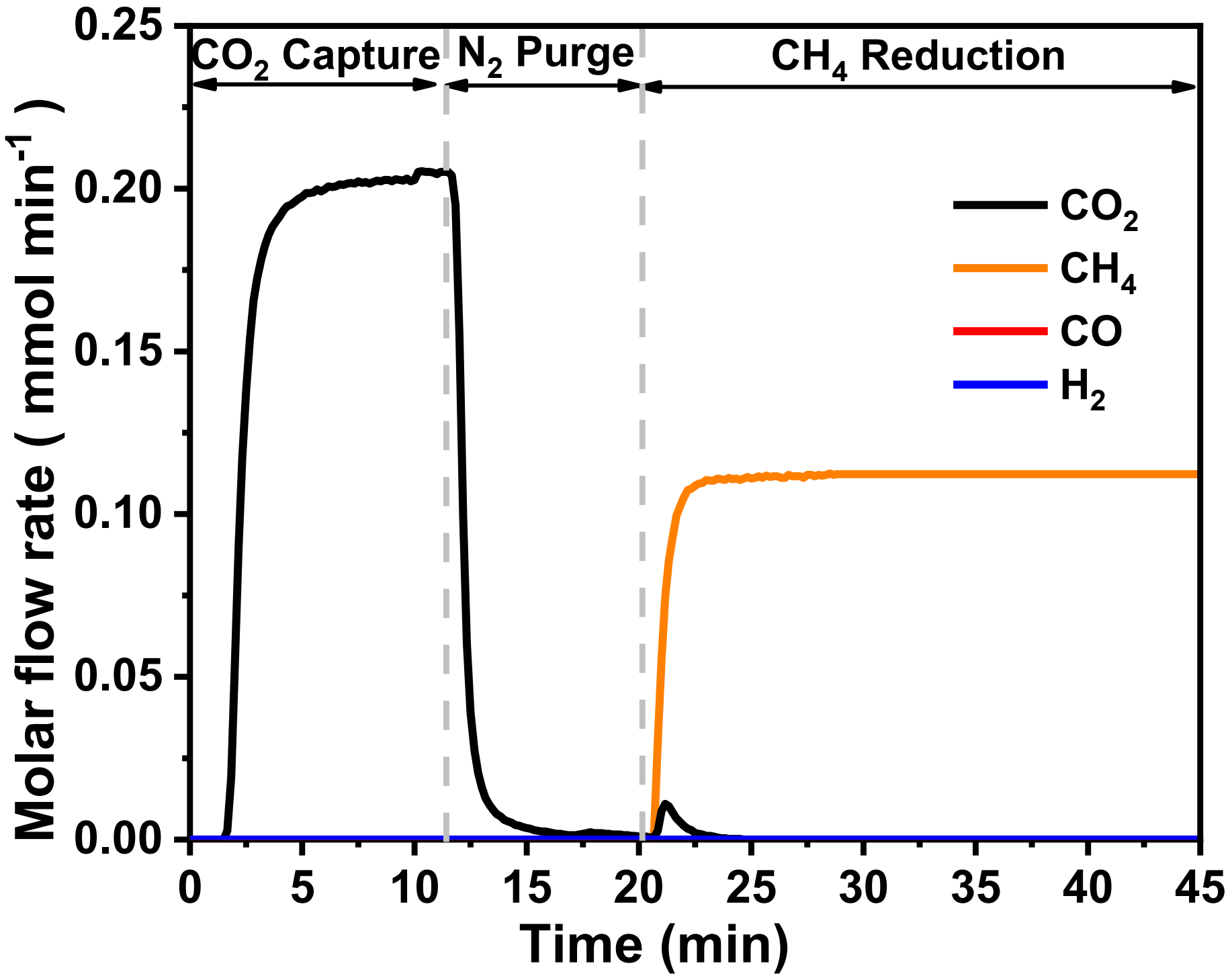

Supplementary Fig. 10 ICCC-DRM performance. The blank experiment of the iCCC-DRM process.

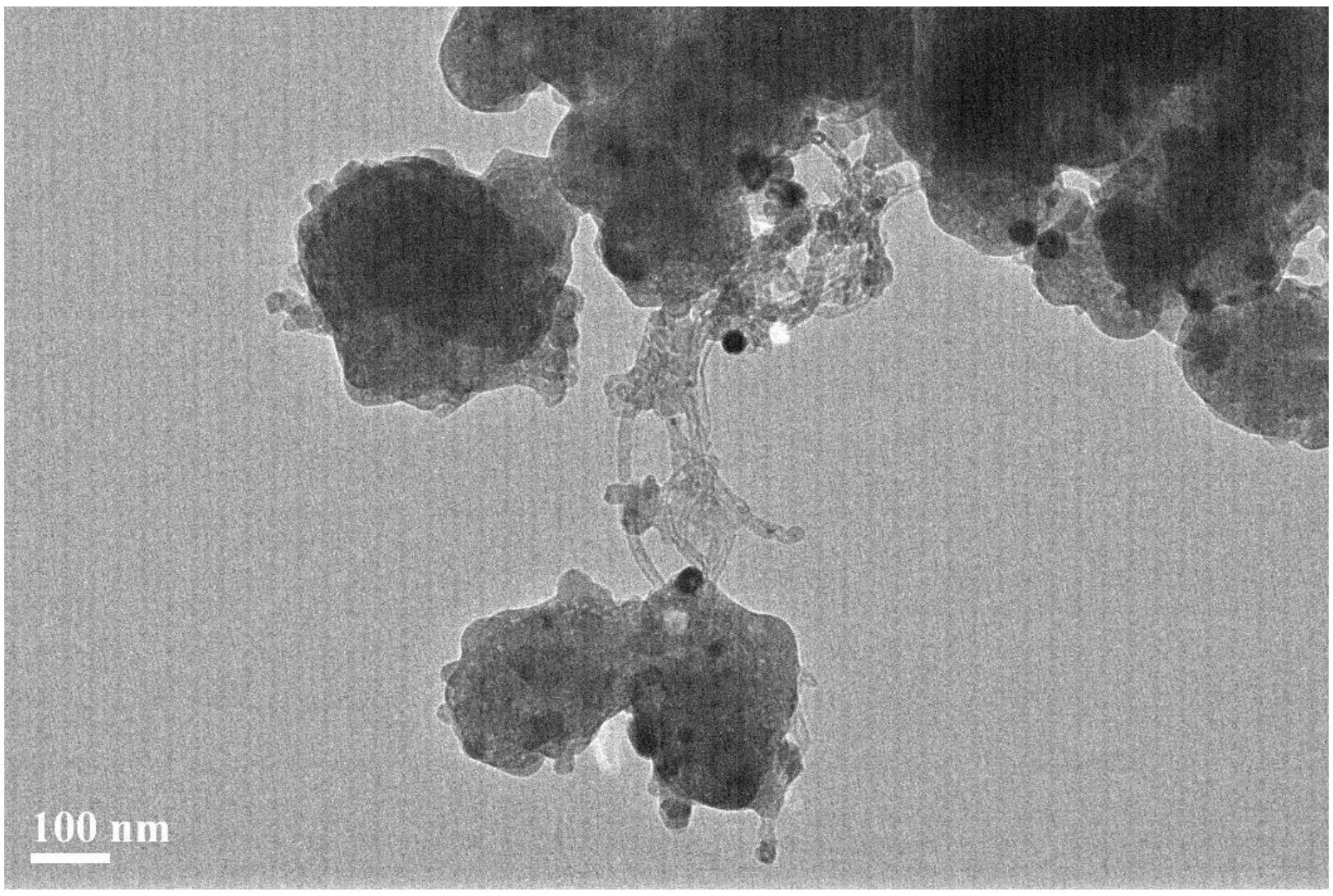

Supplementary Fig. 11 Morphological characterizations. TEM image of spent $LaNiO_3/CaO$ DFM after 25 cycles.

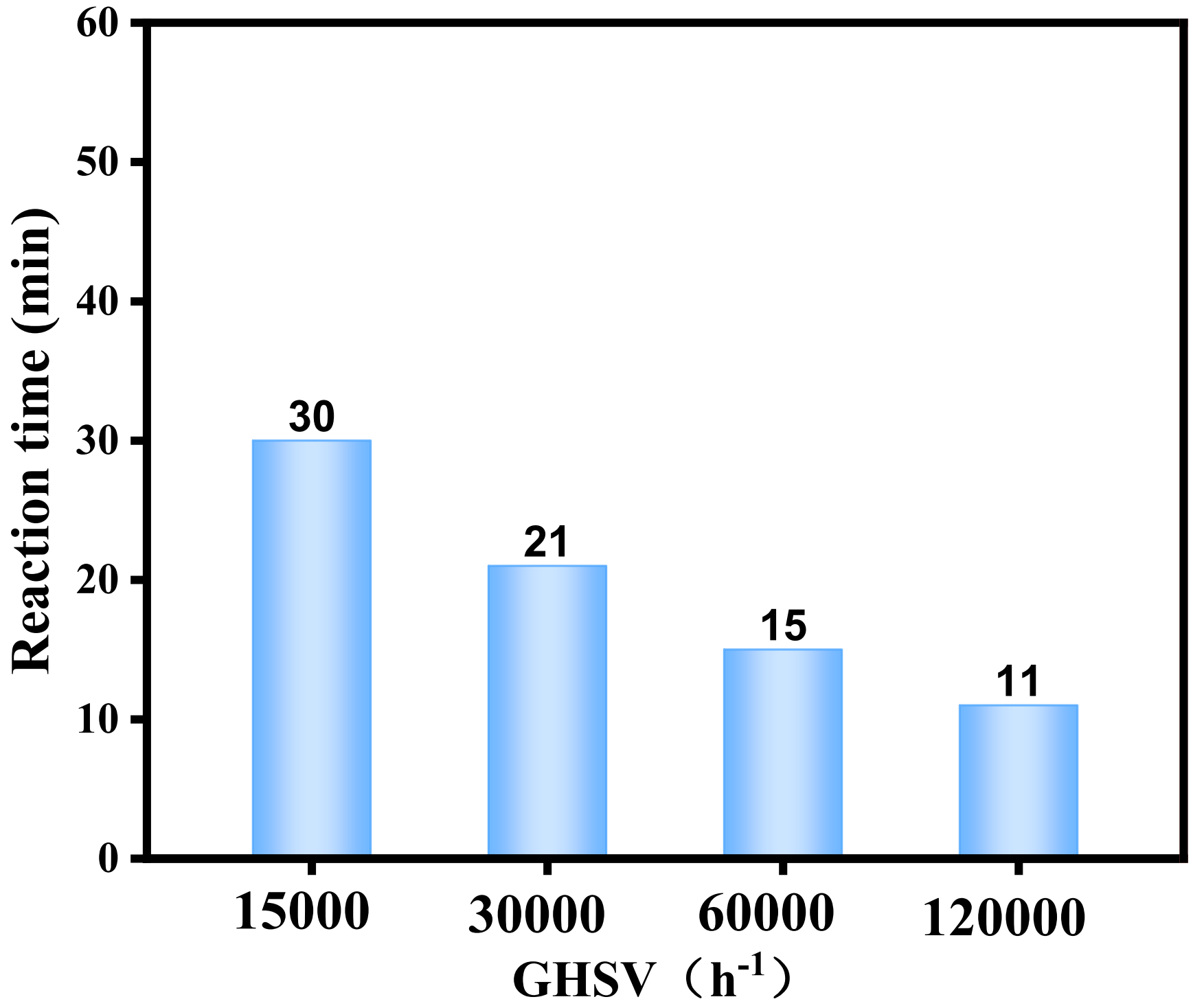

Supplementary Fig. 12 ICCC-DRM performance. The reaction time of in-situ DRM in iCCC-DRM process under various GHSVs from 15000 to 120000 h^{-1}.

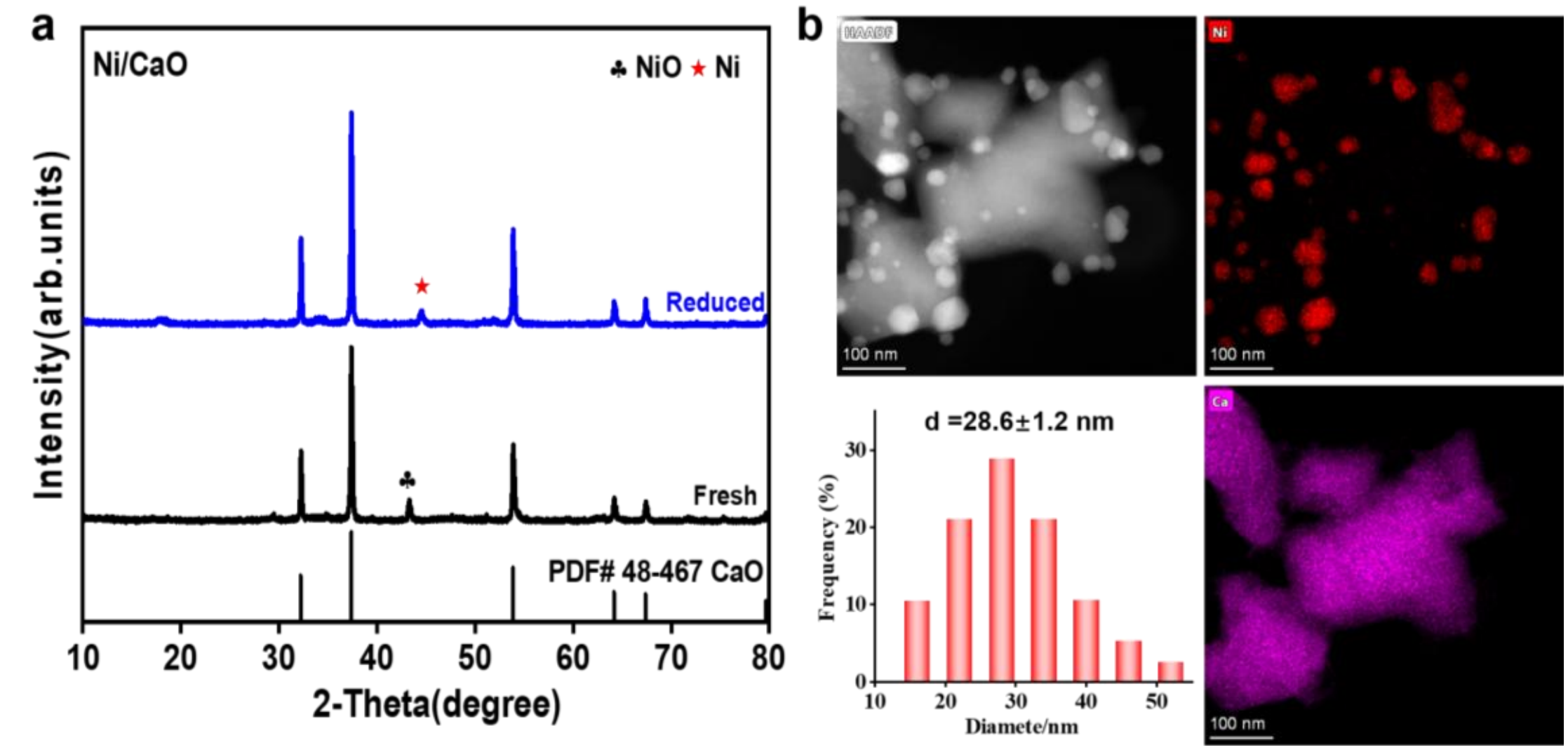

Supplementary Fig. 13 Structural and morphological characterizations. (a) XRD patterns of as-synthesized and reduced Ni/CaO DFM. (b) HAADF-STEM images and corresponding size distribution of reduced Ni/CaO DFM.

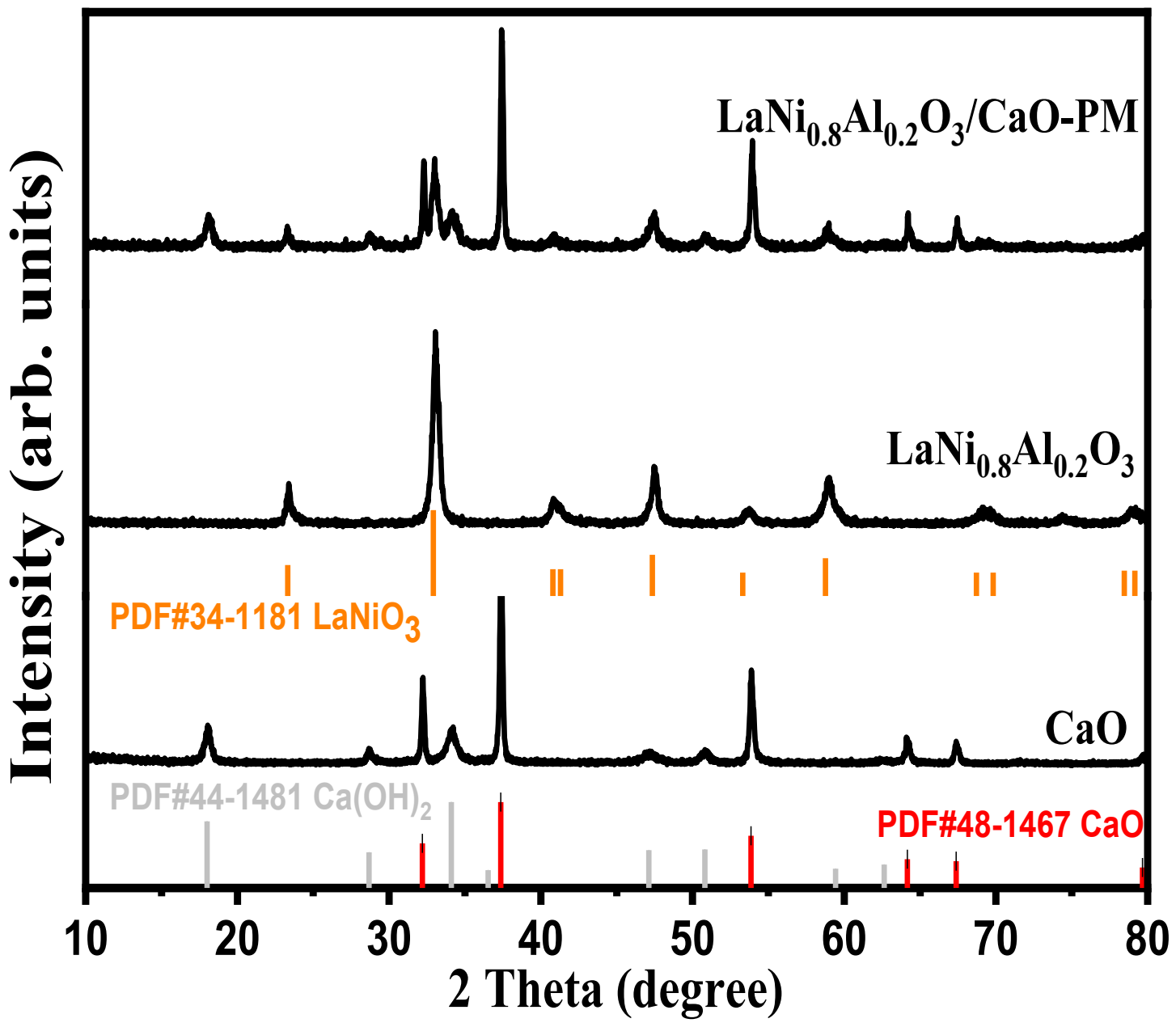

Supplementary Fig. 14 Structural characterizations. XRD patterns of as-prepared $LaNi_{0.8}Al_{0.2}O_3$ catalyst, CaO sorbent and the $LaNi_{0.8}Al_{0.2}O_3$/CaO-PM.

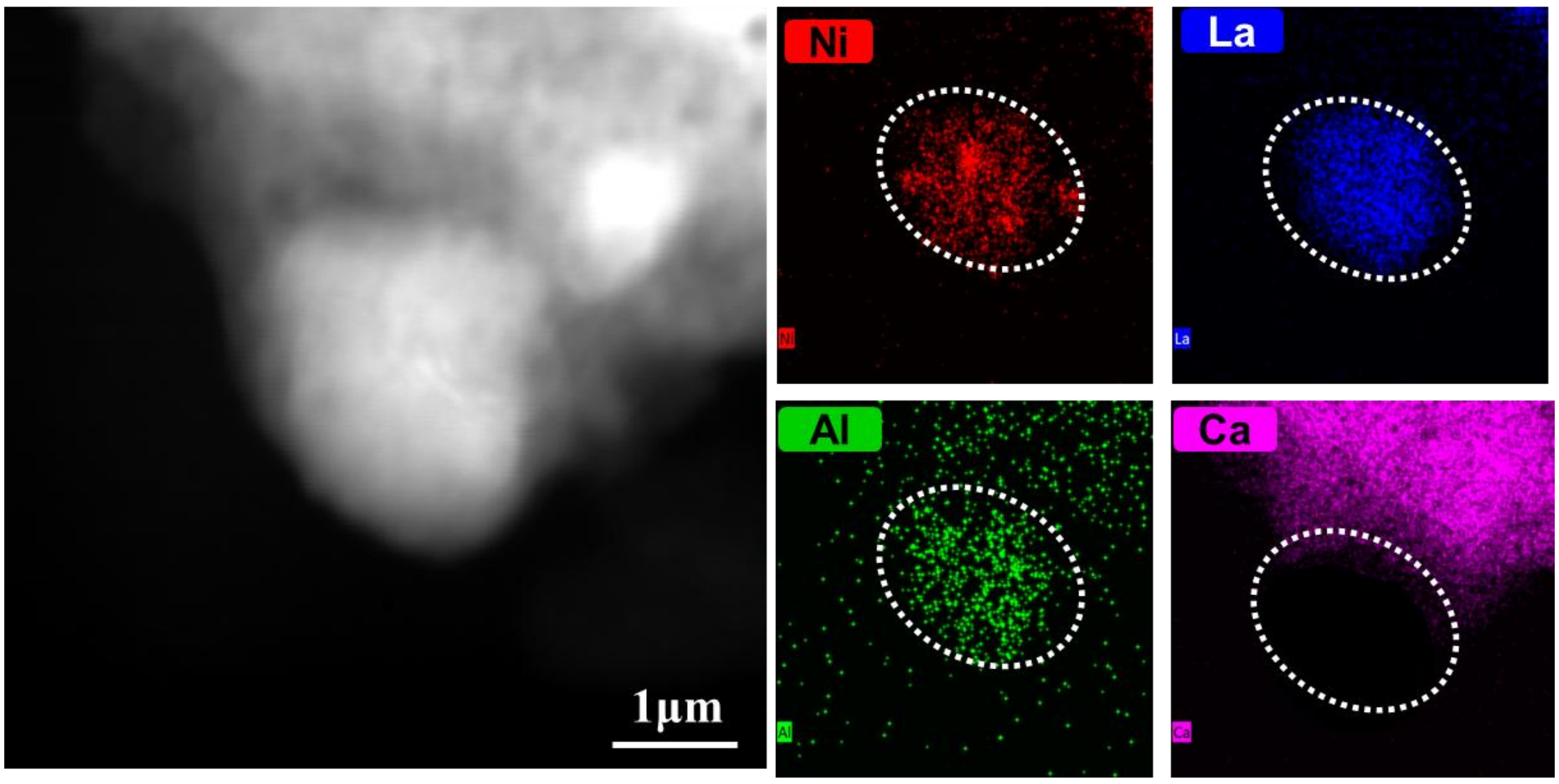

Supplementary Fig. 15 Morphological characterizations. STEM-HAADF image and EDS elemental mappings of the $LaNi_{0.8}Al_{0.2}O_3$/CaO-PM

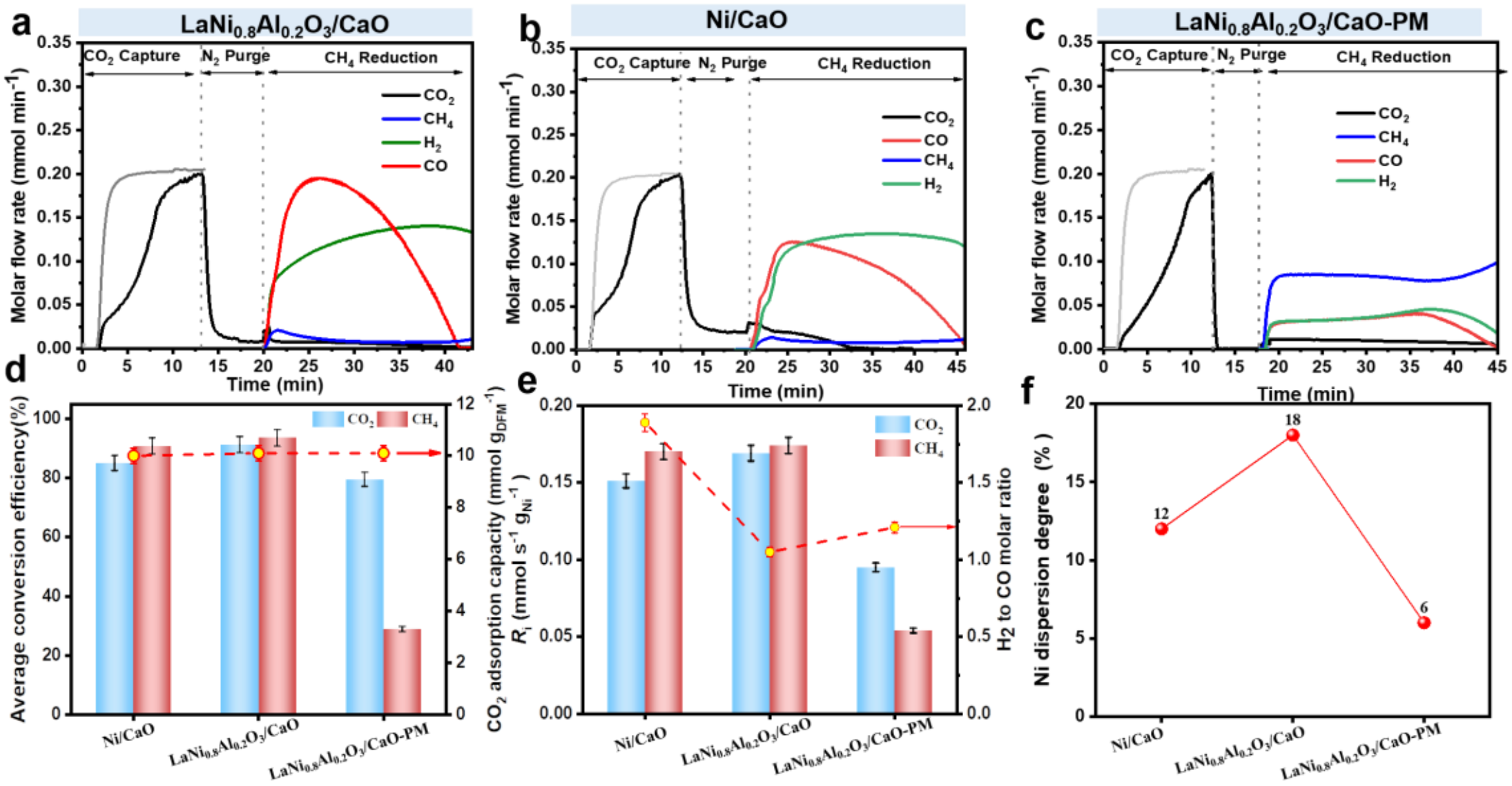

Supplementary Fig. 16 ICCC-DRM performance. Typical molar flow rate of each component in the effluent gas during one cycle of iCCC-DRM on (a) $LaNi_{0.8}Al_{0.2}O_3$/CaO, (b)Ni/CaO and (c) $LaNi_{0.8}Al_{0.2}O_3$/CaO-PM. (d) Average conversion efficiency of CO_2 and CH_4 along with CO_2 capture capacity, and (e) average conversion rate (R_i) of CO_2 and CH_4 along with the H_2-to-CO molar ratio on different DFMs. (f) The Ni dispersion degree of different DFMs. Error bars mean ±standard deviations calculated from three independent measurements

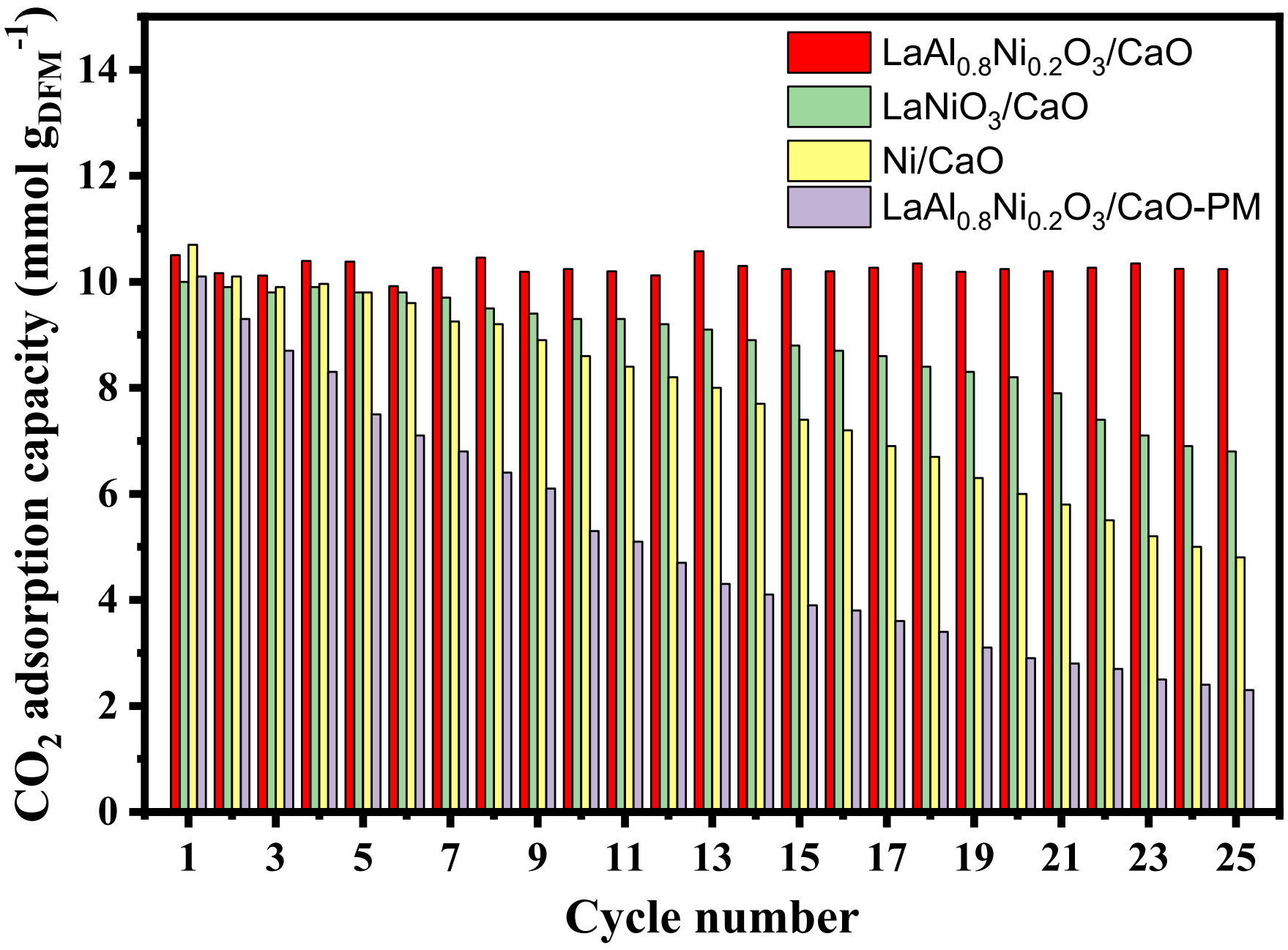

Supplementary Fig. 17 Cycling stability for CO_2 capture. CO_2 adsorption capacity of 25 cyclic iCCC-DRM processes on Ni/CaO, $LaNi_{0.8}Al_{0.2}O_3$/CaO and $LaNi_{0.8}Al_{0.2}O_3$/CaO-PM DFMs.

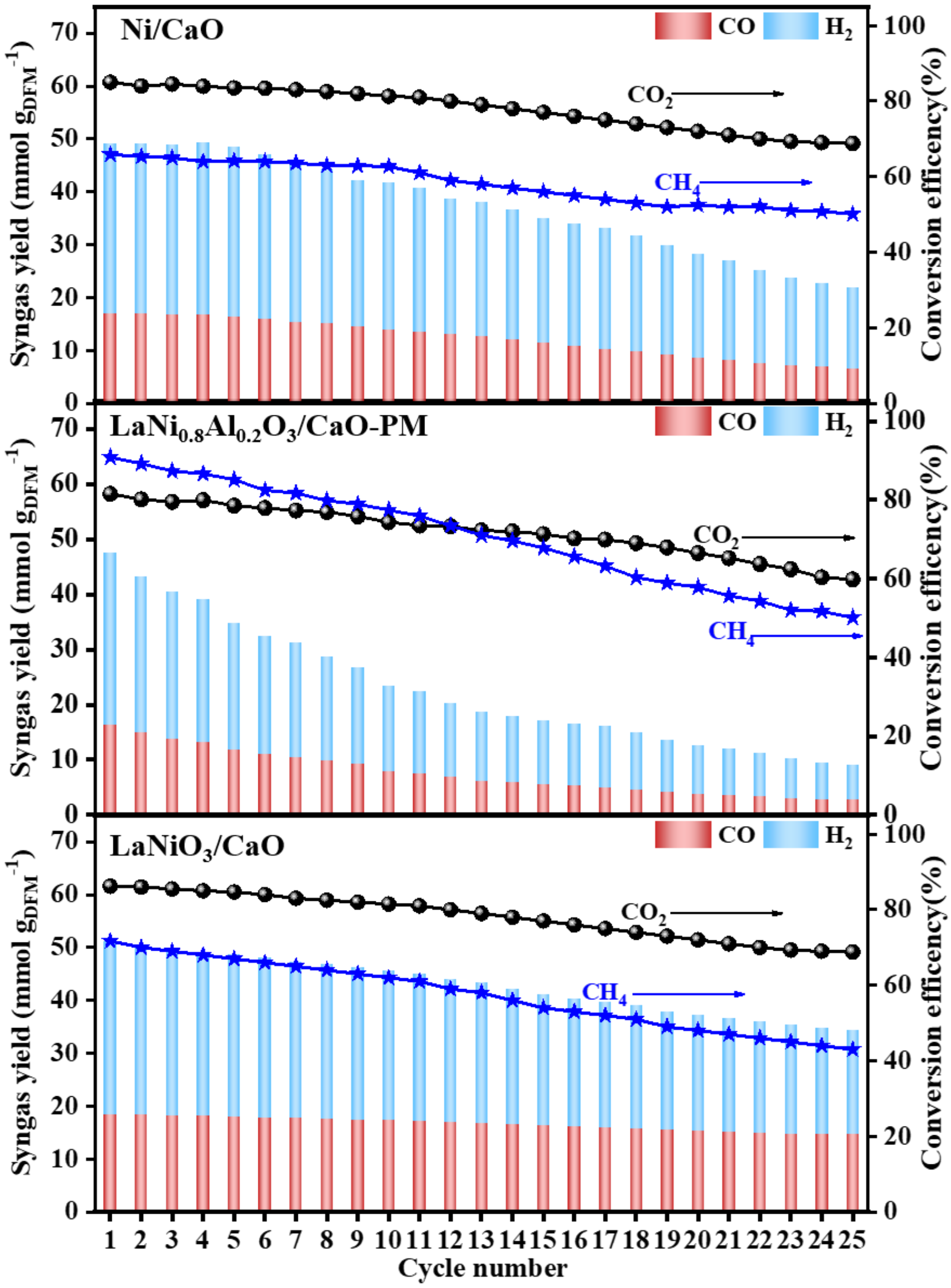

Supplementary Fig. 18 ICCC-DRM performance. 25 cyclic iCCC-DRM stability of Ni/CaO, $LaNiO_3$/CaO DFMs and $LaNi_{0.8}Al_{0.2}O_3$/CaO-PM at 650°C. Operating conditions: at the CO_2 capture stage, the inlet gas was 10 vol% CO_2 and 2 vol%O_2 simulated flue gas, after N_2 purging, the inlet gas at the conversion stage was switched into 5 vol% CH_4 at GHSV of 30000 mL g^{-1} h^{-1}.

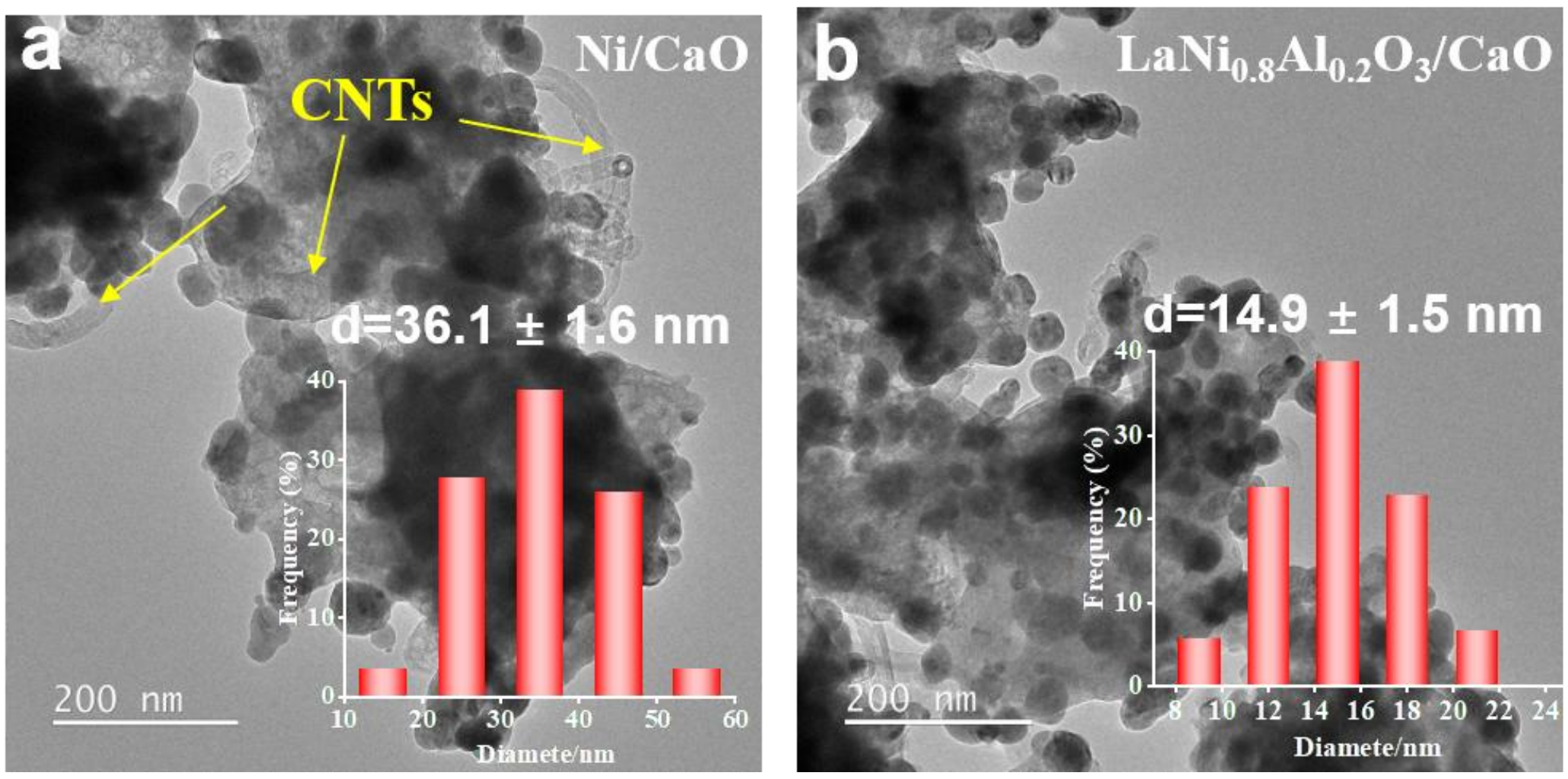

Supplementary Fig. 19 Morphological characterizations. (a)TEM image of spent Ni/CaO after 25 cycles; (b) TEM image of spent $LaNi_{0.8}Al_{0.2}O_3/CaO$ after 25 cycles.

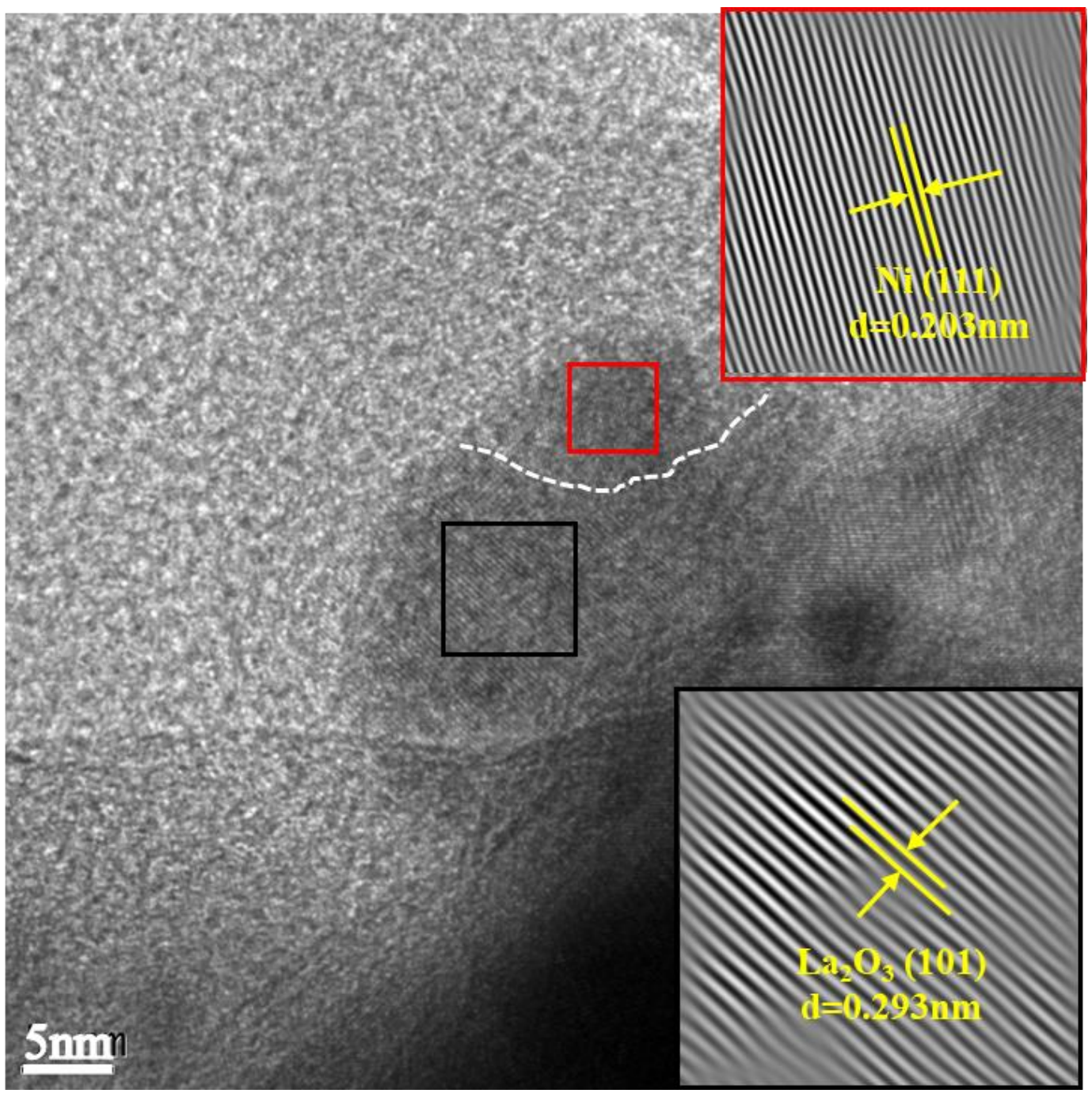

Supplementary Fig. 20 Morphological characterizations. HRTEM images of $LaNiO_3$/CaO DFM after 25th iCCC-DRM cycles.

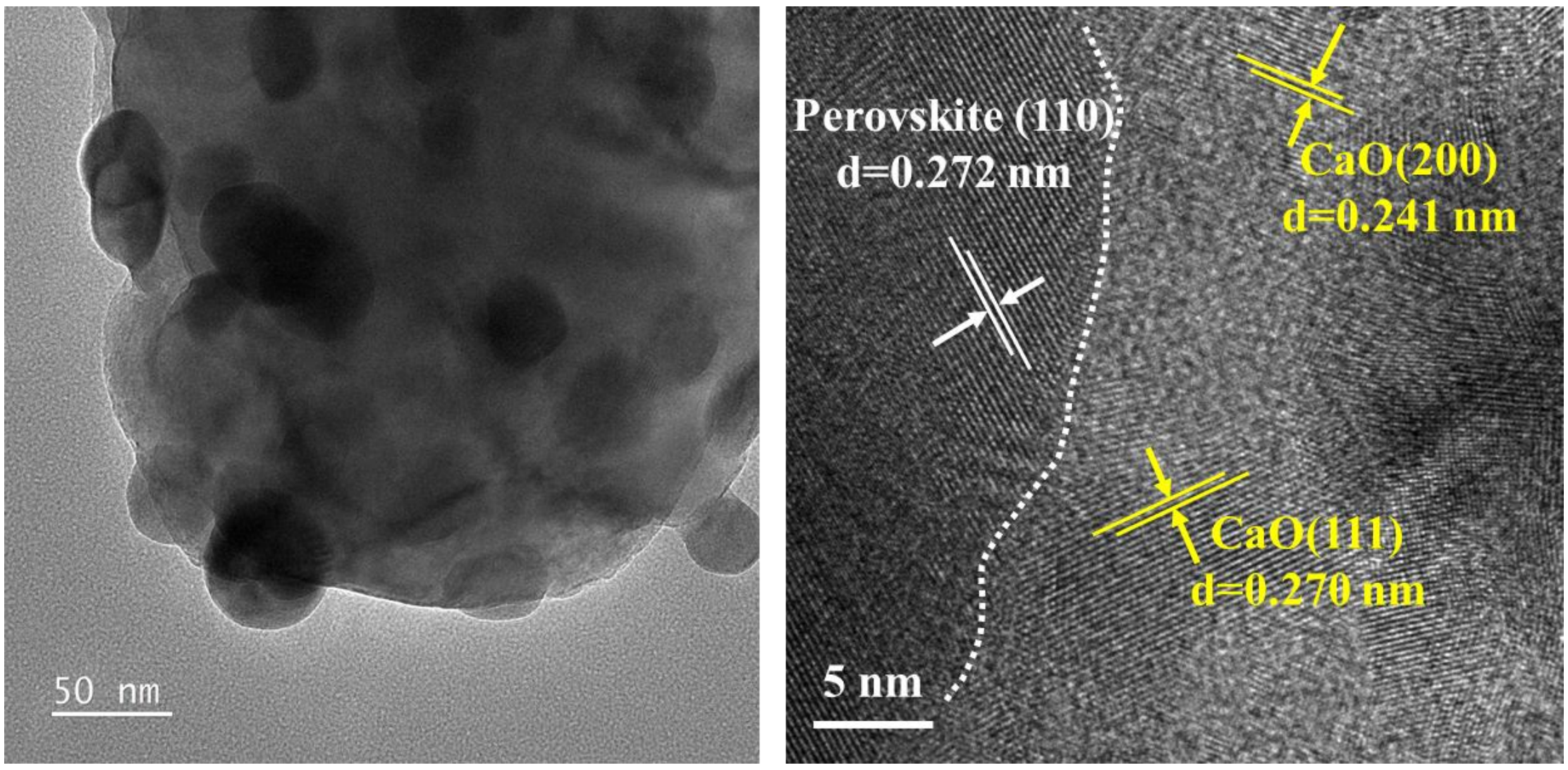

Supplementary Fig. 21 Morphological characterizations. HRTEM images of as-synthesized $LaNi_{0.8}Al_{0.2}O_3$/CaO DFM with intimately contacted heterogeneous interfaces.

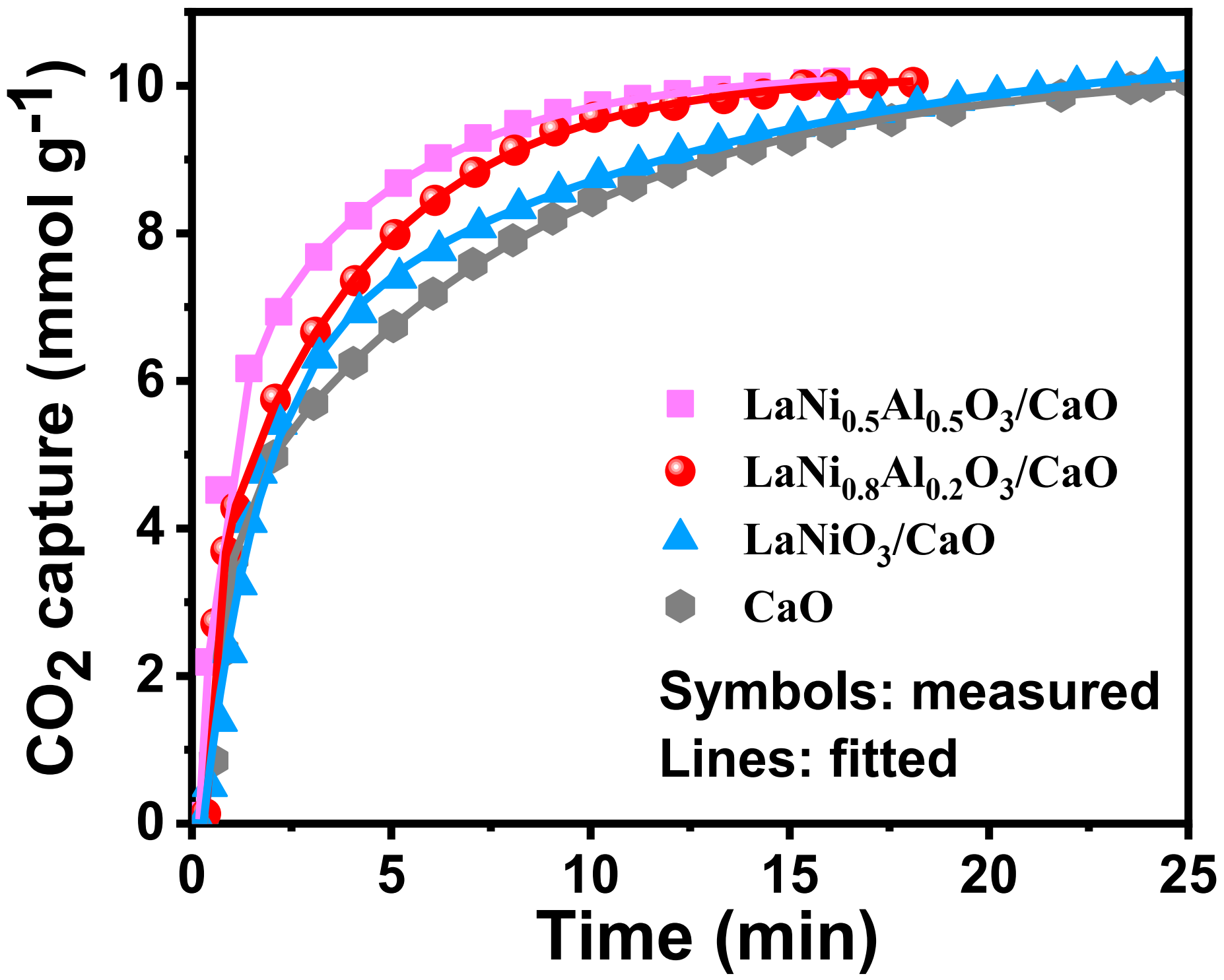

Supplementary Fig. 22 Adsorption kinetics analysis. Experimental and kinetic fittings of the CO_2 adsorption on fresh $LaNi_{1-x}Al_xO_3$/CaO DFMs (Test conditions: 650 °C, 10vol% CO_2 /2vol%O_2 /N_2)

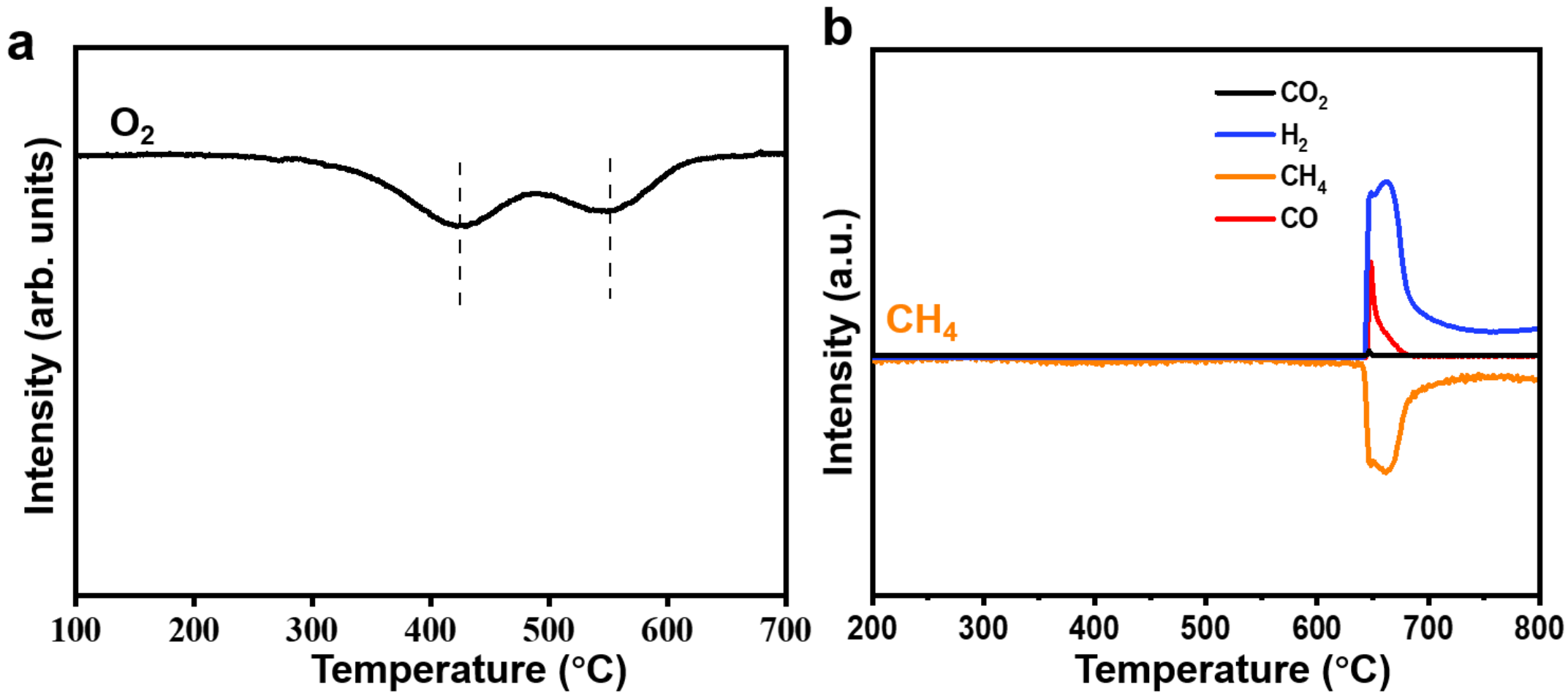

Supplementary Fig. 23 Temperature-programmed surface reaction performance. (a) O_2 -temperature-programmed oxidation (O_2-TPO) profile of the reduced $LaNi_{0.8}Al_{0.2}O_3$/CaO. (b) CH_4-temperature-programmed surface reactions (TPSR) profile of the oxidative $LaNi_{0.8}Al_{0.2}O_3$/CaO.

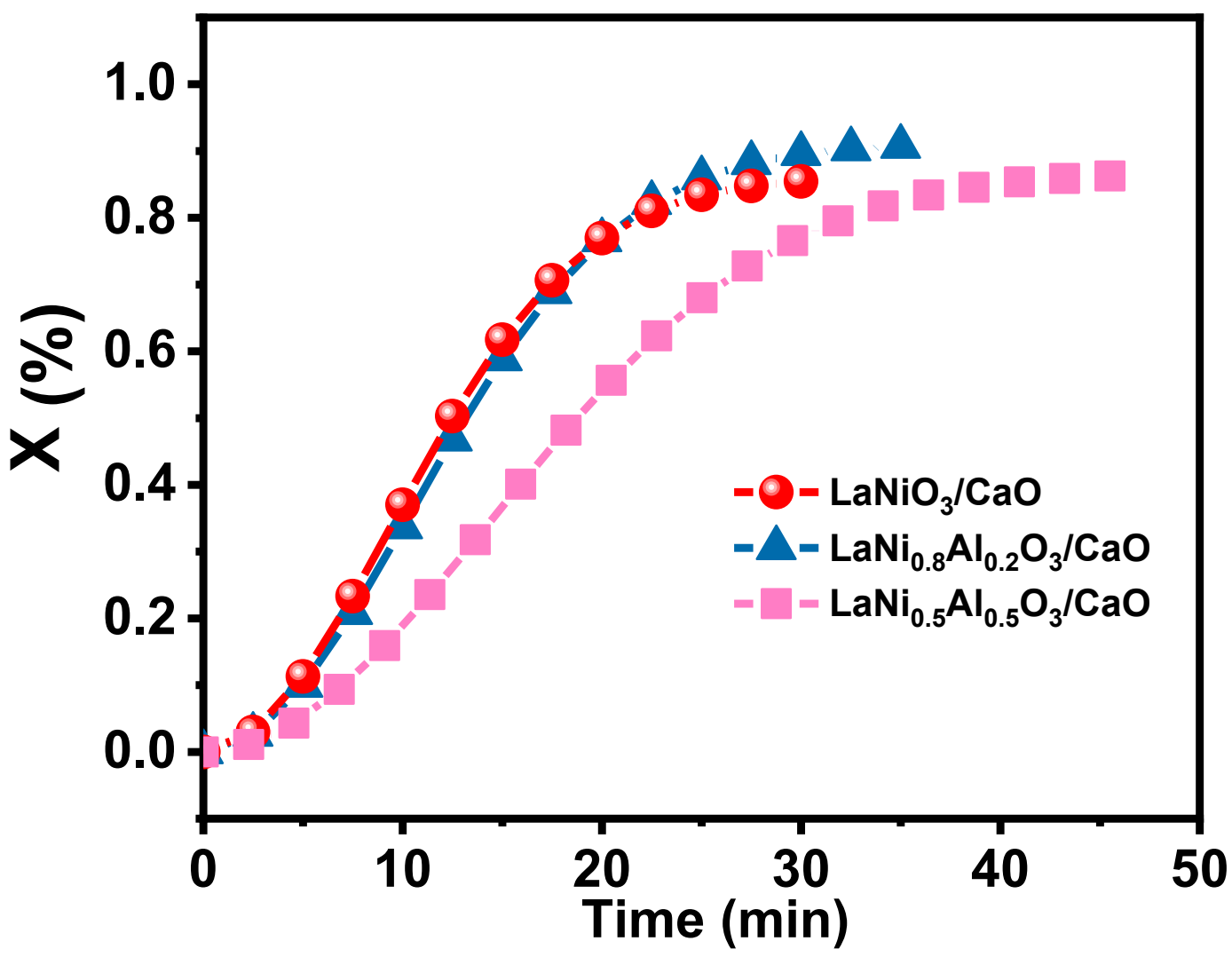

Supplementary Fig. 24 The kinetic of in-situ conversion after CO_2 capture. The conversion curves of adsorbed CO_2 (X) with the reaction time.

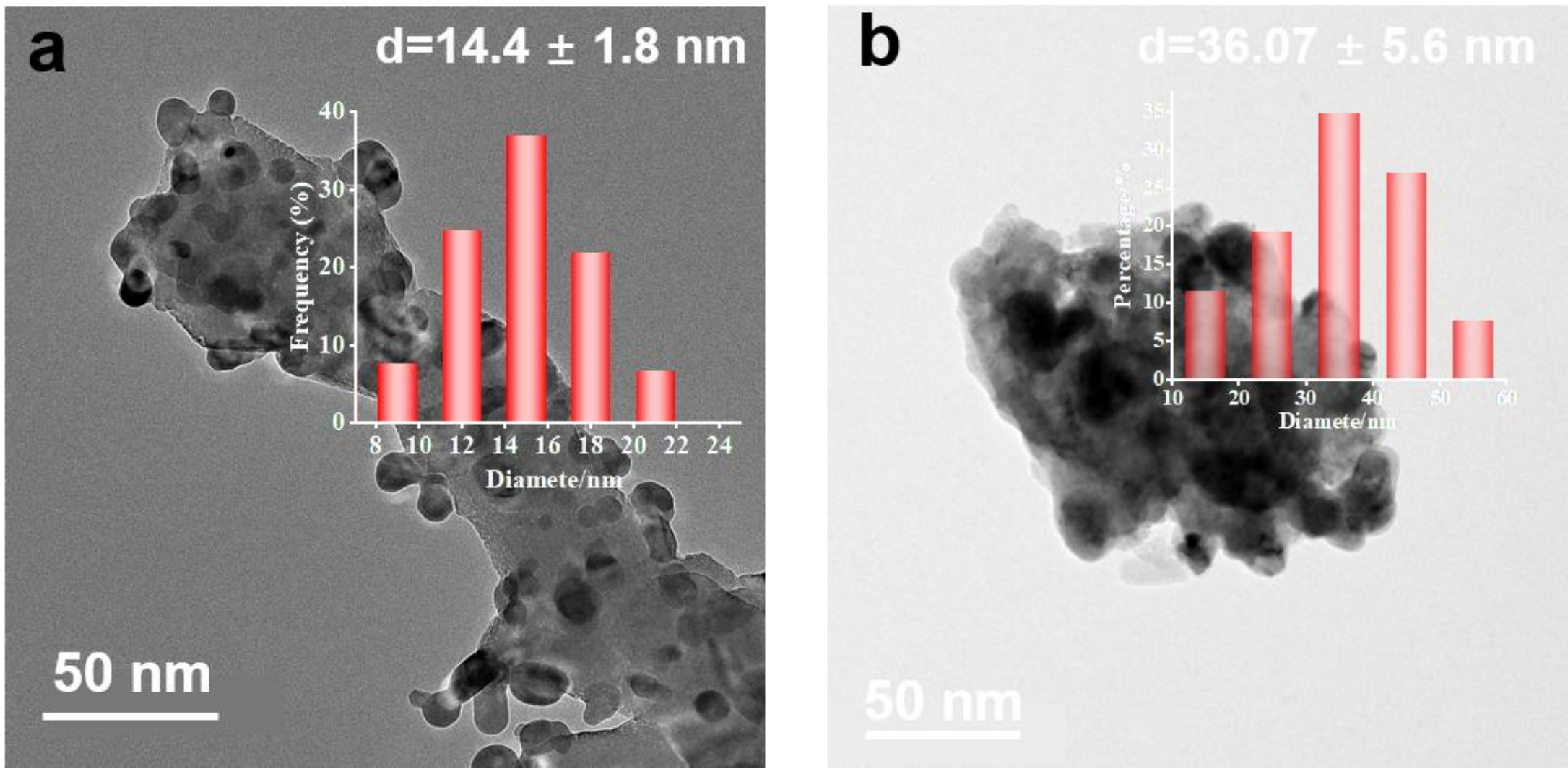

Supplementary Fig. 25 Morphological characterizations. HRTEM images of (a) fresh and (b) spent $LaNiO_3/CaO$ DFM after 25 cyclic iCCC-DRM process.

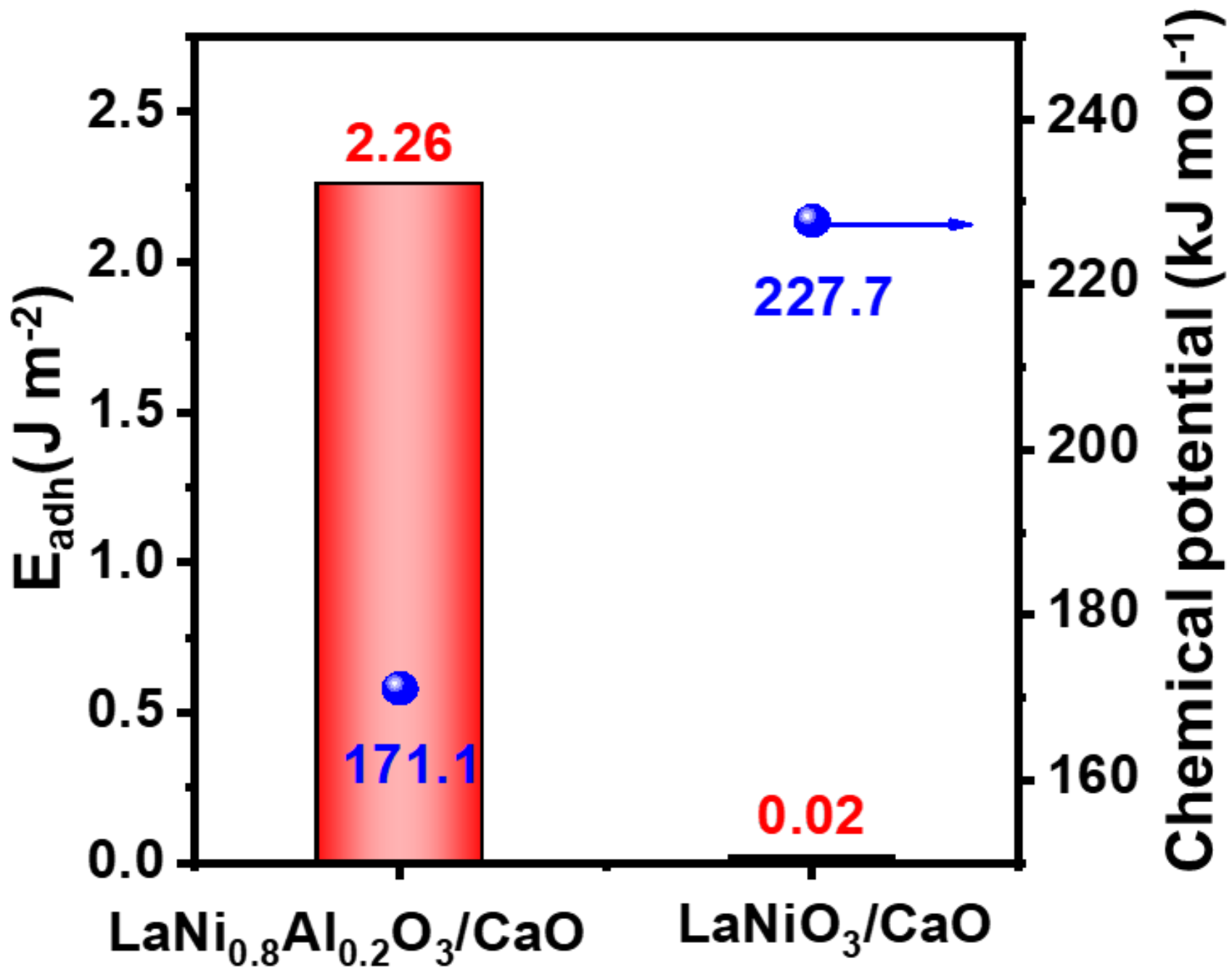

Supplementary Fig. 26 Exploration of in-between adhesion energy. The adhesion energy (E_{adh}) and chemical potential (μ) of (G) $LaNi_{0.8}Al_{0.2}O_3$/CaO and (H) $LaNiO_3$/CaO DFMs.

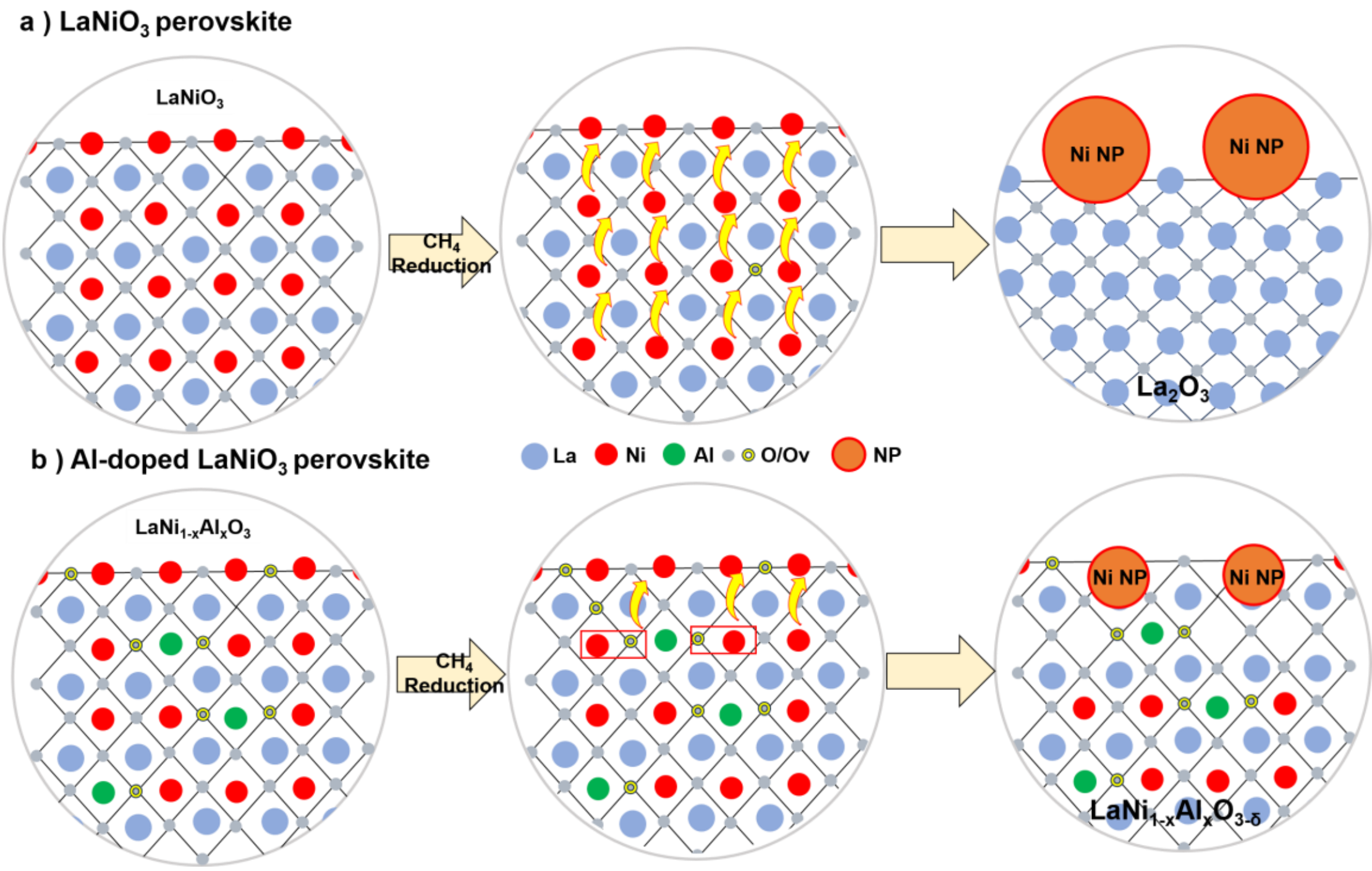

Supplementary Fig. 27 Schematic illustration of the Ni exsolution process. (a) The Ni exsolution of $LaNiO_3$/CaO and (b) Al-doped $LaNiO_3$/CaO under reductive CH_4 atmosphere (CaO/$CaCO_3$ transformation is omitted for clarity).

Supplementary Table 1 Quasi-in situ high-resolution O1S XPS measurements of $LaNi_{0.8}Al_{0.2}O_3/CaO$ DFM.

Stage	O_L	O_V	O_{OH}	O_V/O_L
H_2 reduction	6.06%	40.20%	53.74	6.6
CO_2 capture	22.50%	32.17%	45.33%	1.4
CH_4 reduction	6.35%	43.39%	50.26%	6.8

Supplementary Table 2 quasi-in situ high-resolution Ni 3p XPS measurements of $LaNi_{0.8}Al_{0.2}O_3$-CaO DFM during iCCC-DRM reaction.

Stage	Ni^0	Ni^{2+}	Ni^{3+}
H_2 reduction	34.50%	36.50%	29.00%
CO_2 capture	0	67.40%	32.60%
CH_4 reduction	45.85%	17.70%	36.40%

Supplementary Table 3 Elemental analysis, average crystallite size, and textural properties of fresh $LaNi_{1-x}Al_xO_3$-CaO and Ni/CaO

Samples	La (wt%)	Ni (wt%)	Al (wt%)	La/Ni/Al molar ratio	S_{BET} (m^2/g)	D_{CaO} (nm)
CaO	-	-	-	-	18.8	55.6
Ni/CaO	-	5.01	-	-	13.7	48.5
$LaNi_{0.8}Al_{0.2}O_3$	57.8	21.70	2.35	1/0.80/0.20	11.3	-
$LaNiO_3$/CaO	14.96	6.57	-	1/1.04	7.9	29.8
$LaNi_{0.9}Al_{0.1}O_3$/CaO	14.98	5.80	0.31	1/0.92/0.11	8.9	29.6
$LaNi_{0.8}Al_{0.2}O_3$/CaO	14.78	4.98	0.59	1/0.80/0.21	17.6	28.7
$LaNi_{0.7}Al_{0.3}O_3$/CaO	14.56	3.67	0.75	1/0.76/0.24	15.1	27.3
$LaNi_{0.5}Al_{0.5}O_3$/CaO	14.42	3.21	1.26	1/0.53/0.45	6.1	26.5
$LaNi_{0.8}Al_{0.2}O_3$/CaO-PM	13.34	5.01	0.54	1/0.80/0.20	14.7	55.6

Supplementary Table 4 Lattice structure and refined lattice parameters of fresh $LaNi_{1-x}Al_xO_3$/CaO perovskite in CaO samples.

x	Lattice structure	Space group	a, b (Å)	c (Å)	Vol ($Å^3$)	t	μ
0	Trigonal	$R\bar{3}c$	5.451	6.564	168.900	0.976	0.400
0.1	Trigonal	$R\bar{3}c$	5.440	6.553	168.810	0.979	0.398
0.2	Trigonal	$R\bar{3}c$	5.430	6.541	168.610	0.982	0.396
0.3	Trigonal	$R\bar{3}c$	5.418	6.512	168.450	0.985	0.395
0.5	Trigonal	$R\bar{3}c$	5.403	6.498	168.217	0.992	0.391

Supplementary Table 5 The numerical comparison of iCCC-DRM processes and reported conventional DRM process in the literature.

Catalyst	Gas composition	WHSV	Temperature (℃)	CH_4 conversion (%)	CO_2 conversion (%)	H_2/CO molar ratio ($R_{H/C}$)	Ref.
$LaNi_{0.8}Al_{0.2}O_3$/CaO	**Capture step:** 10 vol.% CO_2/N_2; **Conversion step:** 5vol.% CH_4/N_2	30 L g^{-1} h^{-1} (50 ml/min)	650	93.5	91.5	1.05	**This work**
$Ca_2Ni_{0.05}Ti_{0.95}$	**Capture step:** 10 vol.% CO_2/N_2; **Conversion step:** 5vol.% CH_4/N_2	60 L g^{-1} h^{-1} (50 mL/min)	700	/	/	0.73	1
$Ce_{0.75}Co_{0.25}NiO_3$/Ca	**Capture step:** 10 vol.% CO_2/Ar; **Conversion step:** 5vol.% CH_4/Ar	3 L g^{-1} h^{-1} (25 mL/min)	700	94.0	77.0	2.30	2
5Ni-30CaO-$LaFeO_3$	**Capture step:** 15vol.% CO_2/Ar; **Conversion step:** 5vol.% CH_4/Ar	10 L g^{-1} h^{-1} (50 mL/min)	700	74.8	86.5	1.0	3
Ni/CaO-Al_2O_3	**Capture step:** 15vol.% CO_2/N_2; **Conversion step:** 3vol.% CH_4/N_2	60 L g^{-1} h^{-1} (100mL/min)	650	74.7	83.6	0.96	4
Ni-CaO catal-sorbent	**Capture step:** 10 vol.% CO_2/N_2; **Conversion step:** 10vol.% CH_4/N_2	12 L g^{-1} h^{-1} (40 ml/min)	700	/	83.8	6.53	5
Ni-Ca@Zr	**Capture step:** 5 vol.% CO_2/N_2; **Conversion step:** 8 vol.% CH_4/N_2	36 L g^{-1} h^{-1} (30 ml/min)	720	40	45	0.8	6
Ni/NiCa (2.6 wt%)	**Capture step:** 10% CO2/Ar, **Conversion step:** 10%CH4/Ar,	60 L g^{-1} h^{-1} (20 mL/min)	650	67	78	0.9	7
Ni-Ca_{10}Mg	**Capture step:** 20% CO_2/N_2 **Conversion step:** 10%CH_4/N_2,	24 L g^{-1} h^{-1} (200 mL/min)	720	90	60	0.92	8
$NiCa_{1.6}K_{0.4}$@Al_2O_3	**Capture step:** 20 % CO_2/Ar **Conversion step:** 10%CH4/Ar,	1.5 L g^{-1} h^{-1} (50ml/min)	750	25	52	2.1	9

Ni/SiO_2-E	$CH_4/CO_2/N_2$ = 3:3:4	30 L g^{-1} h^{-1}	500	7.9	17.9	0.5	10
0.2Ce-$La_{0.97}Ni_{0.4}Cr_{0.6}O_3$	CH_4: CO_2: N_2 = 1: 1: 1	30 L g^{-1} h^{-1}	800	87.4	92.9	0.93	11
10NiOMgO-PACS	CH_4/CO_2/He=1:1:3	72 L g^{-1} h^{-1}	700	78.5	93.7	0.85	12
NiFe/Al_2O_3	CH_4/CO_2 = 1:1	12 L g^{-1} h^{-1}	550	26.6	37.8	0.67	13
$MgNiO_2$@Ni@$MgNiO_2$	CH_4: CO_2 = 1:1	70 L g^{-1} h^{-1}	700	68	78	0.9	14
Ni-CaO/MCM-41	CH_4/CO_2/Ar = 1:1:1	143 L g^{-1} h^{-1}	650	20	40	0.5	15
15%Ni@S-1	CH_4/CO_2 = 1:1	100 L g^{-1} h^{-1}	650	40	42	0.6	16
$Ni_3Fe_1Cu_1$-MA	$CH_4/CO_2/N_2$ = 2:2:1	432 L g^{-1} h^{-1}	650	15	35	0.5	17
Ni/MCF	CH_4/CO_2/Ar = 9:9:2	36 L g^{-1} h^{-1}	650	60	70	1.0	18
Ni/MgO	CH_4/CO_2/Ar = 1:1:8	30 L g^{-1} h^{-1}	700	⩾37	⩾50	0.78	19
0.8MgO^{WI}/Ni@NiAlO	CH_4/CO_2/Ar=1:1:3	60 L g^{-1} h^{-1}	600	50	61	/	20
NiMo/MgO	CH_4/CO_2/He = 1:1:8	60 L·g^{-1} h^{-1}	800	100	100	1	21

Supplementary Table 6 Kinetic parameters of CO_2 adsorption on CaO sorbent and Al-doped $LaNiO_3$/CaO DFMs at 650°C.

Samples	A_1	A_2	A_3	k_1 / min^{-1}	k_2 / min^{-1}	R^2
CaO	10.68	-7.36	-4.78	0.72	0.09	0.997
$LaNiO_3$/CaO	10.27	-7.13	-6.77	2.14	0.13	0.999
$LaNi_{0.8}Al_{0.2}O_3$/CaO	10.15	-12.72	-7.40	3.90	0.22	0.999
$LaNi_{0.5}Al_{0.5}O_3$/CaO	10.17	-13.28	-6.89	4.17	0.25	0.999

Supplementary References

1. Jo, S. & Gilliard-AbdulAziz, K. L. Self-Regenerative Ni-Doped $CaTiO_3$/CaO for Integrated CO_2 Capture and Dry Reforming of Methane. *Small* e2401156 (2024).
2. Baamran, K., Lawson, S., Rownaghi, A. A. & Rezaei, F. Reactive Capture and Conversion of CO_2 into Hydrogen over Bifunctional Structured $Ce_{1-x}Co_xNiO_3$/Ca Perovskite-Type Oxide Monoliths. *JACS. Au.* **4**, 101–115 (2023).
3. Wei, H. *et al.* Carbon-resistant bifunctional catalyst composed of $LaFeO_3$ enhanced Ni-CaO for integrated CO_2 capture and conversion. *Carbon Capt. Sci. Technol.* 100358–100358 (2025).
4. Zhang, L., Zhou, Y., Cui, H., Cheng, Z. & Zhou, Z. Performance enhancement of Ni/CaO-Al_2O_3 dual-function materials for integrated CO_2 capture and conversion via dry reforming of methane. *Chem. Eng. Sci.* **320**, 122635 (2026).
5. Jo, S. B. *et al.* CO_2 green technologies in CO_2 capture and direct utilization processes: methanation, reverse water-gas shift, and dry reforming of methane. *Sustainable Energy Fuels* **4**, 5543–5549 (2020).
6. Hu, J., Hongmanorom, P., Galvita, V. V., Li, Z. & Kawi, S. Bifunctional Ni-Ca based material for integrated CO_2 capture and conversion via calcium-looping dry reforming. *Appl. Catal. B Environ.* **284**, 119734 (2021).
7. Hu, J. *et al.* Tandem distributing Ni into CaO framework for isothermal integration of CO_2 capture and conversion. *Chem. Eng. J.* **452**, 139460 (2023).
8. Han, R. *et al.* Two birds with one stone: MgO promoted Ni-CaO as stable and coke-resistant bifunctional materials for integrated CO_2 capture and conversion. *Separation Purif. Technol.* **307**, 122808 (2023).
9. Jiang, R., Miao, Z. & Shen, L. Study on K-modified Ca-based dual-functional materials for carbon capture and in-situ methane dry reforming. *J. Energy Inst.* **117**, 101847 (2024).
10. Wang, F., Han, K., Xu, L., Yu, H. & Shi, W. Ni/SiO_2 Catalyst Prepared by Strong Electrostatic Adsorption for a Low-Temperature Methane Dry Reforming Reaction. *Ind. Eng. Chem. Res.* **60**, 3324–3333 (2021).
11. Hao, C. *et al.* Ce-induced synergistic effect in exsolved perovskite catalyst for highly efficient and robust methane dry reforming. *Nat Commun* **16**, 10630 (2025).
12. Danghyan, V., Kumar, A., Mukasyan, A. & Wolf, E. E. An active and stable NiOMgO solid solution based catalysts prepared by paper assisted combustion synthesis for the dry reforming of methane. *Appl. Catal. B Environ.* **273**, 119056 (2020).
13. Song, Z. *et al.* Improved Effect of Fe on the Stable NiFe/Al_2O_3 Catalyst in Low-Temperature Dry Reforming of Methane. *Ind. Eng. Chem. Res.* **59**, 17250–17258 (2020).
14. Wang, Q. *et al.* Electron-deficient Ni0 sites stabilized by dual Ni/$MgNiO_2$ interface enable efficient and durable dry reforming of methane. *Appl. Catal. B Environ.* **383**, 126105 (2026).
15. Wu, P. *et al.* Cooperation of Ni and CaO at Interface for CO_2 Reforming of CH_4: A Combined Theoretical and Experimental Study. *ACS Catal.* **9**, 10060–10069 (2019).
16. Liu, Y. *et al.* Embedding high loading and uniform Ni nanoparticles into silicalite-1 zeolite for dry reforming of methane. *Appl. Catal. B Environ.* **307**, 121202 (2022).
17. Jin, F. *et al.* Stable Trimetallic NiFeCu Catalysts with High Carbon Resistance for Dry Reforming of Methane. *ChemPlusChem* **85**, 1120–1128 (2020).

18. Daoura, O. *et al.* Mesocellular silica foam-based Ni catalysts for dry reforming of CH_4 (by CO_2). *J. CO_2. Util.* **24**, 112–119 (2018).
19. Cheng, F., Duan, X. & Xie, K. Dry Reforming of CH_4/$CO_2$by Stable Ni Nanocrystals on Porous Single-Crystalline MgO Monoliths at Reduced Temperature. *Angew Chem Int Ed* **60**, 18792–18799 (2021).
20. Wang, Q. *et al.* Interface engineering of oxygen-vacancy-rich MgO/Ni@NiAlO enables low-temperature coke-free methane dry reforming. *Chin. J. Catal.* **75**, 9–20 (2025).
21. Song, Y. *et al.* Dry reforming of methane by stable Ni–Mo nanocatalysts on single-crystalline MgO. *Science* **367**, 777–781 (2020).
