# Supplementary material for: Self-adaptive Ni nanoparticles in perovskite LaNi1-xAlxO3/CaO for durable CO2 capture and in-situ conversion
Source: Nat Commun. 2026 Jul 21;17:8903. doi: 10.1038/s41467-026-75734-x (PMC13503938; doi:10.1038/s41467-026-75734-x)
Supplement: Supplementary file 2 [file 41467_2026_75734_MOESM2_ESM.pdf]

nature portfolio

Peer Review File

Self-Adaptive Ni Nanoparticles in Perovskite LaNi1-xAlxO3/CaO for Durable CO2 Capture and in-situ Conversion

Corresponding Author: Professor Jun Hu

This file contains all reviewer reports in order by version, followed by all author rebuttals in order by version.

Version 0:

Reviewer comments:

Reviewer #1

(Remarks to the Author)
The authors present a noteworthy attempt to integrate exsolution-based catalyst design with a CaO-based CO_2 capture and conversion system, which remains largely underexplored. The authors propose a dynamically adaptive catalyst system in which reversible exsolution–dissolution of Ni nanoparticles is inherently coupled with the cyclic nature of CO_2 capture and conversion. Furthermore, the use of Al doping to optimize performance and durability of this materials system provides a rational materials design strategy that connects lattice chemistry with catalytic functionality. However, However, for this study to go beyond a simple combination of existing concepts and to establish itself as a meaningful extension of exsolution catalysts into a new application area, the following issues should be carefully addressed. I believe that the manuscript can only be considered for publication after the following issues are thoroughly addressed by the authors.

1) A direct comparison with a conventional Ni/CaO catalyst system under identical conditions would significantly clarify the advantages of the proposed catalyst. It is strongly recommended to benchmark the performance against a commercially relevant and widely used system. In this comparison, the Ni loading should be comparable to the total Ni content present in the exsolution-based catalyst to ensure a fair evaluation.

2) In the iCCC process, CO_2 capture is primarily governed by CaO. Although the authors attribute enhanced CO_2 adsorption kinetics to the increased oxygen vacancy (Ov) concentration in Al-doped $LaNiO_3$, this effect is inherently limited to the perovskite phase. Therefore, it remains unclear how the Ov in $La(Ni,Al)O_3$ significantly influences the overall CO_2 capture behavior, which is expected to be dominated by CaO. Furthermore, I think Figure 5A does not sufficiently support the formation of O^{2-} channels as claimed. The authors should more clearly elucidate how oxygen vacancies in $La(Ni,Al)O_3$ contribute to the adsorption mechanism. Revision of the figure may also be necessary to better support this claim.

3. Following the above point, a control experiment using a simple physical mixture of Al-doped $LaNiO_3$ and CaO would be highly beneficial. Such a comparison would help clarify whether the observed performance enhancement originates from the proposed one-pot synthesis and structural integration, rather than from a simple combination of the two components.

4. If possible, the catalytic performance should be further evaluated in terms of intrinsic activity metrics, such as mass activity or turnover frequency (TOF), rather than relying solely on conversion values. This would allow a more rigorous assessment of the catalytic efficiency.

5. A major concern associated with the one-pot synthesis approach is the potential incorporation of Ca into the Al-doped $LaNiO_3$ lattice, which may obscure the distinction between the two phases. While the crystal structures of Al-doped $LaNiO_3$ and CaO are distinguishable, the results shown in Figure S2 and Figure 2D suggest that Ca incorporation into the perovskite phase cannot be ruled out. If such incorporation occurs, particularly in a manner dependent on the Al doping level, it could significantly affect the interpretation of the results and the proposed mechanism. Therefore, the authors should provide more rigorous analytical evidence to clearly confirm the phase separation and exclude possible Ca substitution in the perovskite lattice.

Reviewer #2

(Remarks to the Author)
The manuscript presents an interesting concept of a self-adaptive Ni-containing catalyst/adsorbent system for coupled CO_2 capture and methane conversion. The topic is timely and potentially relevant to Nature Communications. However, the novelty claim appears overstated, and the mechanistic interpretation is not yet sufficiently convincing. The authors claim that this type of exsolution-enhanced/sorbent system has not been previously reported. This statement is incorrect. Closely related systems combining Ni-containing perovskites, such as $LaFeNiO_3$–CaO (Small, 10.1002/smll.202401156), have been reported in the literature. The failure to cite and discuss this and similar studies is a serious omission. At a minimum, the authors must explicitly compare their material and performance against these prior reports (as partially done for Ni-based catalysts in Table S6). Without such benchmarking, the manuscript does not meet the standard of novelty expected for this journal.

Major points
1. The manuscript states that the material/system has not been reported before, but the literature contains closely related examples that should be cited and compared directly. The absence of this discussion weakens the novelty claim and gives the impression that the authors did not sufficiently benchmark the work against prior studies.
2. The proposed theory of oxygen-vacancy diffusion from the perovskite phase to CaO is not convincing as presently written. This mechanism needs stronger experimental or computational support, or the authors should revise the interpretation to a more conservative and defensible explanation.
3. The manuscript would benefit from additional imaging and interfacial characterization to clarify how the perovskite is connected to the CaO grains. Larger-scale STEM-EDS imaging would help establish whether the interface architecture is truly responsible for the observed cycling behavior.
4. The conclusion that Ni is reversibly exsolving and redistributing because of $LaAlO_3$ is not well supported. Since exsolution is usually associated with reducible B-site cations, the rationale for Al^{3+}-driven reversible exsolution is not persuasive as currently presented. A more cautious interpretation may be that Ni is remaining more stably dispersed near the surface during cycling, rather than undergoing fully reversible exsolution/redistribution.
5. Additional characterization after 50 cycles or after aging is critical for linking the seemingly stable performance to the structure-function properties of the perovskite oxide catalyst coupled to CaO.

Minor point
• Page 6: replace “dopped” with “doped”.

Version 1:

Reviewer comments:

Reviewer #1

(Remarks to the Author)
The authors have successfully addressed all of the concerns raised during the previous round of review. In particular, they performed the additional experiments that were suggested and provided direct comparisons with conventional catalyst systems, substantially strengthening the evidence supporting their claims. The responses to the reviewers' questions were accompanied by appropriate references and well-reasoned explanations, which I found convincing.

With the inclusion of these additional datasets and analyses, I believe that the scientific rigor and completeness of the study have been significantly improved. The manuscript now meets the standard expected for publication in a high-profile Nature Communications journal. Therefore, I recommend acceptance of the manuscript in its current form.

Reviewer #2

(Remarks to the Author)
The manuscript was acceptable for publication. The authors addressed all of my concerns.

Open Access This Peer Review File is licensed under a Creative Commons Attribution 4.0 International License, which permits use, sharing, adaptation, distribution and reproduction in any medium or format, as long as you give appropriate credit to the original author(s) and the source, provide a link to the Creative Commons license, and indicate if changes were made.
In cases where reviewers are anonymous, credit should be given to 'Anonymous Referee' and the source.
The images or other third party material in this Peer Review File are included in the article's Creative Commons license, unless indicated otherwise in a credit line to the material. If material is not included in the article's Creative Commons license and your intended use is not permitted by statutory regulation or exceeds the permitted use, you will need to obtain permission directly from the copyright holder.
To view a copy of this license, visit https://creativecommons.org/licenses/by/4.0/

Response to Reviewers

Reviewer #1

"*The authors present a noteworthy attempt to integrate exsolution-based catalyst design with a CaO-based CO_2 capture and conversion system, which remains largely underexplored. The authors propose a dynamically adaptive catalyst system in which reversible exsolution–dissolution of Ni nanoparticles is inherently coupled with the cyclic nature of CO_2 capture and conversion. Furthermore, the use of Al doping to optimize performance and durability of this materials system provides a rational materials design strategy that connects lattice chemistry with catalytic functionality. However, for this study to go beyond a simple combination of existing concepts and to establish itself as a meaningful extension of exsolution catalysts into a new application area, the following issues should be carefully addressed. I believe that the manuscript can only be considered for publication after the following issues are thoroughly addressed by the authors.*"

Response: We sincerely thank the reviewer for recognizing the novelty and significance of this study. We also deeply appreciate the reviewer 's encouraging comments and insightful suggestions, which are valuable and helpful for us to think deeply and hence to improve our manuscript. We have carefully made responses and modifications based on five questions point-by-point as below.

Question 1: "*A direct comparison with a conventional Ni/CaO catalyst system under identical conditions would significantly clarify the advantages of the proposed catalyst. It is strongly recommended to benchmark the performance against a commercially relevant and widely used system. In this comparison, the Ni loading should be comparable to the total Ni content present in the exsolution-based catalyst to ensure a fair evaluation.*"

Response: We thank the reviewer for this valuable suggestion. For comparison, we synthesized the conventional Ni/CaO catalyst using the impregnation method without introducing La and Al. The Ni loading was about 5 wt%, which was comparable to the total Ni content present in the optimized $LaNi_{0.8}Al_{0.2}O_3$/CaO (**Table S3**). The XRD pattern of as-synthesized Ni/CaO DFM shows characteristic CaO peaks (JCPD 44-1481) and broad peaks of NiO (JCPD 47-1049) without any $CaNiO_3$ perovskite or CaNi alloy phases (**Figure R1**). After reduction in H_2 atmosphere at 700 °C, NiO was converted

into Ni metal (JCPD 04-0850). HAADF-STEM-EDS images illustrated the reduced Ni nanoparticles (NPs) were dispersed in CaO matrix, with average size of about 28.6 nm (**Figure R2**). In comparison, HAADF-STEM image of the optimized DFM of $LaNi_{0.8}Al_{0.2}O_3/CaO$ shows that $LaNi_{0.8}Al_{0.2}O_3$ perovskite particles were dispersed in CaO matrix (**Figure R3**), where the EDS mapping images demonstrate the almost overlapped La, Ni, Al elements in $LaNi_{0.8}Al_{0.2}O_3$ perovskites. HRTEM images confirms this dispersion performance (**Figure R4**). From its enlargement, the Ni NP with lattice fringes of 0.203 nm is found partially embedded in the $LaNi_{1-x}Al_xO_3$ perovskite matrix with the (110) lattice of 0.272 nm, indicative of enhanced metal-support interaction. With such confinement effect of $LaNi_{1-x}Al_xO_3$ perovskite, the exsolution of Ni was constricted, resulting in the average diameter of Ni NPs decreased to 14.3 nm. Accordingly, Al-doped $LaNiO_3$ perovskites can rationally regulate Ni NPs growth and inhibit their sintering.

Table S3. Elemental analysis, average crystallite size, and textural properties of fresh $LaNi_{1-x}Al_xO_3$-CaO and Ni/CaO

Samples	La (wt%)	Ni (wt%)	Al (wt%)	La/Ni/Al molar ratio	S_{BET} (m^2/g)	D_{CaO} (nm)
CaO	-	-	-	-	18.8	55.6
Ni/CaO	-	5.01	-	-	13.7	48.5
$LaNi_{0.8}Al_{0.2}O_3$	57.8	21.70	2.35	1/0.80/0.20	11.3	-
$LaNiO_3$/CaO	14.96	6.57	-	1/1.04	7.9	29.8
$LaNi_{0.9}Al_{0.1}O_3$/CaO	14.98	5.80	0.31	1/0.92/0.11	8.9	29.6
$LaNi_{0.8}Al_{0.2}O_3$/CaO	14.78	4.98	0.59	1/0.80/0.21	17.6	28.7
$LaNi_{0.7}Al_{0.3}O_3$/CaO	14.56	3.67	0.75	1/0.76/0.24	15.1	27.3
$LaNi_{0.5}Al_{0.5}O_3$/CaO	14.42	3.21	1.26	1/0.53/0.45	6.1	26.5
$LaNi_{0.8}Al_{0.2}O_3$/CaO-PM	13.34	5.01	0.54	1/0.80/0.20	14.7	55.6

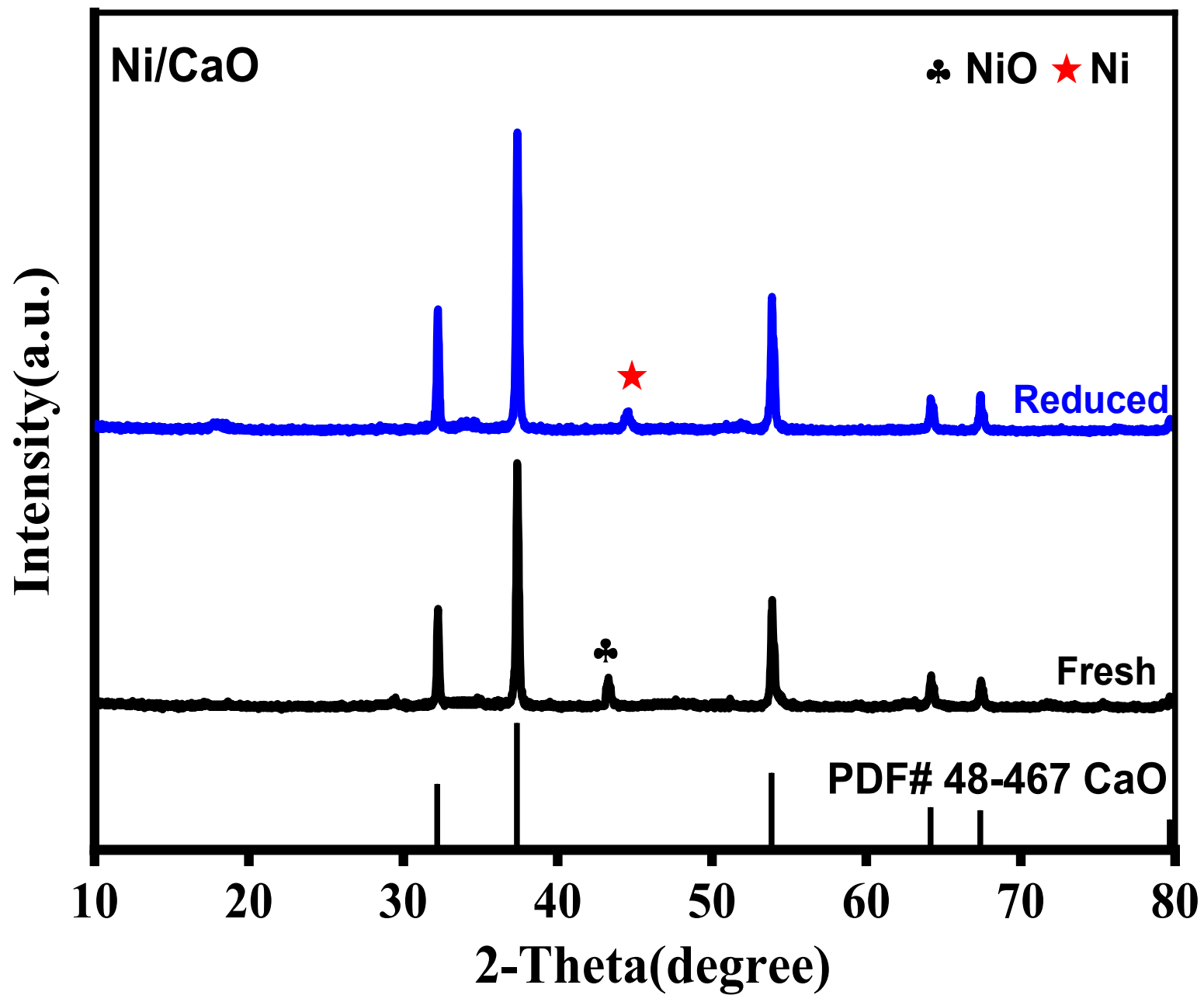

Figure R1(Figure S13a). XRD patterns of as-synthesized and reduced Ni/CaO DFM.

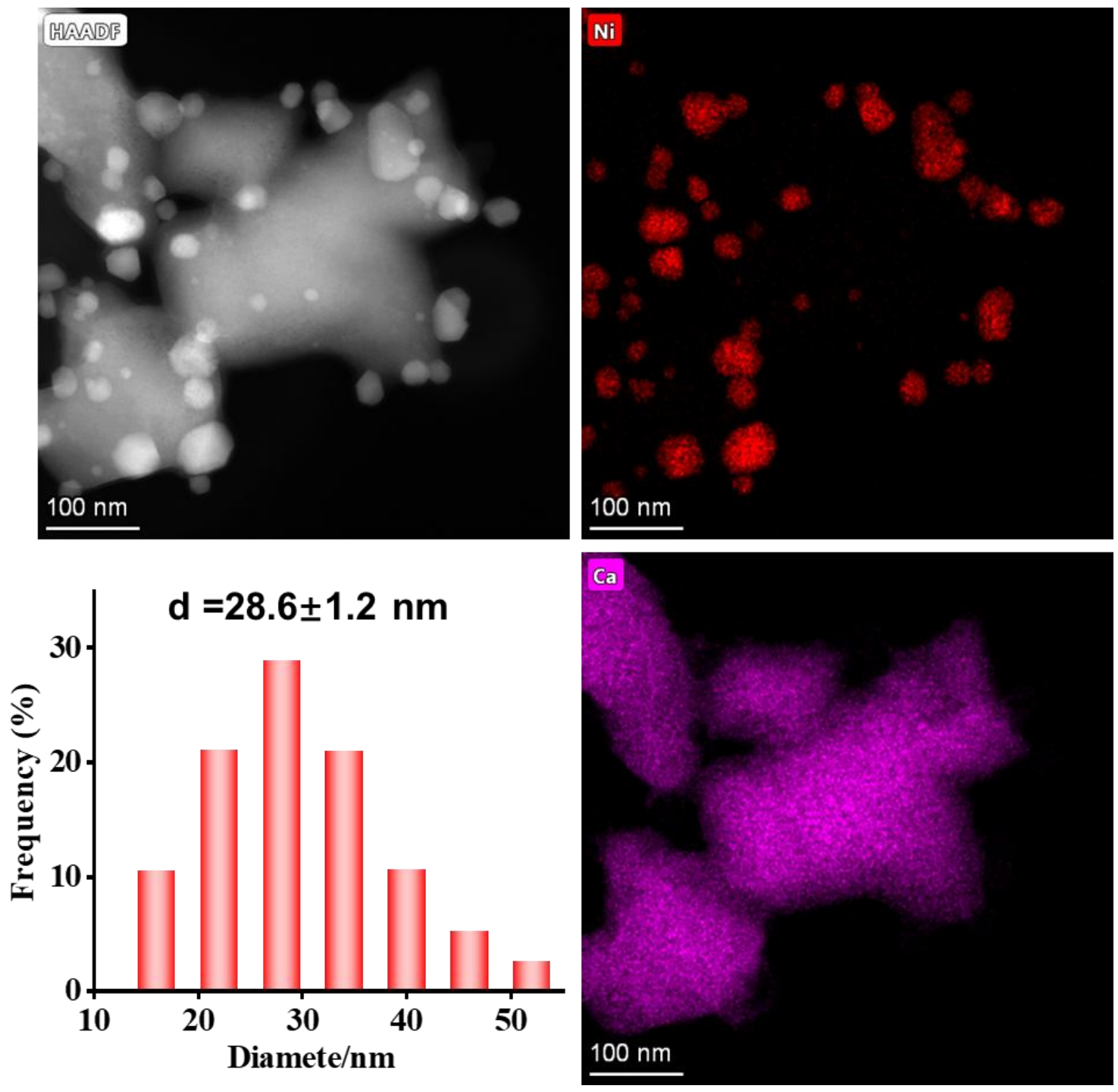

Figure R2 (Figure S13b). HAADF-STEM images and corresponding size distribution of reduced Ni/CaO DFM.

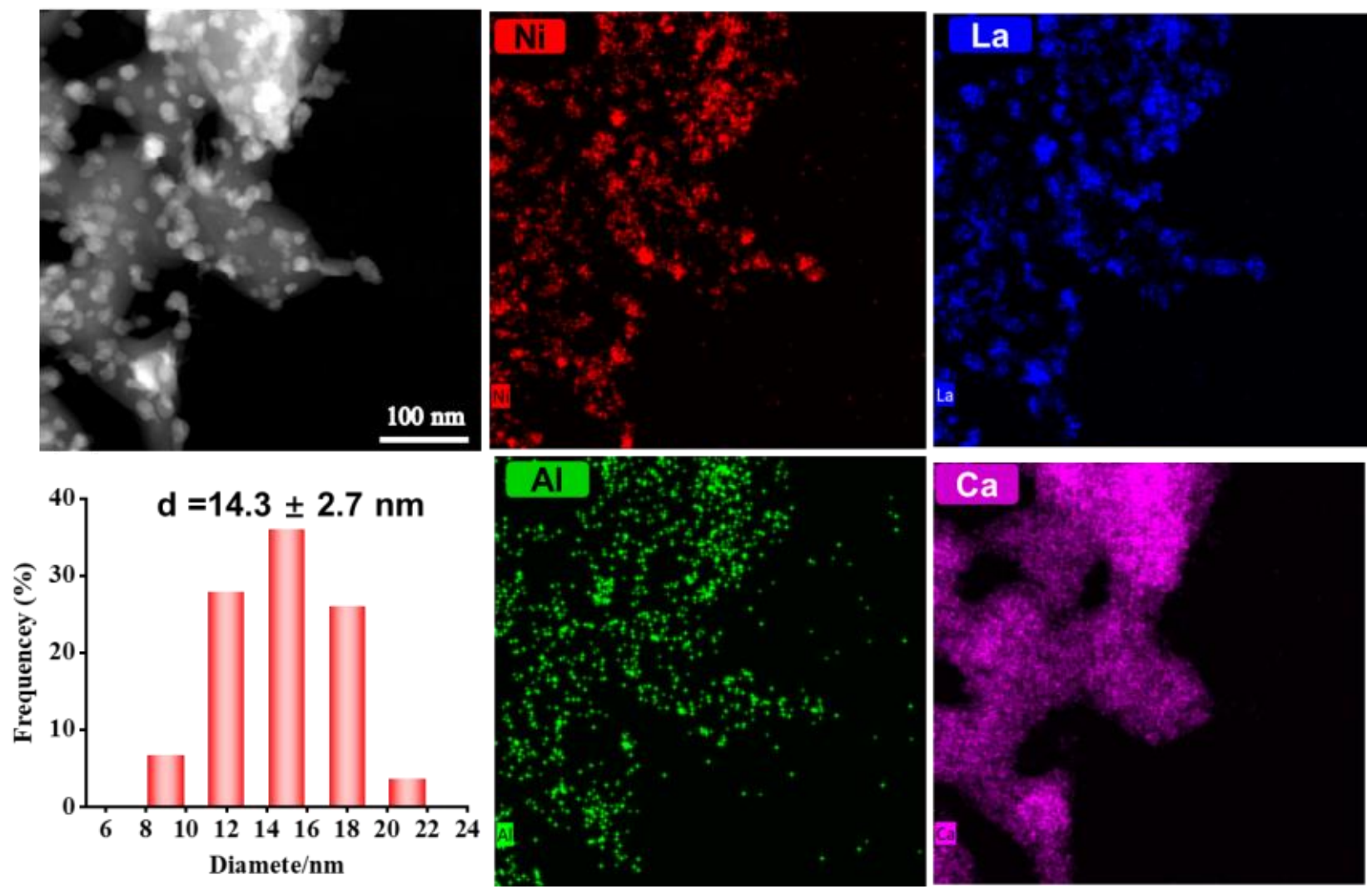

Figure R3 (Figure 6A) HAADF-STEM images and corresponding size distribution of reduced $LaNi_{0.8}Al_{0.2}O_3/CaO$ DFM.

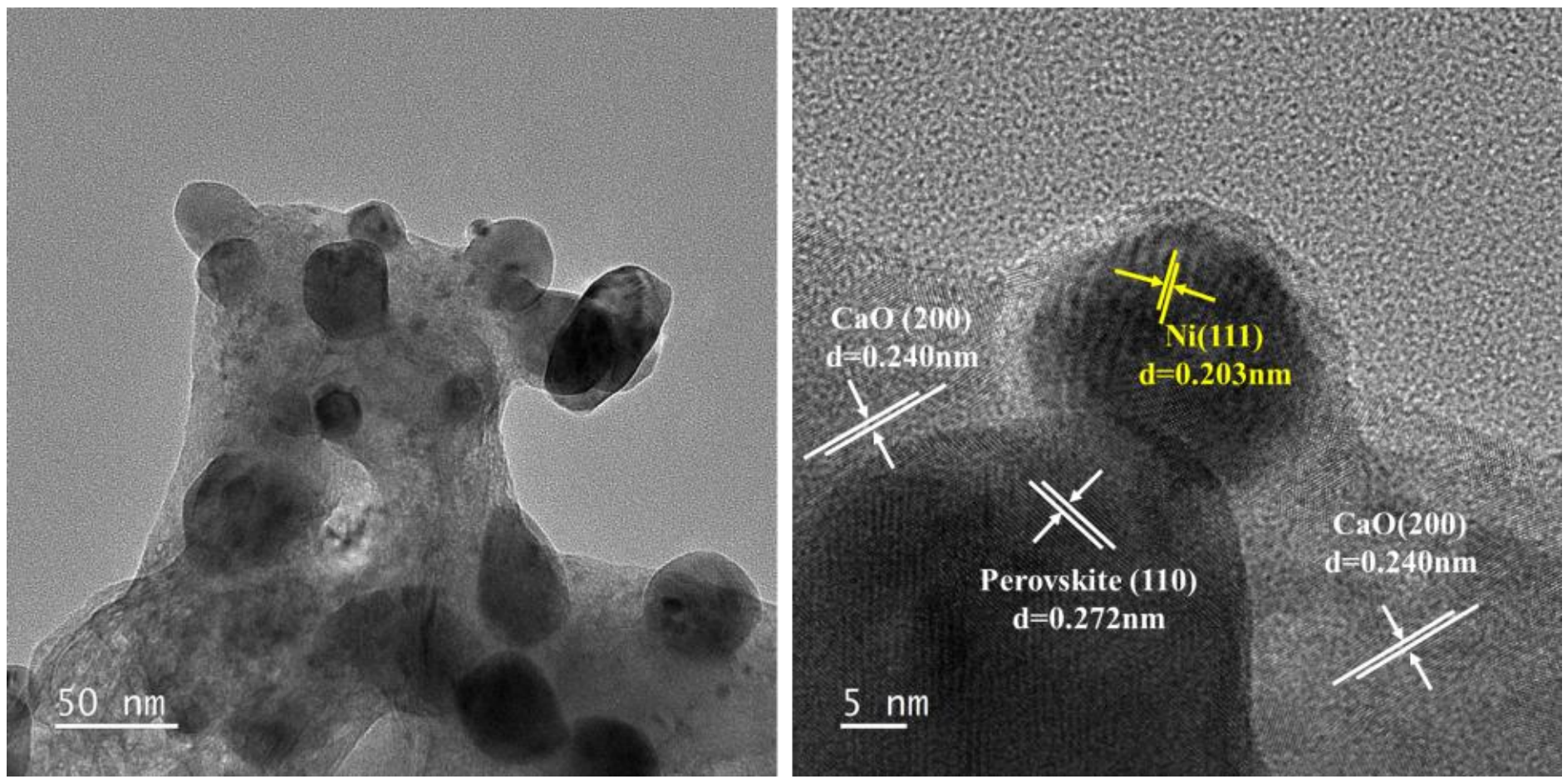

Figure R4 (Figure S5) HRTEM images of the reduced $LaNi_{0.8}Al_{0.2}O_3/CaO$ DFM.

Then, the iCCC-DRM performance of control DFM sample of Ni/CaO was comparative investigated at 650°C (**Figure R5**). At the stage of CO_2 capture from the simulated flue gas (10vol%CO_2 and 2vol%O_2 balanced by N_2), the Ni/CaO DFM shows a little higher CO_2 adsorption capacity of 10.7 mmol g_{DFM}^{-1} than $LaNi_{0.8}Al_{0.2}O_3/CaO$ of 10.2 mmol g_{DFM}^{-1} because of its more CaO content without loading $LaNiO_3$ perovskite. Whereas at the stage of conversion, the $LaNi_{0.8}Al_{0.2}O_3/CaO$ DFM with smaller exsolved Ni NPs delivers superior CH_4 and CO_2 conversion efficiencies of 93.5% and 91.5%, which are notably higher than those of the conventional Ni/CaO (90.8%, 85.0%). Moreover, calculated in terms of per gram of Ni loading, the $LaNi_{0.8}Al_{0.2}O_3/CaO$ exhibits much faster CH_4 and CO_2 conversion rates of 0.174 mmol s^{-1} g_{Ni}^{-1} and 0.169 mmol s^{-1} g_{Ni}^{-1}, respectively. Notably, owing to their comparable conversion rates, the

H/C molar ratio in the syngas products are determined as 1.05 on $LaNi_{0.8}Al_{0.2}O_3/CaO$ DFM. Nevertheless, the H/C ratio of 1.89 obtained on Ni/CaO DFM indicates the mismatched conversions between CO_2 and CH_4, which originates from pronounced CH_4 cracking over its larger Ni NPs.

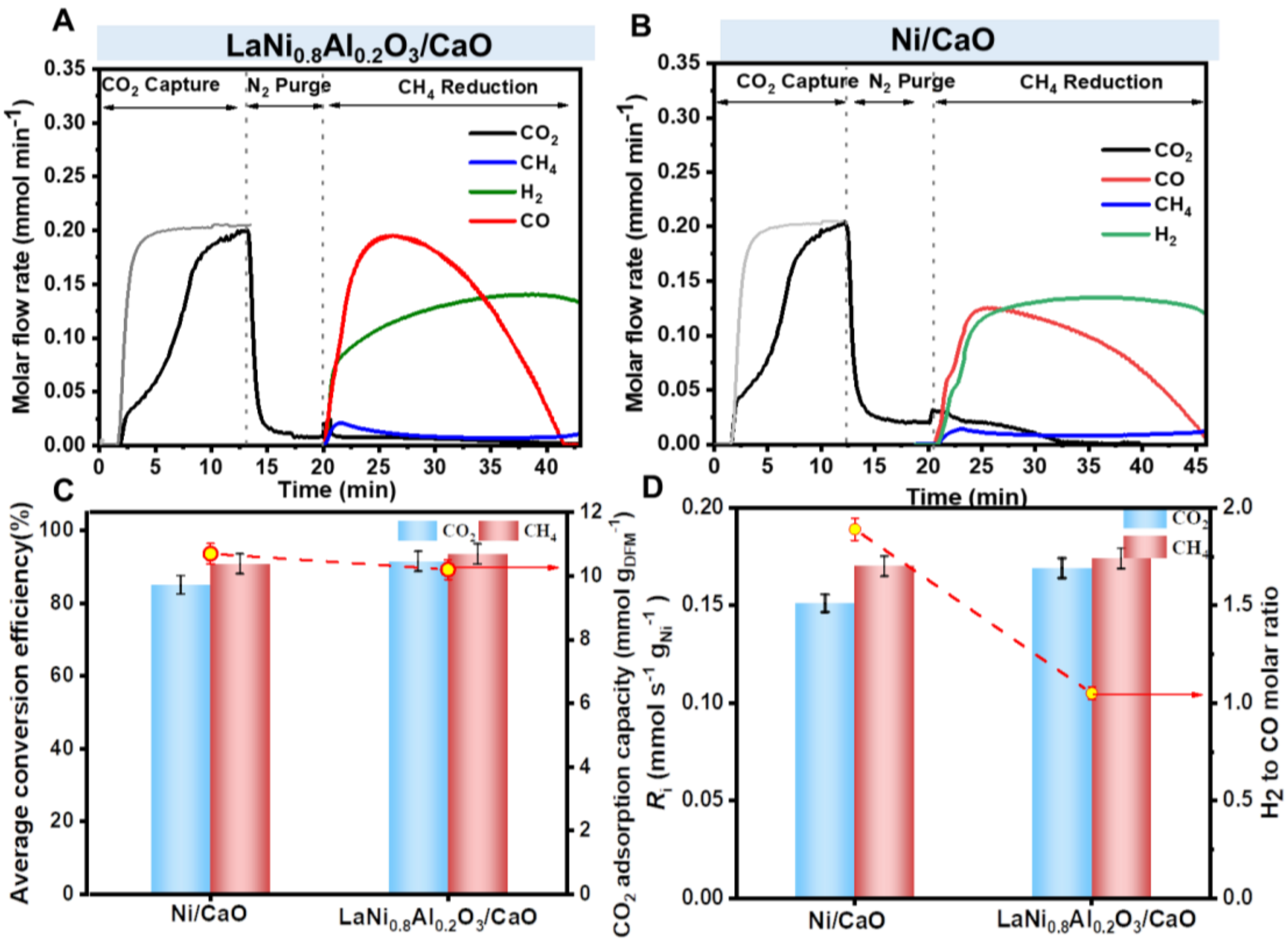

Figure R5 (Figure S16). Typical molar flow rate of each component in the effluent gas during one cycle of iCCC-DRM on (a) $LaNi_{0.8}Al_{0.2}O_3/CaO$ and Ni/CaO DFMs. The effects of Al-doping on (c) average conversion of CO_2 and CH_4 along with CO_2 capture capacity, and (d) average conversion rate (R_i) of CO_2 and CH_4 along with the H_2-to-CO molar ratio.

Consequently, 25-cyclic iCCC-DRM test on $LaNi_{0.8}Al_{0.2}O_3/CaO$ DFM demonstrates remarkable stabilities in both CO_2 capture capacity and conversion efficiencies of CO_2 and CH_4, maintaining at 10.2 mmol g^{-1} capacity, and 91.5% and 93.5%, without any decay, respectively (**Figure R6**). On the contrary, the Ni/CaO DFM shows rapid decreases in CO_2 capture capacity, from 10.7 mmol g_{DFM}^{-1} to 4.8 mmol g_{DFM}^{-1} with 55% decline after 25 cycles. Meanwhile, the conversion efficiencies of CH_4 and CO_2 decreases from 90.8% to 52.1% and 85.0% to 72.0%, respectively. During each cycle of conventional Ni/CaO DFM, the reversible phase transformation between CaO and $CaCO_3$ weakens the metal-support interaction of Ni-CaO, thereby inducing severe sintering of Ni NPs. After 25 cycles, TEM characterization reveals that the

average particle size of Ni nanoparticles increases to 36.1 nm, and many filamentous carbon depositions can be observed (**Figures R7** and **R8**). In distinct contrast, the spend $LaNi_{0.8}Al_{0.2}O_3/CaO$ DFM after 25 cycles exhibits almost the same morphology of the fresh one, without any particle aggregations and carbon deposition. Therefore, with the coherent high Tammann temperature (~800 °C) of $LaNi_{0.8}Al_{0.2}O_3$ perovskite, the spatial separation of CaO domains collectively prevents sintering during volumetric expansion. More significantly, the confinement effect of Al-doped $LaNiO_3$ perovskite ensures the reversible dissolution-exsolution of Ni NPs during the dynamically switched redox iCCC-DRM process. As a result, the durability of both CO_2 capture and in-situ conversion are synergistically boosted.

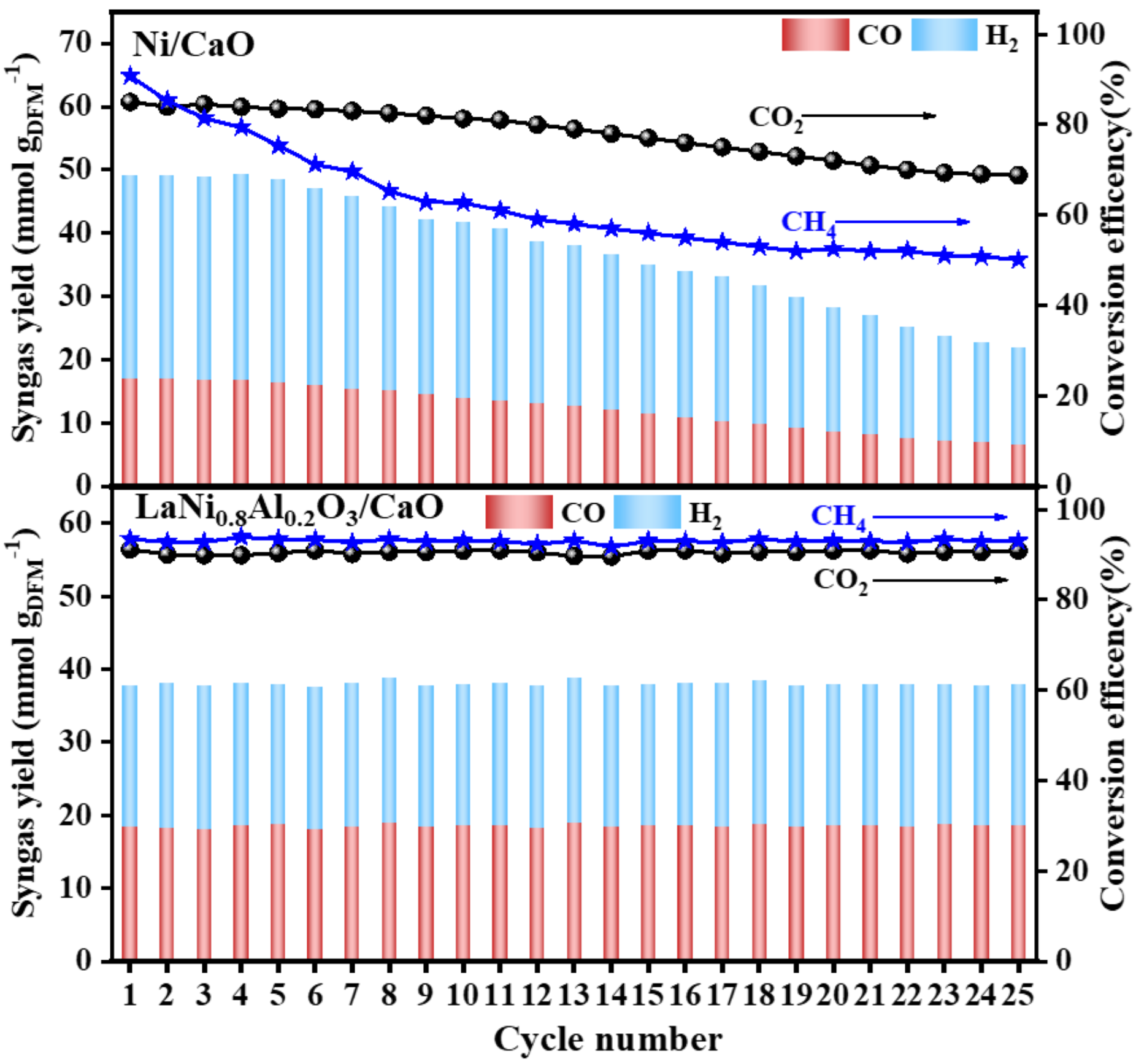

Figure R6 (**Figure S18**). 25 cyclic iCCC-DRM stability of Ni/CaO and $LaNi_{0.8}Al_{0.2}O_3/CaO$ DFMs at 650°C. Operating conditions: at the CO_2 capture stage, the inlet gas was 10 vol% CO_2 and 2 vol%O_2 simulated flue gas, after N_2 purging, the inlet gas at the conversion stage was switched into 5 vol% CH_4 at GHSV of 30000 mL g^{-1} h^{-1}.

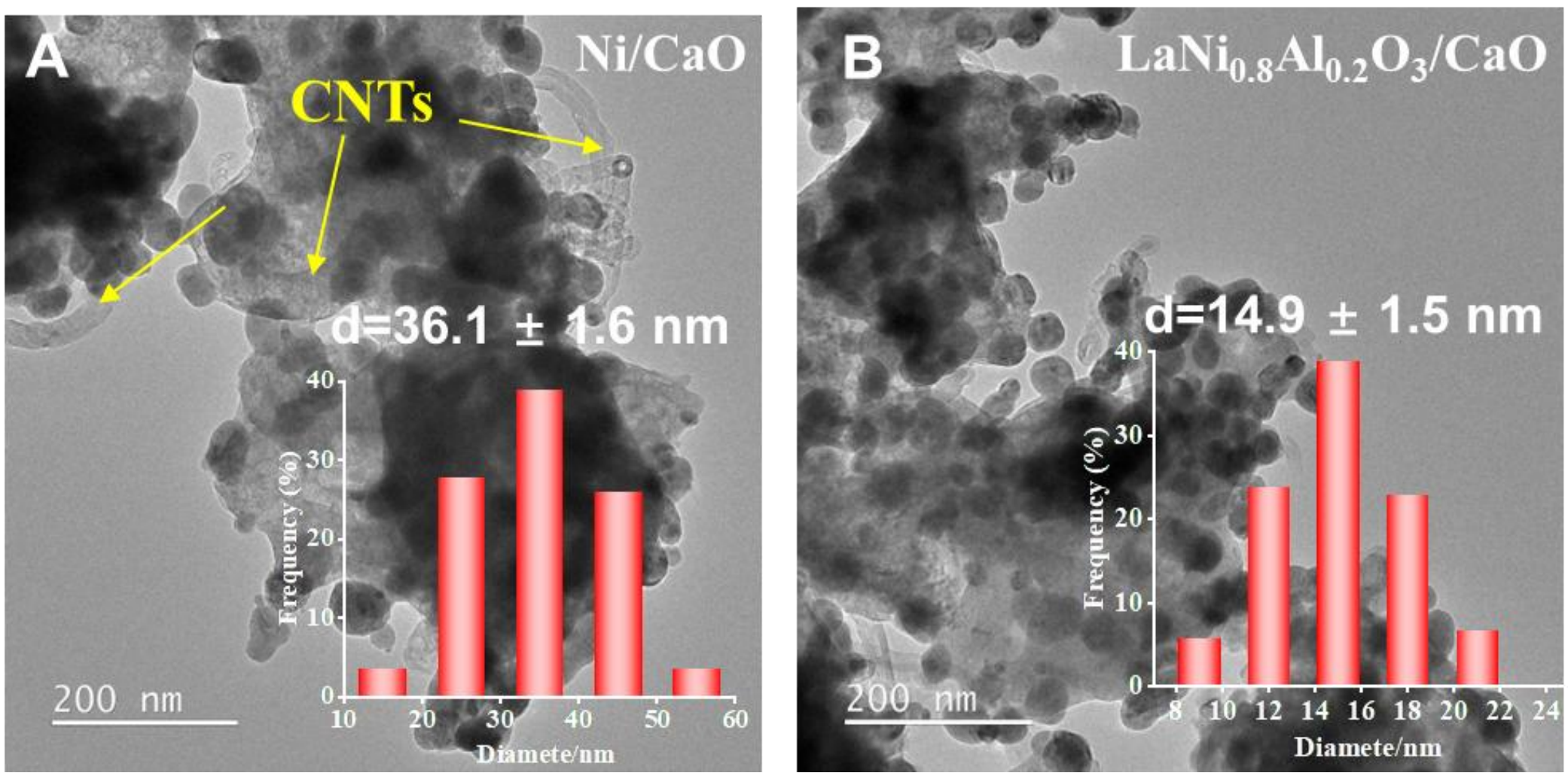

Figure R7 (Figure S19). (a)TEM image of spent Ni/CaO after 25 cycles; (b) TEM image of spent $LaNi_{0.8}Al_{0.2}O_3$/CaO after 25 cycles.

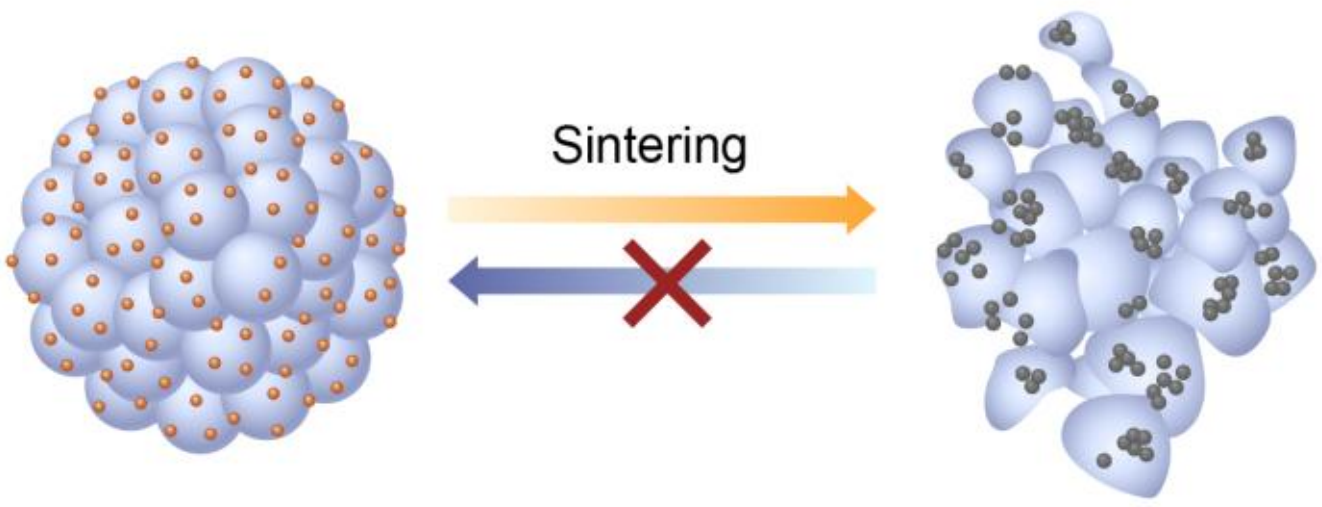

Figure R8 Schematics of iCCC-DRM over conventional Ni/CaO DFM for sintering and coke deposition.

In order to clarify the advantages of the proposed perovskite $LaNi_{1-x}Al_xO_3$/CaO DFMs, we have supplied Figures R1, R2, R4, R6, R7, R8 as Figures S13(a, b), S5, S16, S18 and S19 in the revised Supporting Information, and added more corresponding discussions in the revised manuscript.

(**Page 16, Lines 266-273**) "To highlight the interfacial synergistic effect between CaO adsorption sites and perovskite catalytic sites of the DFM, the control samples with the fixed Ni content of pristine Ni/CaO, $LaNiO_3$/CaO without Al-doping, as well as physical mixing DFM of $LaNi_{0.8}Al_{0.2}O_3$/CaO-PM were also prepared and demonstrated (**Figures S13-15**). Significantly, after 25 cycles, the CO_2 capture capacity of Ni/CaO DFM rapidly decreases from initial 10.7 mmol g_{DFM}^{-1} to 4.8 mmol g_{DFM}^{-1} with 55% decline, and the CH_4 conversion efficiency decreases from 65.9% to 52.1% (**Figures S17 and S18**). During each cycle of conventional Ni/CaO DFM, the reversible phase transformation between CaO and $CaCO_3$ weakens the metal-support interaction of Ni-

CaO, thereby inducing severe sintering of Ni NPs and filamentous carbon depositions (**Figure S19**).”

(Page 25, Lines 425-429) “…For comparing, Ni/CaO DFM was also synthesized by using the impregnation method without the introduction of La and Al, and the Ni content was maintained at 5 wt%. ”

Question 2: *“In the iCCC process, CO_2 capture is primarily governed by CaO. Although the authors attribute enhanced CO_2 adsorption kinetics to the increased oxygen vacancy (O_v) concentration in Al-doped $LaNiO_3$, this effect is inherently limited to the perovskite phase. Therefore, it remains unclear how the O_v in La(Ni,Al)O_3 significantly influences the overall CO_2 capture behavior, which is expected to be dominated by CaO. Furthermore, I think Figure 5A does not sufficiently support the formation of O^{2-} channels as claimed. The authors should more clearly elucidate how oxygen vacancies in La(Ni,Al)O_3 contribute to the adsorption mechanism. Revision of the figure may also be necessary to better support this claim.”*

Response: We thank the reviewer for raising this concern. We fully agree with the comments that the overall CO_2 capture behavior is dominated by CaO, because the CO_2 adsorption capacity of the $LaNi_{0.8}Al_{0.2}O_3$ catalyst was negligible (0.0069mmol g^{-1}) at 650°C in comparison with that of 10.1 mmol g^{-1} of pure CaO sorbent (**Figure R9**).

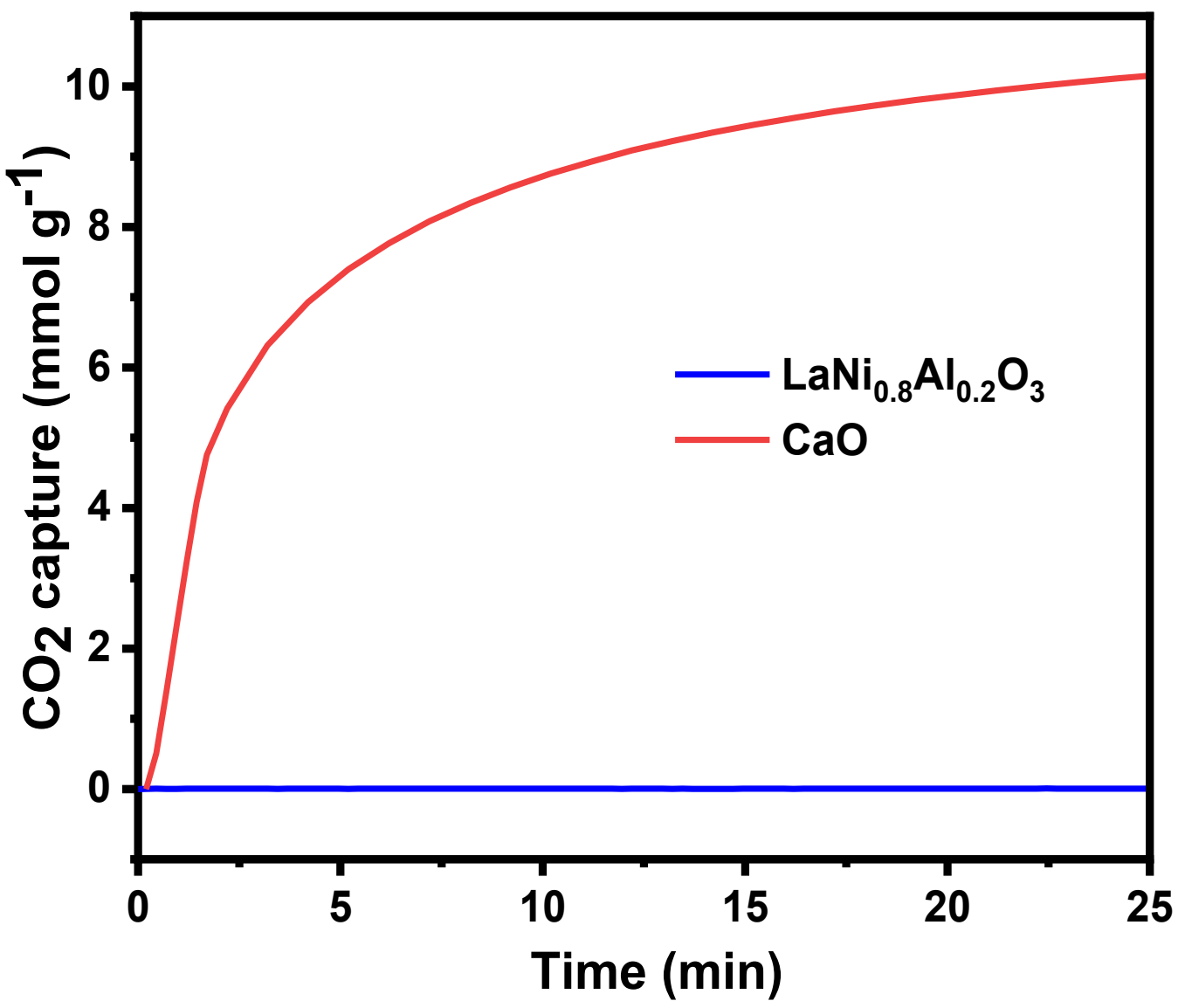

Figure R9 CO_2 adsorption capacity of CaO sorbent and $LaNi_{0.8}Al_{0.2}O_3$ perovskite.

The actual carbonation is a complex three-step process including ① surface adsorption of CO_2, ② reaction with O^{2-} to generate CO_3^{2-}, and ③ ion exchange with

CaO to form $CaCO_3$. Accordingly, the O^{2-} specie plays a significant bridge role in the CO_2 adsorption kinetics. Naturally, to keep electrical neutrality, O^{2-} species will migrate outward, in the opposite direction of inward bulk diffusion of CO_3^{2-} to accommodate CO_2 molecules (**Figure R10**). However, such O^{2-} migration proceeds sluggishly owing to lack of additional driving force, resulting in the serious retarded CO_2 diffusion caused by the in-situ formed dense $CaCO_3$ layer over CaO. It has been reported that oxygen vacancies (O_v) can facilitate the CO_2 adsorption by the insertion, thus providing the active O^{2-} species (*Ind. Eng. Chem. Res., 2022, 61: 5527-5535*). This can accelerate the O^{2-} migration through the $CaCO_3$ product layer via the O_v hopping mechanism (*Chem Catal., 2023, 3: 100785*). Accordingly, introducing O_v in CaO-based adsorbent can serve as a promising strategy to address the challenge of sluggish CO_2 diffusion through the $CaCO_3$ layer.

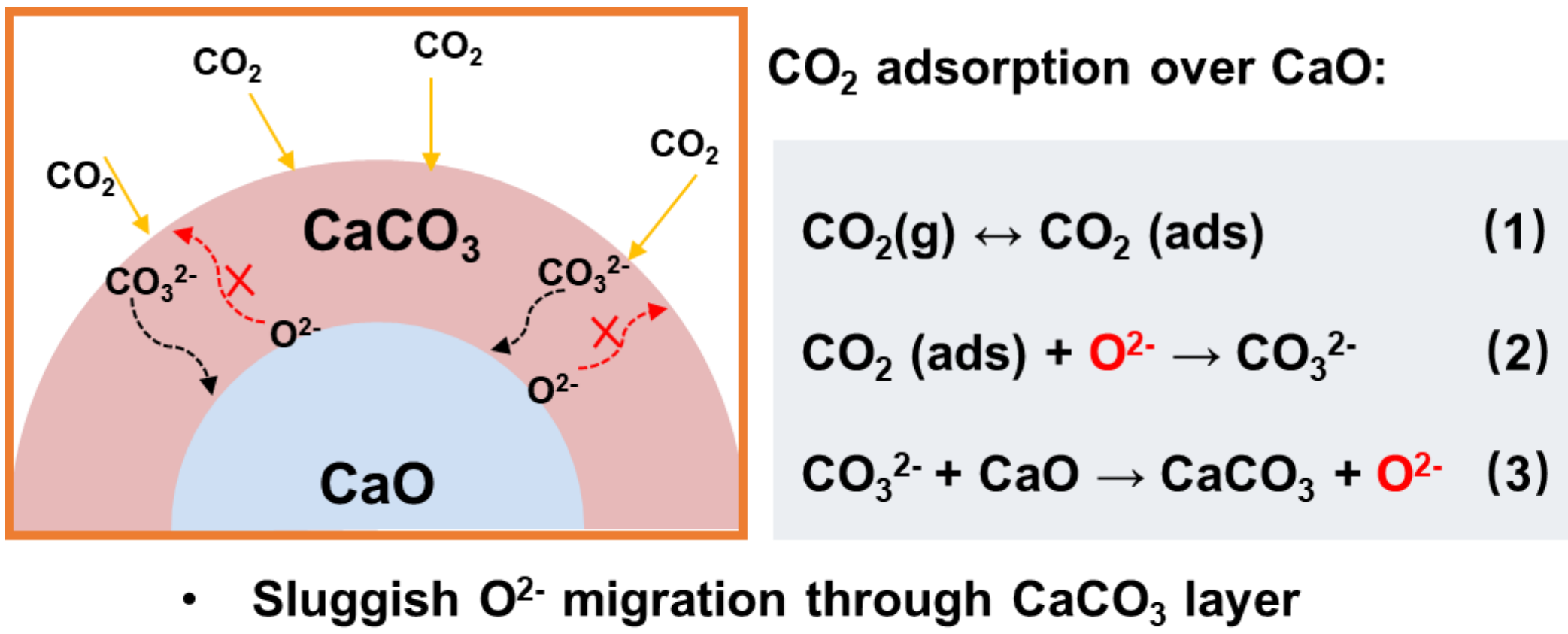

Figure R10 (Figure 5A) CO_2 adsorption mechanism of CaO-based adsorbents.

Notably, ABO_3 perovskite is a typical intrinsic oxygen-deficient crystalline, especially when the B site is partially substituted by another metal. The metal doping will induce the open BO_6 octahedral framework more distorted, which can be assessed by the octahedron factor ($\mu = r_B/r_O$), the ratio of average radius of B cations over that of O anion. With increasing the amount of Al substitution, the μ of $LaNi_{1-x}Al_xO_3$ perovskite decreases from 0.400 to 0.391 (**Table S4**), revealing more lattice distortion and hence more O_vs for facilitating O^{2-} migration. Therefore, we highly anticipate through one-pot sol-gel method, the $LaNi_{1-x}Al_xO_3$/CaO DFMs can produce abundant O^{2-} migration channels.

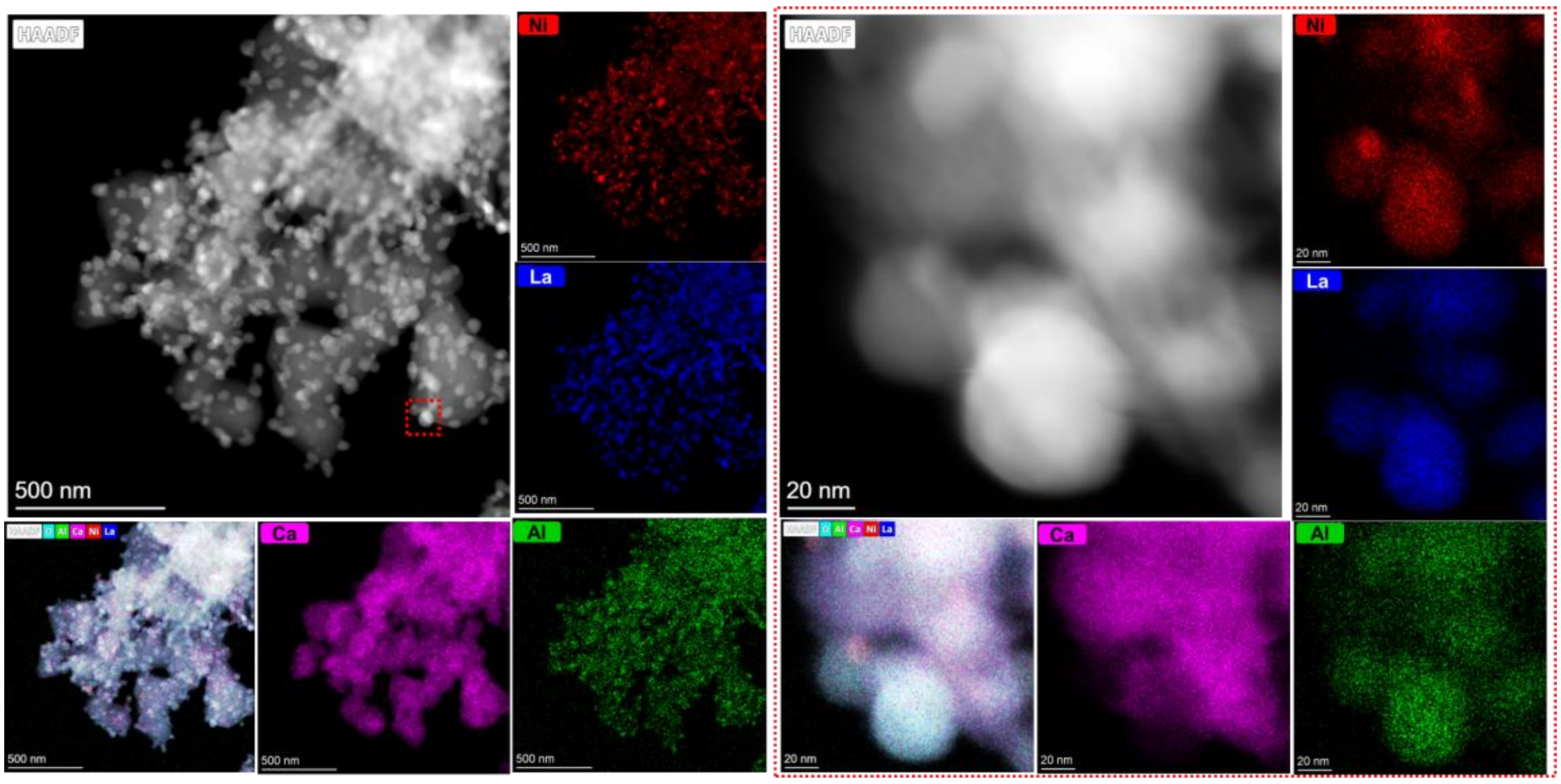

Figure R11(Figure S3) STEM-HAADF and EDS elemental mapping images of the as-synthesized $LaNi_{0.8}Al_{0.2}O_3$/CaO DFM.

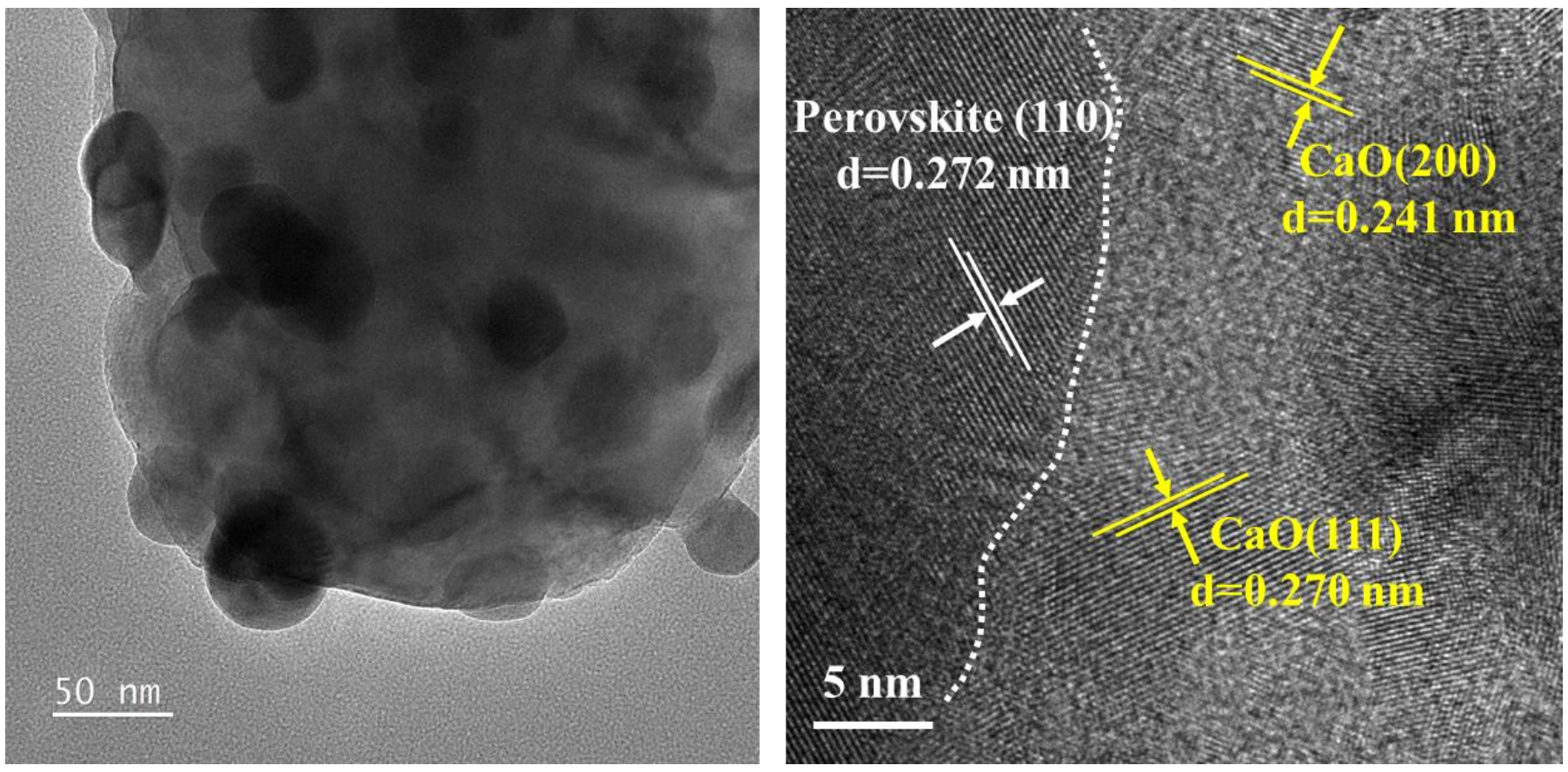

Figure R12(Figure S21) HRTEM images of as-synthesized $LaNi_{0.8}Al_{0.2}O_3$/CaO DFM with intimately contacted heterogeneous interfaces.

The STEM-HAADF and EDS elemental mapping images in different scales all verify the well dispersed $LaNi_{1-x}Al_xO_3$ perovskite particles in CaO matrix (**Figure R11**). Meanwhile, the intimately contacted heterogeneous interfaces between $LaNi_{1-x}Al_xO_3$ perovskite and CaO are further verified by HRTEM image (**Figure R12**). The electron paramagnetic resonance (EPR) spectra of $LaNi_{0.5}Al_{0.5}O_3$/CaO further exhibits the strongest signal response at g = 2.004, positively correlated with the concentrated O_vs (**Figure R13**). Accordingly, the CO_2 adsorption mechanism in $LaNi_{1-x}Al_xO_3$/CaO DFM is demonstrated. As illustrated in **Figure R14**, the abundant O_vs original from $LaNi_{1-x}Al_xO_3$ can act as the driving force to induce reactive O^{2-} migration: that O^{2-} migrates to adjacent O_v, leaving a new O_v at the original lattice site and subsequently reoccupied by surrounding O^{2-}. In this way, O_vs are dynamically occupied and regenerated,

providing O^{2-} transport channels for continuously accelerating CO_2 diffuse across the interface and react with inner CaO to sustain excellent CO_2 capture performance.

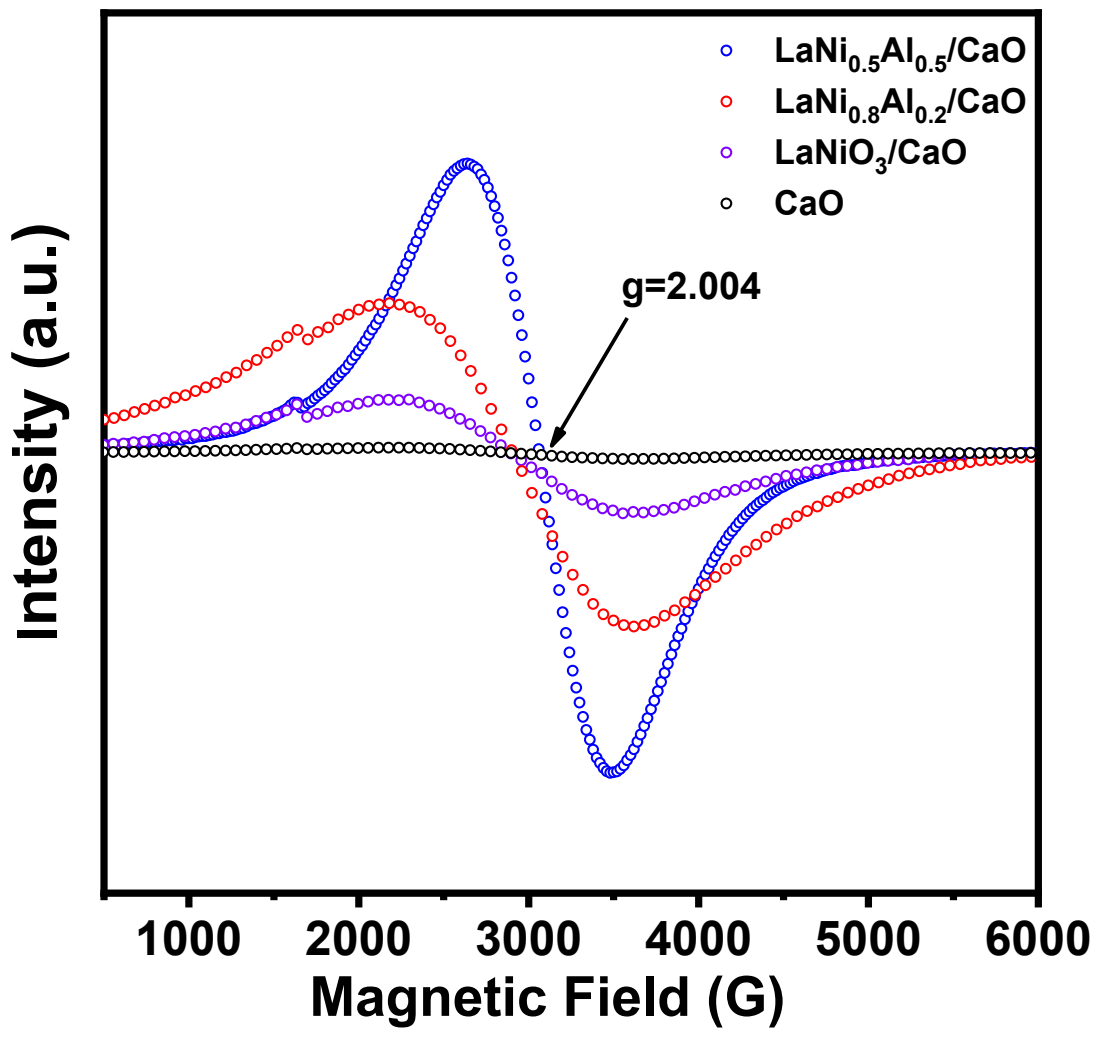

Figure R13 (Figure S9) EPR curves of CaO sorbent and $LaNi_{1-x}Al_xO_3$/CaO DFMs.

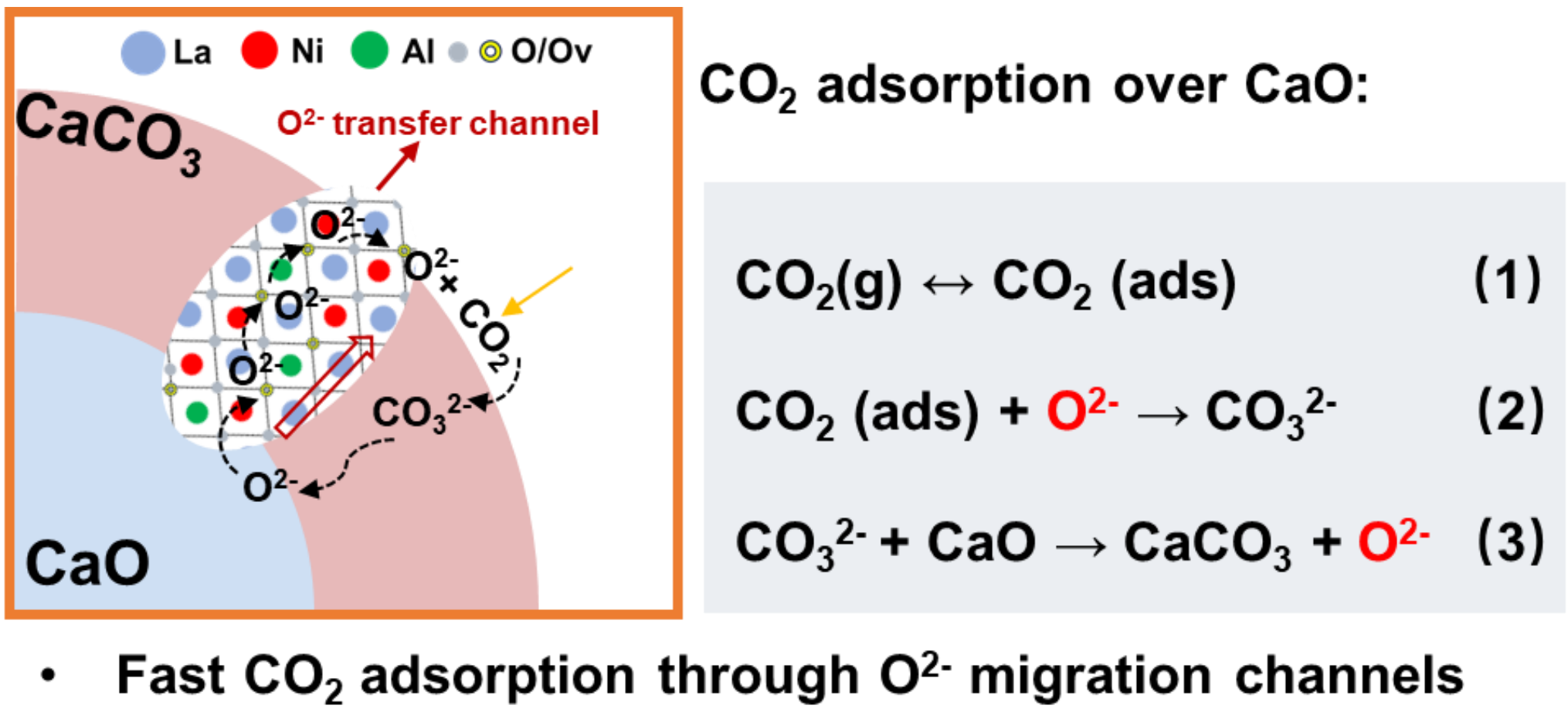

Figure R14 (Figure 5A) Schematic diagrams of the role of O^{2-} migration channels in DFMs for CO_2 adsorption.

As expected, the CO_2 adsorption rate increases with incorporation of Al-doping perovskites (**Figure R15a**). Each adsorption kinetic curves at 650 °C was well simulated by the double exponential model (**Table S5**). Notably, the initial fast chemical adsorption (carbonation) rate constant (k_1) and subsequent CO_2 diffusion-controlled rate constant (k_2) of $LaNi_{0.5}Al_{0.5}O_3$/CaO (4.17 min^{-1}, 0.25 min^{-1}) are 3-6 times larger than those of CaO (0.72min^{-1}, 0.09min^{-1}), respectively. Importantly, we have identified a remarkable linear relationship between k_1(k_2) and the intensity of O_v EPR signal, verifying the concentration of O_v directly governs the O^{2-} migration barrier and accelerate CO_2 diffusion (**Figure R15b**).

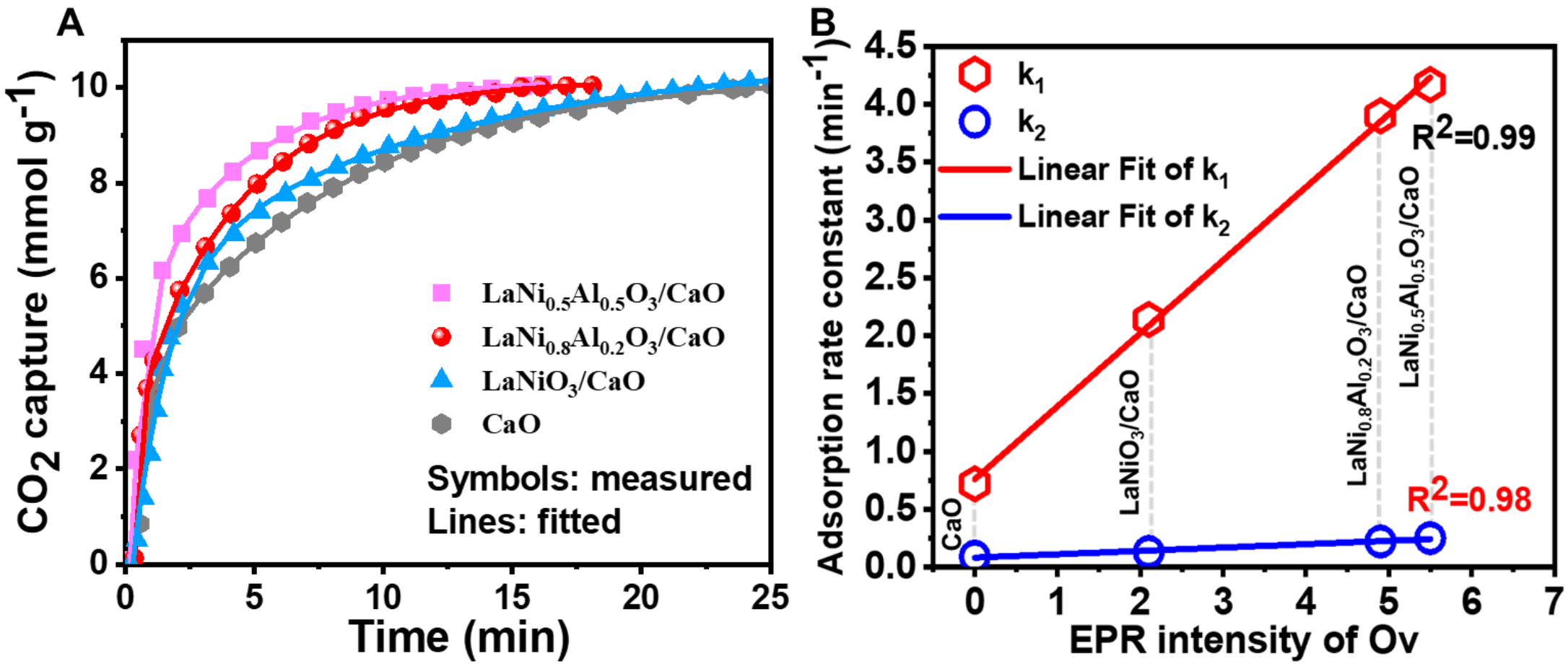

Figure R15 (Figure S22 and Figure 5C) (a) Experimental and kinetic fittings of the CO_2 adsorption on fresh CaO sorbent and $LaNi_{1\text{-}x}Al_xO_3$/CaO DFMs. (Test conditions: 650 °C, 10vol% CO_2 /2vol%O_2 /N_2). (b) the regression fitting between k_1(k_2) and the intensity of O_v EPR signal of CaO and $LaNi_{1\text{-}x}Al_xO_3$/CaO DFMs.

Therefore, we can conclude $LaNi_{1\text{-}x}Al_xO_3$ perovskite phase in DFMs boosts the CO_2 adsorption kinetics through providing abundant O^{2-} migration channels in CaO matrix. What's more, it also plays a synergistic role to enhance CO_2 adsorption stability through restraining the agglomeration of CaO grains by intimate interfacial contact.

Table S6 Kinetic parameters of CO_2 adsorption on CaO sorbent and Al-doped $LaNiO_3$/CaO DFMs at 650°C.

Samples	A1	A2	A3	k_1 / min^{-1}	k_2 / min^{-1}	R^2
CaO	10.68	-7.36	-4.78	0.72	0.09	0.997
$LaNiO_3$/CaO	10.27	-7.13	-6.77	2.14	0.13	0.999
$LaNi_{0.8}Al_{0.2}O_3$/CaO	10.15	-12.72	-7.40	3.90	0.22	0.999
$LaNi_{0.5}Al_{0.5}O_3$/CaO	10.17	-13.28	-6.89	4.17	0.25	0.999

Accordingly, in the revised manuscript, we have revised the original Figure 5A and added Figures R10 and R14, R11, R12, R13, and R15 as Figures 5A, S3, S21, S9, S22 and Figure 5D, and elucidated the oxygen vacancies contributions to the enhanced CO_2 adsorption on $LaNi_{1\text{-}x}Al_xO_3$/CaO DFM as the following.

(Page 17, Lines 284-299) "The conventional carbonation consists of three steps ①

surface adsorption of CO_2, ② reaction with O^{2-} to generate CO_3^{2-}, and ③ ion exchange with CaO to form $CaCO_3$. Naturally, O^{2-} species will migrate outward to accommodate CO_2 molecules. However, such O^{2-} migration proceeds sluggishly owing to lack of driving force, resulting in the serious retarded CO_2 diffusion caused by the in-situ formed dense $CaCO_3$ layer over CaO (**Figure 5A**). It has been reported that oxygen vacancies (O_v) can facilitate the CO_2 adsorption via the O_v hopping mechanism [53–55], which accelerates the O^{2-} migration through the $CaCO_3$ product layer. Accordingly, introducing O_v in CaO-based adsorbent can serve as a promising strategy to address the challenge of sluggish CO_2 diffusion through the $CaCO_3$ layer.

Notably, with increasing the amount of Al substitution, the octahedron factor (μ) of $LaNi_{1-x}Al_xO_3$ perovskite in DFMs decreases from 0.400 to 0.391 (**Table S4**), revealing more lattice distortion and hence more O_vs. Thanks to the intimately contacted between $LaNi_{1-x}Al_xO_3$ perovskite and CaO (**Figure S21**), the abundant O_vs generated in $LaNi_{1-x}Al_xO_3$ can act as the driving force to induce reactive O^{2-} migration. Specifically, O^{2-} migrates to adjacent O_v, leaving a new O_v at the original lattice site and subsequently reoccupied by surrounding O^{2-}. In this way, O_vs are dynamically occupied and regenerated, providing O^{2-} transport channels for CO_2 to diffuse across the $CaCO_3$ layer and react with inner CaO."

(Page 18, Lines 305-313) "This enhanced CO_2 adsorption kinetic was illustrated by the double exponential model (**Eq. S1,** see in the supplementary information), that at 650 °C, the initial fast chemical adsorption (carbonation) rate constant (k_1) and subsequent CO_2 diffusion-controlled rate constant (k_2) of $LaNi_{0.5}Al_{0.5}O_3$/CaO (4.17 min^{-1}, 0.25 min^{-1}) are 3-6 times larger than those of CaO (0.72min^{-1}, 0.09min^{-1}), respectively (**Figure S22** and **Table S6**). Importantly, we have identified a remarkable linear relationship between k_1, or k_2 and the intensity of O_v EPR signal (**Figure 5C**) verifying the concentration of O_v directly governs the O^{2-} migration barrier and accelerate CO_2 diffusion. Therefore, $LaNi_{1-x}Al_xO_3$ perovskite phase in DFMs boosts the CO_2 adsorption kinetics through providing abundant O^{2-} migration channels in CaO matrix."

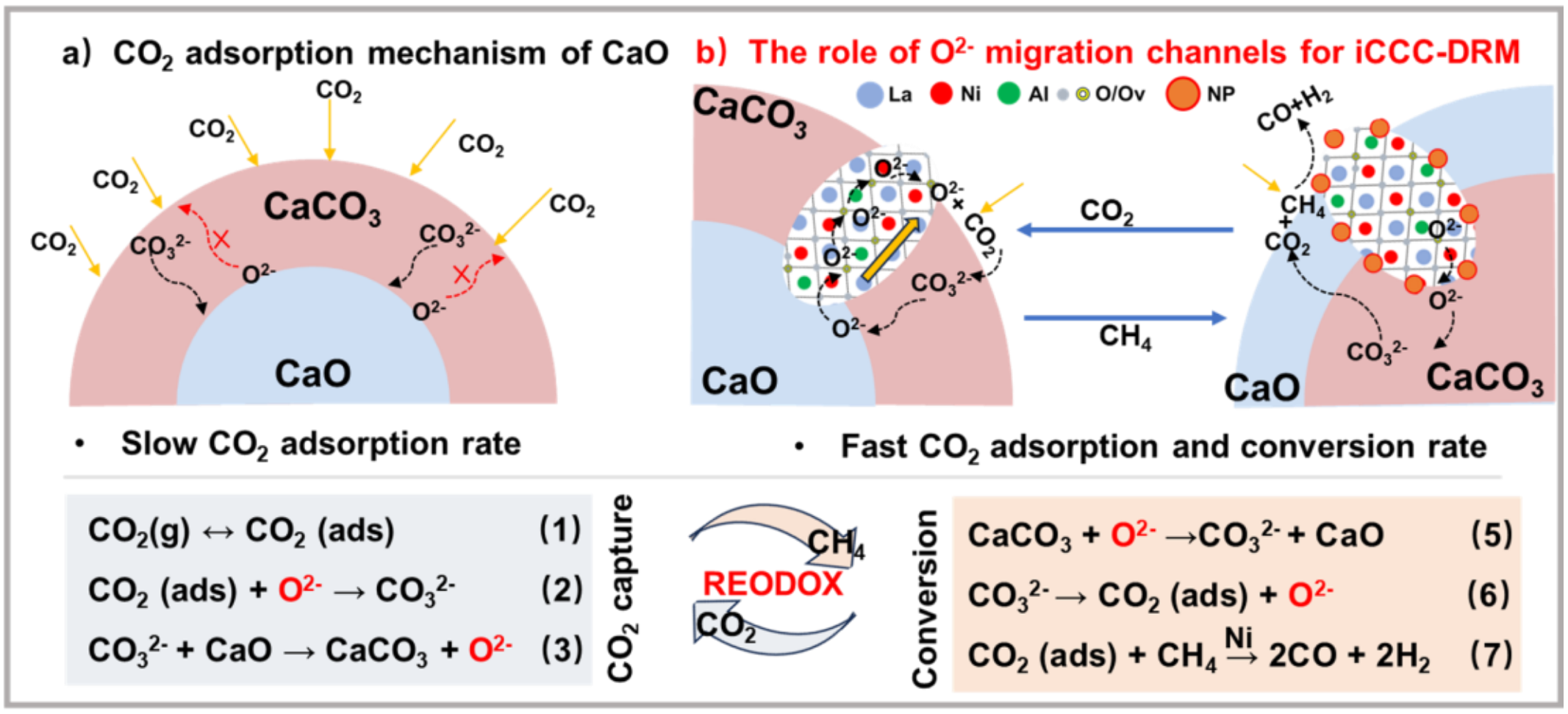

Figure 5A. Schematic diagrams of the role of O^{2-} migration channels in DFMs for iCCC-DRM.

Question 3: "*Following the above point, a control experiment using a simple physical mixture of Al-doped* $LaNiO_3$ *and CaO would be highly beneficial. Such a comparison would help clarify whether the observed performance enhancement originates from the proposed one-pot synthesis and structural integration, rather than from a simple combination of the two components.*"

Response: We highly appreciate the reviewer's valuable suggestions. Following the suggestions, we synthesized the Al-doped $LaNiO_3$ ($LaNi_{0.8}Al_{0.2}O_3$) catalyst and CaO sorbent, respectively, and subsequently physically mixed them together to keep Ni loading at about 5.0 wt% (**Table S3**), namely $LaNi_{0.8}Al_{0.2}O_3$/CaO-PM DFM. The XRD patten of the $LaNi_{0.8}Al_{0.2}O_3$/CaO-PM DFM displays characteristic diffraction peaks of the perovskite $LaNi_{1-x}Al_xO_3$ (PDF#34-1181) and CaO (PDF#48-1467) (**Figure R16**). While STEM-HAADF EDS mapping images demonstrates the spatial separated $LaNi_{0.8}Al_{0.2}O_3$ and CaO two phases through the physical mixing (**Figure R17**). Providing with an identical Ni content, the initial CO_2 adsorption capacity of $LaNi_{0.8}Al_{0.2}O_3$/CaO-PM is the same as the one-pot synthesized $LaNi_{0.8}Al_{0.2}O_3$/CaO. However, without any spatial segments, the CaO in physical mixing approach triggers rapid sintering during the recycle carbonation-decarbonation process, consequently results in a significant decay of 77.2 % in adsorption capacity after 25 cycles (**Figure R18**). Even worse, the CH_4 conversion efficiency is as low as 28.9% on $LaNi_{0.8}Al_{0.2}O_3$/CaO-PM DFM with a low Ni dispersion rate, and higher H/C ratio of 1.21 in the syngas production due to low Ni dispersion, which may induce carbon deposition for catalytic deactivation (**Figure R19**). This further demonstrates the crucial role of the interfacial interaction between intimate assembly of $LaNi_{0.8}Al_{0.2}O_3$

and CaO for the enhanced iCCC-DRM performance.

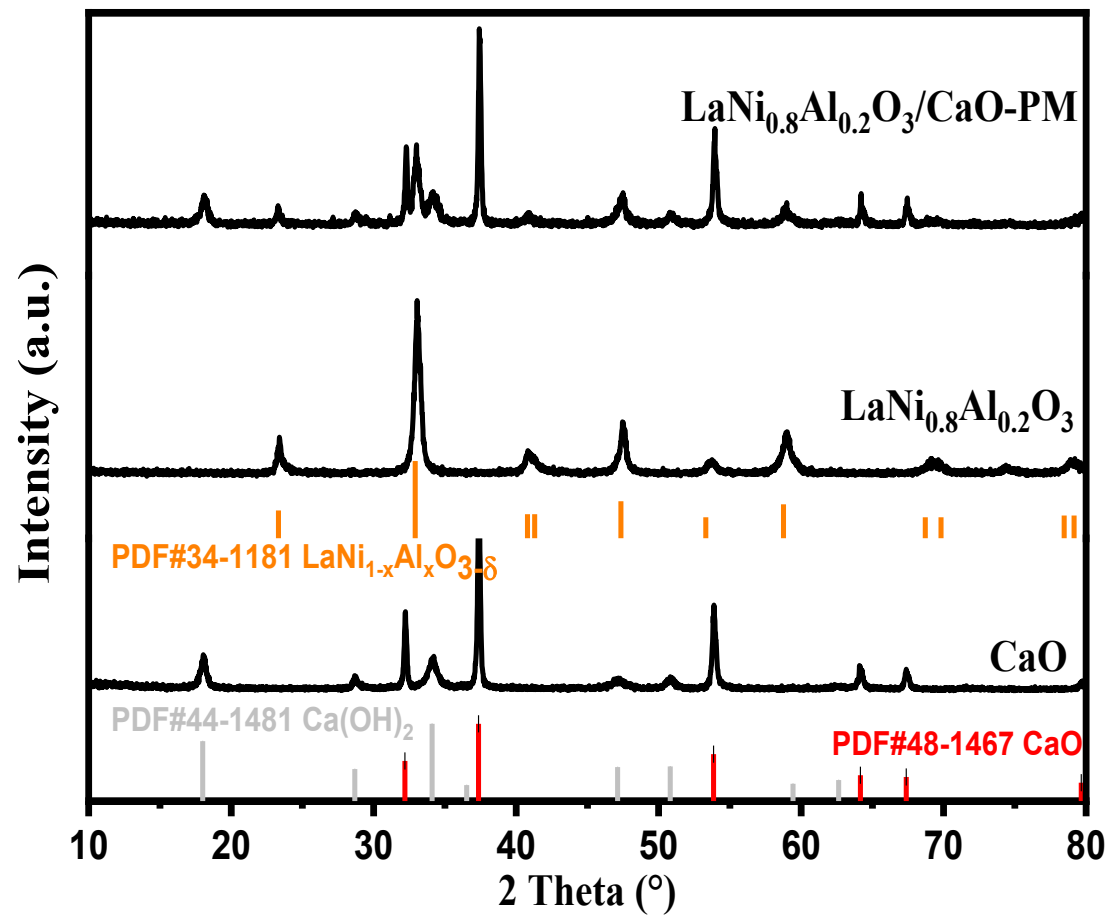

Figure R16 (Figure S14). XRD patterns of as-prepared $LaNi_{0.8}Al_{0.2}O_3$ catalyst, CaO sorbent and the $LaNi_{0.8}Al_{0.2}O_3$/CaO-PM.

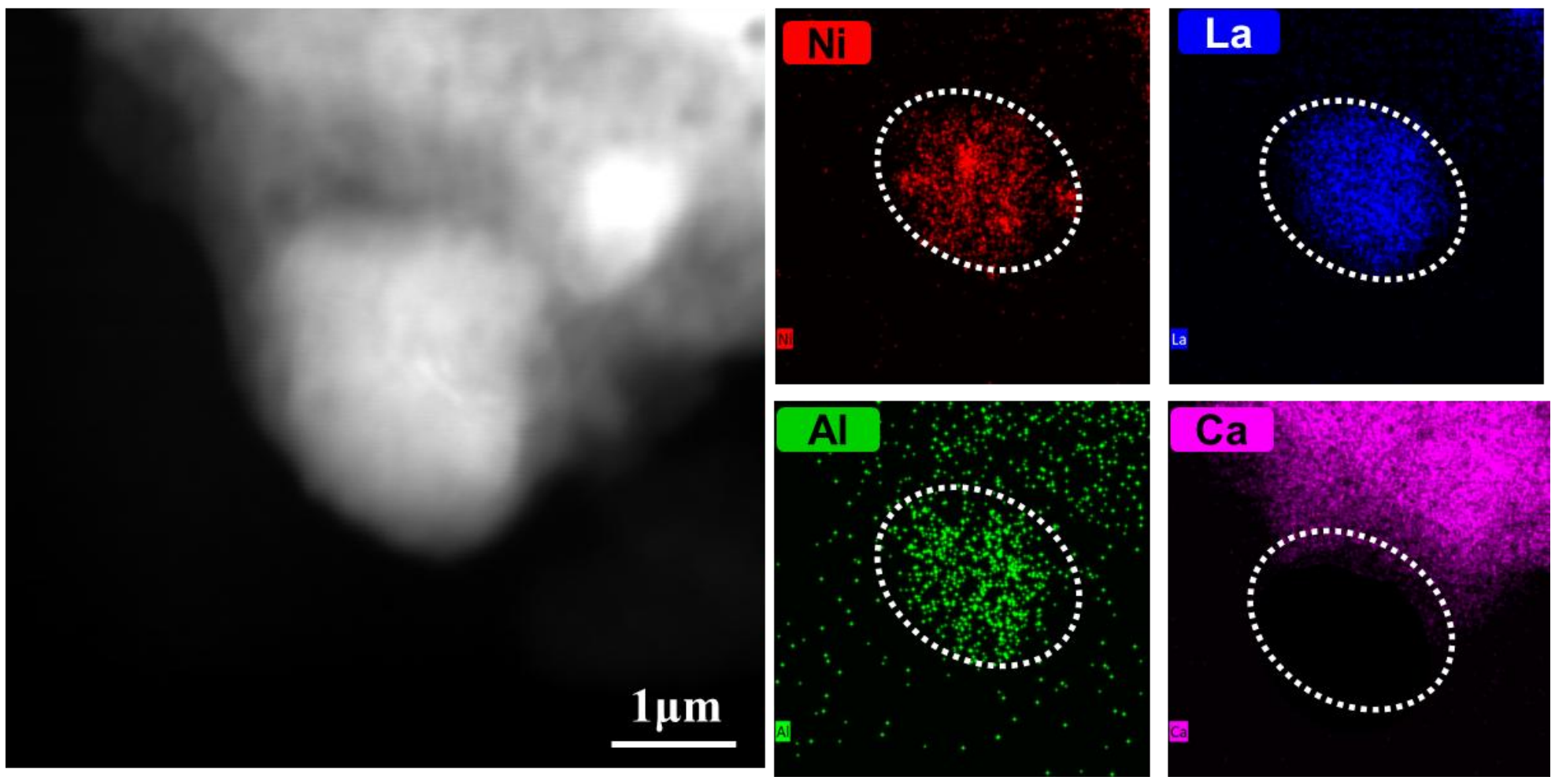

Figure R17 (Figure S15). STEM-HAADF image and EDS elemental mappings of the $LaNi_{0.8}Al_{0.2}O_3$/CaO-PM

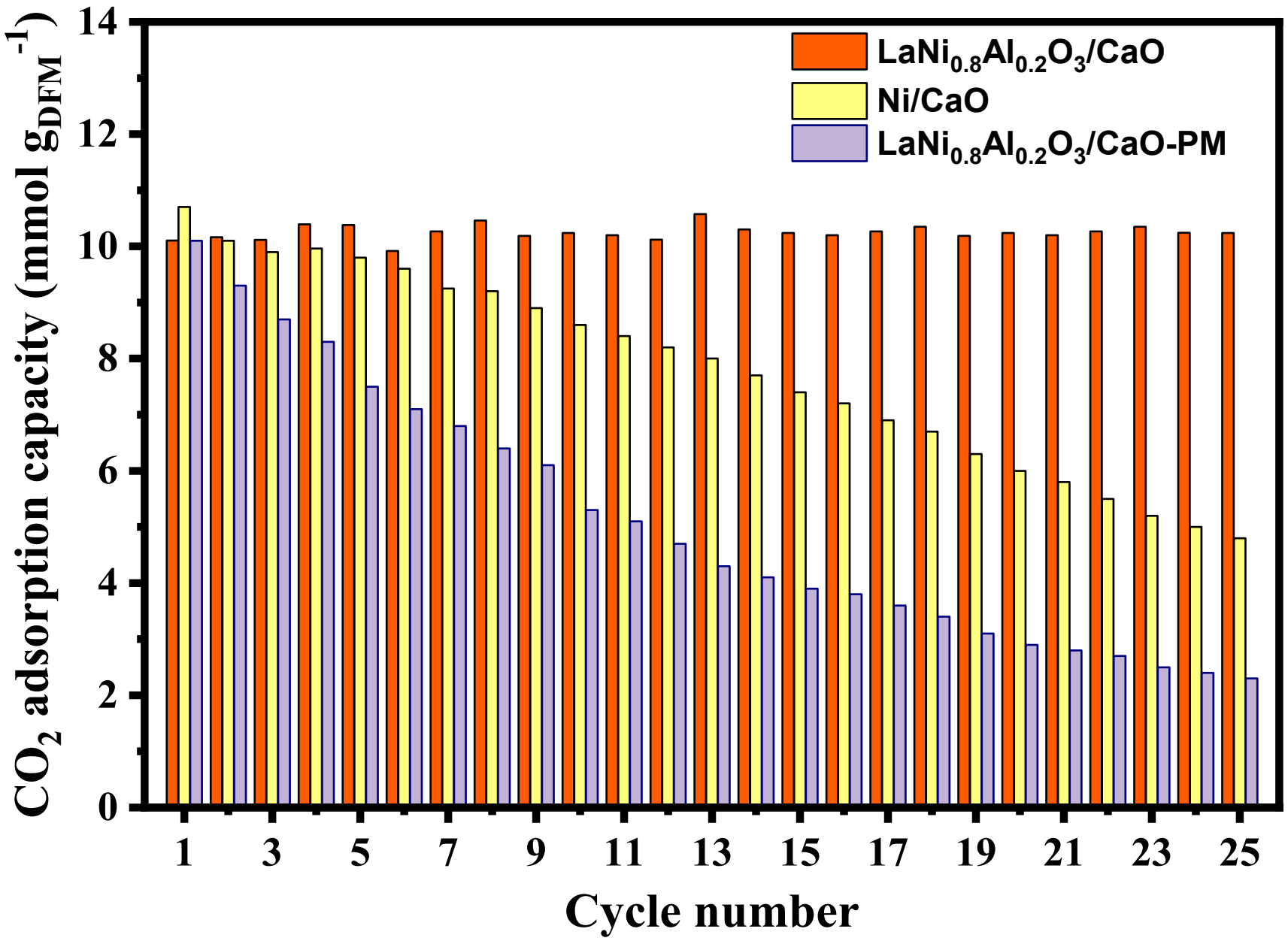

Figure R18 (Figure S17). CO_2 adsorption capacity of 25 cyclic iCCC-DRM processes on Ni/CaO, $LaNi_{0.8}Al_{0.2}O_3$/CaO and $LaNi_{0.8}Al_{0.2}O_3$/CaO-PM DFMs.

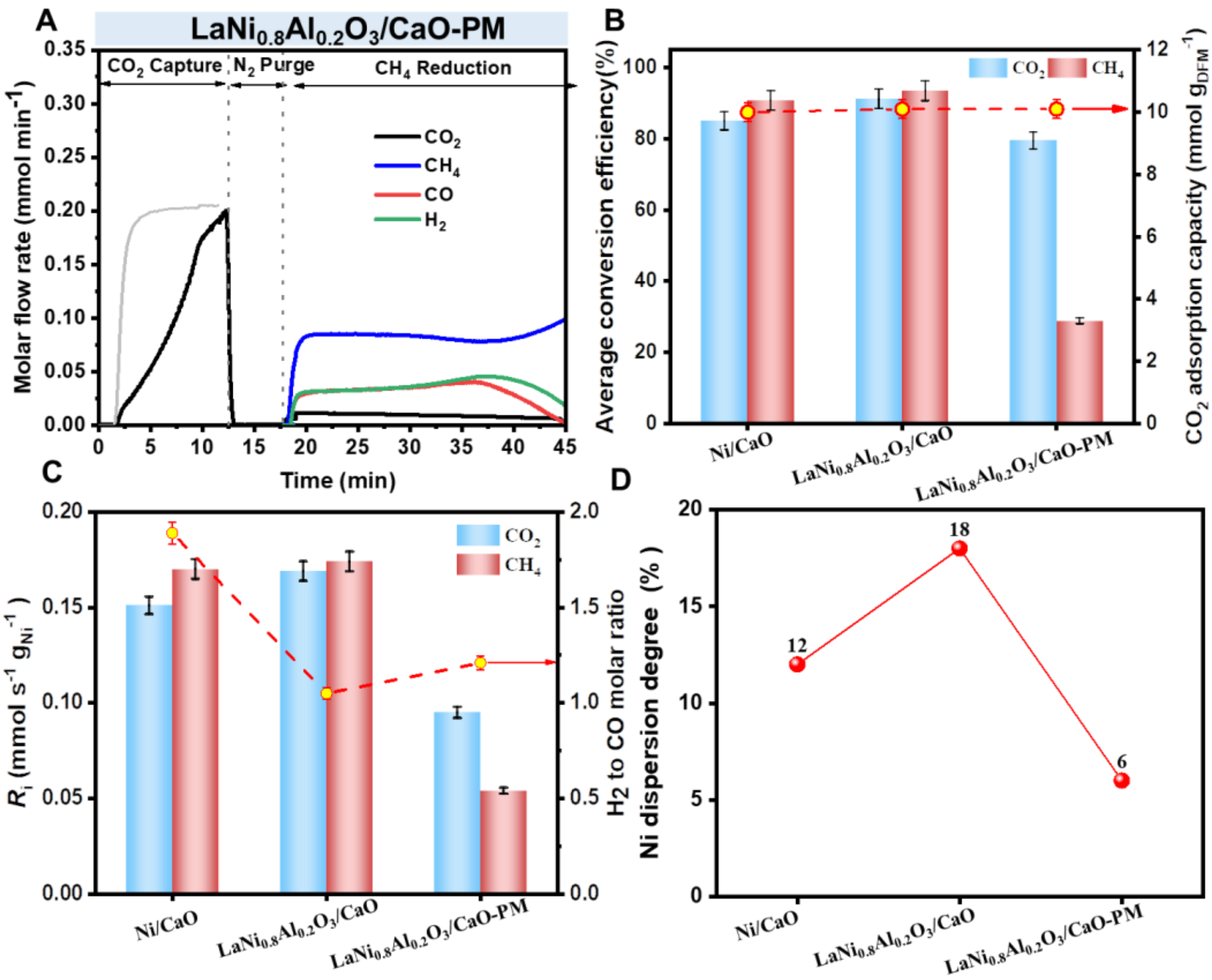

Figure R19 (Figure S16). (a) Typical molar flow rate of each component in the effluent gas during one cycle of iCCC-DRM on $LaNi_{0.8}Al_{0.2}O_3$/CaO-PM. (b) Average

conversion efficiency of CO_2 and CH_4 along with CO_2 capture capacity, and (c) average conversion rate (R_i) of CO_2 and CH_4 along with the H_2-to-CO molar ratio on different DFMs. (d) The Ni dispersion degree of different DFMs.

Accordingly, the relevant experimental results and discussion have been supplemented in the revised manuscript and revised SI.

(**Page 16, Lines 265-268**) "To highlight the interfacial synergistic effect between CaO adsorption sites and perovskite catalytic sites of the DFM, the control samples with the fixed Ni content of pristine Ni/CaO, $LaNiO_3$/CaO without Al-doping, as well as physical mixing DFM of $LaNi_{0.8}Al_{0.2}O_3$/CaO-PM were also prepared and demonstrated (**Figures S13-15**)… "

(**Page 17, Lines 277-281**) "….For comparison, physical mixing $LaNi_{0.8}Al_{0.2}O_3$/CaO-PM shows a significantly decay of 77.2% in adsorption capacity with a low CH_4 conversion efficiency of 28.9 % after 25 cycles due to low Ni dispersion, which may induce carbon deposition for catalytic deactivation (**Figures S16 - S18**). This further demonstrates the crucial role of the interfacial interaction between intimate assembly of $LaNi_{0.8}Al_{0.2}O_3$ and CaO for the enhanced iCCC-DRM performance…."

(**Page 26, Lines 427-430**) "…The Al-doped $LaNiO_3$ ($LaNi_{0.8}Al_{0.2}O_3$) catalyst and CaO sorbent were synthesized by the sol-gel method, respectively. And subsequently physically mixed them together to keep Ni loading at about 5.0 wt%, namely $LaNi_{0.8}Al_{0.2}O_3$/CaO-PM DFM. "

Question 4: "*If possible, the catalytic performance should be further evaluated in terms of intrinsic activity metrics, such as mass activity or turnover frequency (TOF), rather than relying solely on conversion values. This would allow a more rigorous assessment of the catalytic efficiency.*"

Response: We thank the reviewer for the valuable and constructive comment. According to the suggestion, we have supplemented the specific mass activity based on loading mass of Ni in the DFMs to calculate the reaction rate of CH_4 and CO_2, respectively (**Figure R20**), replaced relevant figures in Figure 4(c, e, g, i), and provided more discussions in the revised manuscript, which enables a more rigorous and objective comparison of the inherent catalytic efficiency among different samples.

(**Page 13, Lines 217-221**) "…Moreover, calculated in terms of per gram of Ni loading to improve the comparability in activity among the $LaNi_{1-x}Al_xO_3$/CaO DFMs with different Ni loadings, the $LaNi_{0.8}Al_{0.2}O_3$/CaO exhibited the superior reaction kinetics with the highest CH_4 and CO_2 conversion rates of 0.169 mmolCO_2 s^{-1} $g_{Ni}^{-1}$and 0.174

mmolCH_4 s^{-1} gNi^{-1}, respectively (**Figure 4**)…"

Moreover, the calculate method of the mass activity and the corresponding data are supplied in the revised manuscript.

(**Page 29, Lines 497-501**) "The mass activity of the average conversion rate (R_i, mmol s^{-1} g_{Ni}^{-1}) of CO_2 or CH_4 in terms of the mass of Ni loading is calculated by the following equation.

$$R_i = \frac{\int_0^{ts}[F_{i,in} - F_{i,out}(t)]dt}{M_0 \times ts}$$

Where $F_{i,in}$ and $F_{i,out}$ represents the molar flow rate of CO_2 or CH_4 in the inlet and outlet gas, t_s is the duration time of the in-situ conversion step, and M_0 is the mass of Ni loading in DFMs."

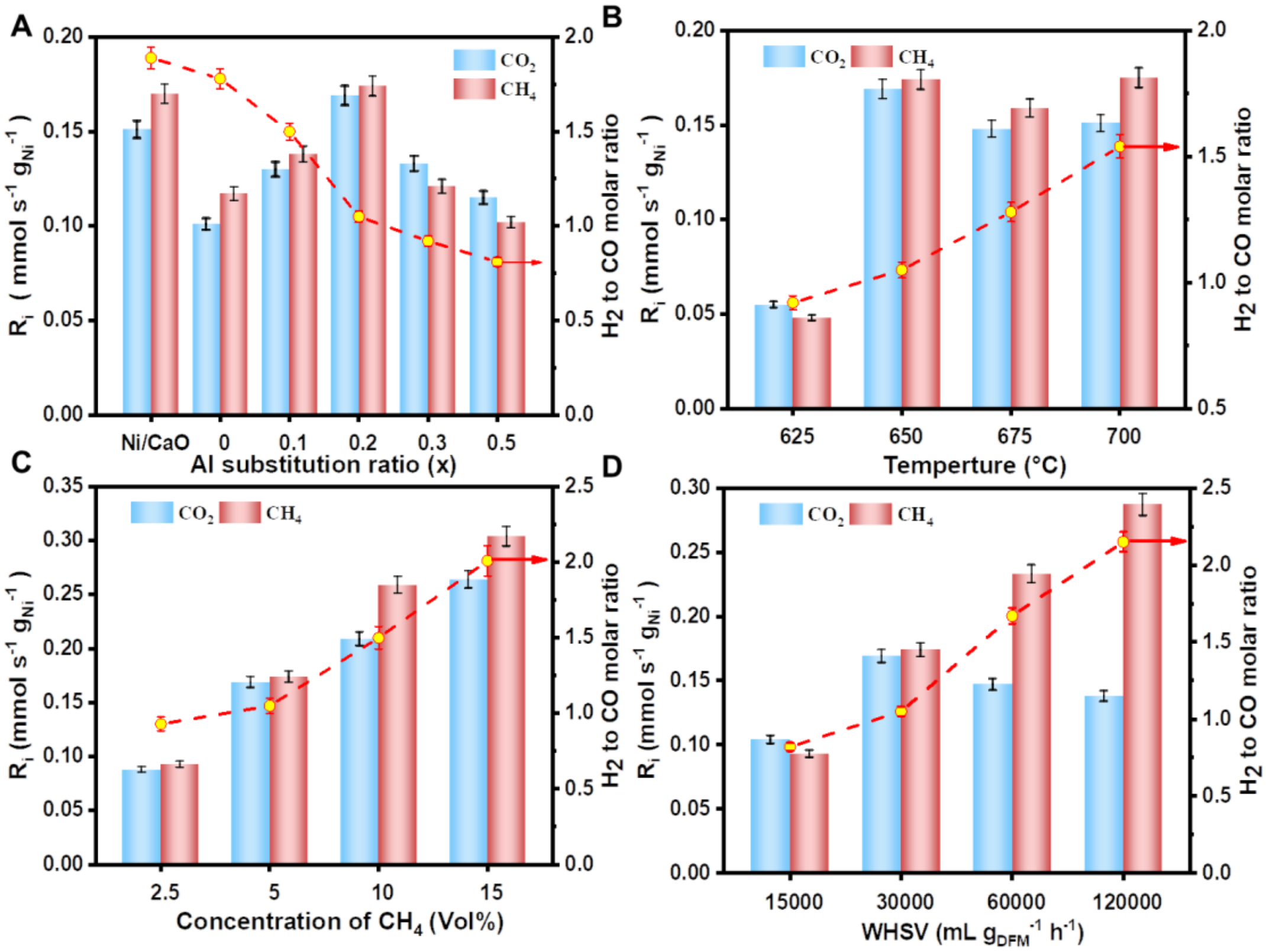

Figure R20 (Figure 4(c, e, g, i)) (a) The effects of Al-doping on average conversion rate and the H_2 to CO molar ratio. Productivity and catalytic performance of iCCC-DRM under (b) different temperatures from 625 to 700°C. The effects of (c) concentration of CH_4 and (d) weight hourly space velocity (WHSV) on the mass activity of the average conversion rate (R_i) of CO_2 and CH_4 along with the H/C molar ratio in the syngas product.

Question 5: *"A major concern associated with the one-pot synthesis approach is the potential incorporation of Ca into the Al-doped $LaNiO_3$ lattice, which may obscure the distinction between the two phases. While the crystal structures of Al-doped $LaNiO_3$ and CaO are distinguishable, the results shown in Figure S2 and Figure 2D suggest that Ca incorporation into the perovskite phase cannot be ruled out. If such incorporation occurs, particularly in a manner dependent on the Al doping level, it could significantly affect the interpretation of the results and the proposed mechanism. Therefore, the authors should provide more rigorous analytical evidence to clearly confirm the phase separation and exclude possible Ca substitution in the perovskite lattice."*

Response: We sincerely appreciate the reviewer's thoughtful and critical comments regarding the potential Ca incorporation into the perovskite phase via the one-pot synthesis route. Admittedly, although the XRD pattern (**Figure S2**), the (HAADF) and energy dispersive spectrometer (EDS) elemental images (**Figure 2D**) in the original manuscript provided clear evidence that the $LaNi_{1-x}Al_xO_3$/CaO DFMs had been successfully produced, it is still difficult to exclude the possibility of Ca doping into the $LaNi_{1-x}Al_xO_3$ perovskite lattice via a one-pot synthesis. Notably, the XRD pattern of the control sample Ni/CaO DFM shows no characteristic diffraction peaks of the $CaNiO_3$ perovskites (**Figure R1**). Accordingly, the readily formed $LaNiO_3$ perovskite under the same synthesis conditions provided a reasonable ground to suspect that $CaNiO_3$ might not exist owing to its less thermodynamically favorable.

Moreover, we adopted the Goldschmidt tolerance factor (*t*, $t = \frac{r_A + r_O}{\sqrt{2}(r_B + r_O)}$) to evaluate the structural stability of $LaNiO_3$ perovskite for the partial substitution of A-site La^{3+} (0.136 nm) by Ca^{2+} (0.134 nm), as well as the partial substitution of B-site Ni^{3+} (0.069 nm) by Al^{3+} (0.054 nm). As illustrated in **Table R1,** Ca-doping results in a slightly step decrease; while Al-doping provides a significant increase from 0.976 to 0.992, further confirming the weak geometric compatibility of Ca incorporation into the perovskite phase, in contrast, Al-doping enhance the structural stability of $LaNiO_3$.

Furthermore, owing to the intrinsic valence charge mismatch caused by the Ca-doping, the inevitable lattice distortion will generate O_v. Such comprehensive effect is positively correlated with those overall contributions as the Al-doping. Considering the substantial CaO in DFMs, the incorporation levels of Ca^{2+} into the ABO_3 perovskite lattice are nearly identical. Accordingly, it is reasonable to regard this as a consistent background baseline when investigating the effects of Al-doping on the catalytic performance of DFMs.

Table R1 Refined lattice parameters of $LaNiO_3$ perovskite via partial substitutions of A-site La^{3+} by Ca^{2+} and B-site Ni^{3+} by Al^{3+}.

	x		t	μ
Ca-doping $La_{1-x}Ca_xNiO_3$	0.5	↑	0.972	0.400
	0.3		0.973	0.400
	0.2		0.974	0.400
	0.1		0.975	0.400
$LaNiO_3$	0		0.976	0.400
Al-doping $LaNi_{1-x}Al_xO_3$	0.1		0.979	0.398
	0.2		0.982	0.396
	0.3		0.985	0.395
	0.5	↓	0.992	0.391

We have supplemented the above crystallographic calculations and discussion with more detailed analysis in the revised manuscript. (see **Page 6, Lines 91-100)**.

"Owing to the selected calcination temperature of 800°C, instead of the formation of $CaAl_2O_4$ (above 1100°C)[43], most Al can partially substitute B site of Ni in $LaNiO_3$ perovskite lattice, which is highly anticipated to suppress the framework collapse of perovskite during the in-situ Ni exsolution. HAADF-STEM image shows that $LaNi_{0.8}Al_{0.2}O_3$ perovskite particles are dispersed in CaO matrix, which will exert a spatial isolation effect to inhibit the sintering of CaO **(Figure S3).** While the EDS mapping images demonstrate the almost overlapped La, Ni, Al elements in $LaNi_{0.8}Al_{0.2}O_3$ perovskites. After the H_2 pretreatment (**Figure S4**), HRTEM images illustrate the well-maintained dispersion state of perovskite particles in $LaNi_{0.8}Al_{0.2}O_3$/CaO DFM (**Figure S5**). From the enlargement, we can see the exsolved Ni NPs (with lattice fringes of 0.203 nm) partially exsolve from the (110) surface (with the lattice of 0.272 nm) of $LaNi_{1-x}Al_xO_3$ perovskite matrix..."

Reviewer #2

"The manuscript presents an interesting concept of a self-adaptive Ni-containing catalyst/adsorbent system for coupled CO_2 capture and methane conversion. The topic is timely and potentially relevant to Nature Communications. However, the novelty claim appears overstated, and the mechanistic interpretation is not yet sufficiently convincing. The authors claim that this type of exsolution-enhanced/sorbent system has not been previously reported. This statement is incorrect. Closely related systems combining Ni-containing perovskites, such as $LaFeNiO_3$–CaO (Small, 10.1002/smll.202401156), have been reported in the literature. The failure to cite and discuss this and similar studies is a serious omission. At a minimum, the authors must explicitly compare their material and performance against these prior reports (as partially done for Ni-based catalysts in Table S6). Without such benchmarking, the manuscript does not meet the standard of novelty expected for this journal."

Response: We deeply appreciate the positive comments from the reviewer and the recognition of the importance of our work. We have carefully studied all comments and made modifications in revised work. Our point-by-point response to the comments be found below.

First of all, we thank the reviewer for raising the concern of overstatement regarding the novelty. Actually, the iCCC-DRM process with the inlet of CO_2 oxidant at the capture stage and switching to the CH_4 reductant at the in-situ conversion stage is of great interesting to study the exsolution-dissolution performance and mechanism of Ni-containing dual functional materials (DFM), although the self-adaptive concept is not new, just as we mentioned in the introduction in the original manuscript. We highly regret that we did not notice this related work about iCCC-DRM, and have supplemented this citation as Ref 37 in the introduction of revised manuscript. After carefully reading this paper (*Small, 10.1002/smll.202401156*), we found they developed a $Ca_2Ni_{0.05}Ti_{0.95}$ DFM, yet few works have focused on the exsolution-dissolution performance and mechanism during the cyclic iCCC-DRM process, which constitutes the core novelty of our present work. The detailed explicit comparison can be found in the following response of Q1.

Question 1: *"The manuscript states that the material/system has not been reported before, but the literature contains closely related examples that should be cited and compared directly. The absence of this discussion weakens the novelty claim and gives the impression that the authors did not sufficiently benchmark the work against prior*

studies."

Response: We thank the reviewer for these valuable suggestions again. We have looked thoroughly of this paper (*Small, 10.1002/smll.202401156*), which reported a $Ca_2Ni_{0.05}Ti_{0.95}$ multifunctional material (MFM) composed of Ni, $CaTiO_3$ and CaO for the integrated CO_2 capture and dry reforming of methane at 700°C. Where TiO_2 was doped into CaO to promote its stability. However, the generation of $CaTiO_3$ consumed CaO, resulting in a significantly decreased CO_2 adsorption capacity of 6.49 mmol g_{DFM}^{-1}. The Ni catalyst was then loaded on $CaTiO_3$/CaO adsorbent, which was a common approach to fabricate DFM. Although the $CaTiO_3$ may help the dispersion of Ni in part, the post-impregnation usually made the size of Ni nanoparticles unevenly, providing extremely low CO_2 and CH_4 conversion efficiency of 43.0% and 4.0%, respectively, during the conversion stage. In addition, related studies reported perovskite ($CeCoO_3$, $LaFeO_3$) as structural stabilization to prevent the CaO sintering and improve the cyclic stability. Therefore, the perovskites were merely utilized for improving the CO_2 adsorption performance, rather than for optimizing the dispersion of Ni sites, consequently, the reaction efficiency of CH_4 and CO_2 usually remained unsatisfactory (**Table S5**).

While in this study, through a convenient one-pot synthesis, the perovskite-based $LaNi_{1-x}Al_xO_3$/CaO dual-functional materials (DFMs) were developed. The rational partial-substitution of Al at the B-site of $LaNi_{1-x}Al_xO_3$ triggered the Jahn-Teller distortion and lattice parameters shrinkage, making Ni^{2+}/Ni^{3+} ratio elevated and oxygen vacancies enriched. Notably, the stable Al-doped perovskite structure enabled the reversible exsolution and dissolution of Ni nanoparticles during the redox iCCC-DRM process. Accordingly, the CO_2 adsorption capacity, catalytic activity and stability were simultaneously enhanced following the Sabatier principle. After the optimization, $LaNi_{0.8}Al_{0.2}O_3$/CaO DFM effectively suppresses Ni sintering and carbon deposition, delivering superior conversion efficiencies of CO_2 and CH_4, as high as 91.5% and 93.5%, respectively, at high CO_2 capture capacity of 10.1 mmol g_{DFM}^{-1}, achieving excellent cyclic stability in iCCC-DRM.

Distinct structure-performance comparisons demonstrate notable differences in the two researches. Superior DFMs can only be achieved via rational design and elaborate regulation, serving as a key material innovation of this manuscript.

More importantly, as the core novelty of this study, the intelligent exsolution-dissolution mechanism based on MSI tuning stands out to achieve exceptional synergistic performance by taking the cyclic redox regimes of iCCC-DRM as the target system. Specifically, we have successfully bridged the knowledge gap between the MSI principle and iCCC performance by unraveling two core fundamental questions:

(1) The dynamic evolution mechanism of adsorption and catalytic active sites in DFMs through the intrinsic redox steps of the iCCC process was revealed.

(2) Rational modulation of MSI balance catalytic activity and structural stability to realize well-matched CO_2 adsorption and in-situ conversion, thus achieving durable iCCC against sintering.

Accordingly, we sincerely hope that the reviewer can fully recognize the contributions and progressive advances of this study. As suggested by the reviewer, we have added the relevant references and the comparisons have been summarized in **Table S5** in the revised Supporting Information, together with the corresponding discussions in Introduction of the revised manuscript.

(Page 4, Lines 60-69)"…Spatial isolation strategies have been predominantly achieved by doping high Tamman temperature oxides or perovskite (e.g., MgO, Al_2O_3, CeO_2, ZrO_2, TiO_2, $CaTiO_3$, $LaFeO_3$) [36–39] to prevent the coalescence of CaO through the physical confinement effect [40,41]. Nevertheless, achieving simultaneous enhancement of the CO_2 adsorption capacity and conversion efficiency remains challenging in iCCC-DRM. Although some approaches have been developed to simultaneously promote DFMs' stability and reducibility[42], the promising MSI regulation strategy remains far from well-studied. Specifically, a comprehensive understanding of the reversible exsolution-dissolution mechanism of catalytic sites in perovskites phase and the cooperation between active metal sites and adsorptive sites during iCCC represents a significant knowledge gap in rational design of DFMs."

(Page 16, Lines 259-263) "… Such stability of $LaNi_{0.8}Al_{0.2}$/CaO can be attributed to the perovskite's high Tamman temperature (~800°C) and its spatial confinement effect for separating CaO domains, which collectively prevent sintering and agglomeration during the redox iCCC-DRM process. More importantly, perfectly stable CO_2 and CH_4 conversion efficiencies of $LaNi_{0.8}Al_{0.2}O_3$/CaO are observed as high as 91.5% and 93.5% without any decline at 650 °C (**Table S5**)…"

Table S5 The numerical comparison of iCCC-DRM processes and reported conventional DRM process in the literature.

Catalyst	Gas composition	WHSV	Temperature (°C)	CH_4 conversion (%)	CO_2 conversion (%)	H_2/CO molar ratio ($R_{H/C}$)	Ref.
$LaNi_{0.8}Al_{0.2}O_3/CaO$	**Capture step:** 10 vol.% CO_2/N_2; **Conversion step:** 5vol.% CH_4/N_2	30 L g^{-1} h^{-1} (50 ml/min)	650	93.5	91.5	1.05	**This work**
$Ca_2Ni_{0.05}Ti_{0.95}$	**Capture step:** 10 vol.% CO_2/N_2; **Conversion step:** 5vol.% CH_4/N_2	60 L g^{-1} h^{-1} (50 mL/min)	700	4.0	43.0	0.73	1
$Ce_{0.75}Co_{0.25}NiO_3/Ca$	**Capture step:** 10 vol.% CO_2/Ar; **Conversion step:** 5vol.% CH_4/Ar	3 L g^{-1} h^{-1} (25 mL/min)	700	94.0	77.0	2.30	2
$5Ni\text{-}30CaO\text{-}LaFeO_3$	**Capture step:** 15vol.% CO_2/Ar; **Conversion step:** 5vol.% CH_4/Ar	10 L g^{-1} h^{-1} (50 mL/min)	700	74.8	86.5	1.0	3
$Ni/CaO\text{-}Al_2O_3$	**Capture step:** 15vol.% CO_2/N_2; **Conversion step:** 3vol.% CH_4/N_2	60 L g^{-1} h^{-1} (100mL/min)	650	74.7	83.6	0.96	4
Ni-CaO catal-sorbent	**Capture step:** 10 vol.% CO_2/N_2; **Conversion step:** 10vol.% CH_4/N_2	12 L g^{-1} h^{-1} (40 ml/min)	700	/	83.8	6.53	5
Ni-Ca@Zr	**Capture step:** 5 vol.% CO_2/N_2; **Conversion step:** 8 vol.% CH_4/N_2	36 L g^{-1} h^{-1} (30 ml/min)	720	40	45	0.8	6

Ni/NiCa (2.6 wt%)	**Capture step:**<br>10% CO2/Ar,<br>**Conversion step:**<br>10%CH4/Ar,	20 mL/min	650	67	78	0.9	7
Ni-Ca_{10}Mg	**Capture step:**<br>20% CO_2/N_2<br>**Conversion step:**<br>10%CH_4/N_2,	200 mL/min	720	90	60	0.92	8
$NiCa_{1.6}K_{0.4}$@Al_2O_3	**Capture step:**<br>20 % CO_2/Ar<br>**Conversion step:**<br>10%CH4/Ar,	50ml/min	750	25	52	2.1	9
Ni/SiO_2-E	$CH_4/CO_2/N_2$ = 3:3:4	30 L g^{-1} h^{-1}	500	7.9	17.9	0.5	10
0.2Ce-$La_{0.97}Ni_{0.4}Cr_{0.6}O_3$	CH_4: CO_2: N_2 = 1: 1: 1	30 L g^{-1} h^{-1}	800	87.4	92.9	0.93	11
10NiOMgO-PACS	CH_4/CO_2/He=1:1:3	72 L g^{-1} h^{-1}	700	78.5	93.7	0.85	12
NiFe/Al_2O_3	CH_4/CO_2 = 1:1	12 L g^{-1} h^{-1}	550	26.6	37.8	0.67	13
$MgNiO_2$@Ni@$MgNiO_2$	CH_4: CO_2 = 1:1	70 L g^{-1} h^{-1}	700	68	78	0.9	14
Ni-CaO/MCM-41	CH_4/CO_2/Ar = 1:1:1	143 L g^{-1} h^{-1}	650	20	40	0.5	15
15%Ni@S-1	CH_4/CO_2 = 1:1	100 L g^{-1} h^{-1}	650	40	42	0.6	16
$Ni_3Fe_1Cu_1$-MA	$CH_4/CO_2/N_2$ = 2:2:1	432 L g^{-1} h^{-1}	650	15	35	0.5	17
Ni/MCF	CH_4/CO_2/Ar = 9:9:2	36 L g^{-1} h^{-1}	650	60	70	1.0	18
Ni/MgO	CH_4/CO_2/Ar = 1:1:8	30 L g^{-1} h^{-1}	700	≥37	≥50	0.78	19
0.8MgO^{WI}/Ni@NiAlO	CH_4/CO_2/Ar=1:1:3	60 L g^{-1} h^{-1}	600	50	61	/	20
NiMo/MgO	CH_4/CO_2/He = 1:1:8	60 L·g^{-1} h^{-1}	800	100	100	1	21

Question 2: *"The proposed theory of oxygen-vacancy diffusion from the perovskite phase to CaO is not convincing as presently written. This mechanism needs stronger experimental or computational support, or the authors should revise the interpretation to a more conservative and defensible explanation."*

Response: We thank the reviewer for this valuable suggestion. Following the reviewer's suggestion (as well as the similar one from the Reviewer 1), we have added more experimental results to elucidate the CO_2 adsorption mechanism over $LaNi_{1-x}Al_xO_3$/CaO DFM.

As reported, the actual carbonation is a complex three-step process including ① surface adsorption of CO_2, ② reaction with O^{2-} to generate CO_3^{2-}, and ③ ion exchange with CaO to form $CaCO_3$. Accordingly, the O^{2-} specie plays a significant bridge role in the CO_2 adsorption kinetics. Naturally, to keep electrical neutrality, O^{2-} species will migrate outward, in the opposite direction of inward bulk diffusion of CO_3^{2-} to accommodate CO_2 molecules (**Figure R10**). However, such O^{2-} migration proceeds sluggishly owing to lack of additional driving force, resulting in the serious retarded CO_2 diffusion caused by the in-situ formed dense $CaCO_3$ layer over CaO. It has been reported that oxygen vacancies (O_v) can facilitate the CO_2 adsorption by the insertion, thus providing the active O^{2-} species (*Ind. Eng. Chem. Res., 2022, 61: 5527-5535*). This can accelerate the O^{2-} migration through the $CaCO_3$ product layer via the O_v hopping mechanism (*Chem Catal., 2023, 3: 100785*). Accordingly, introducing O_v in CaO-based adsorbent can serve as a promising strategy to address the challenge of sluggish CO_2 diffusion through the $CaCO_3$ layer.

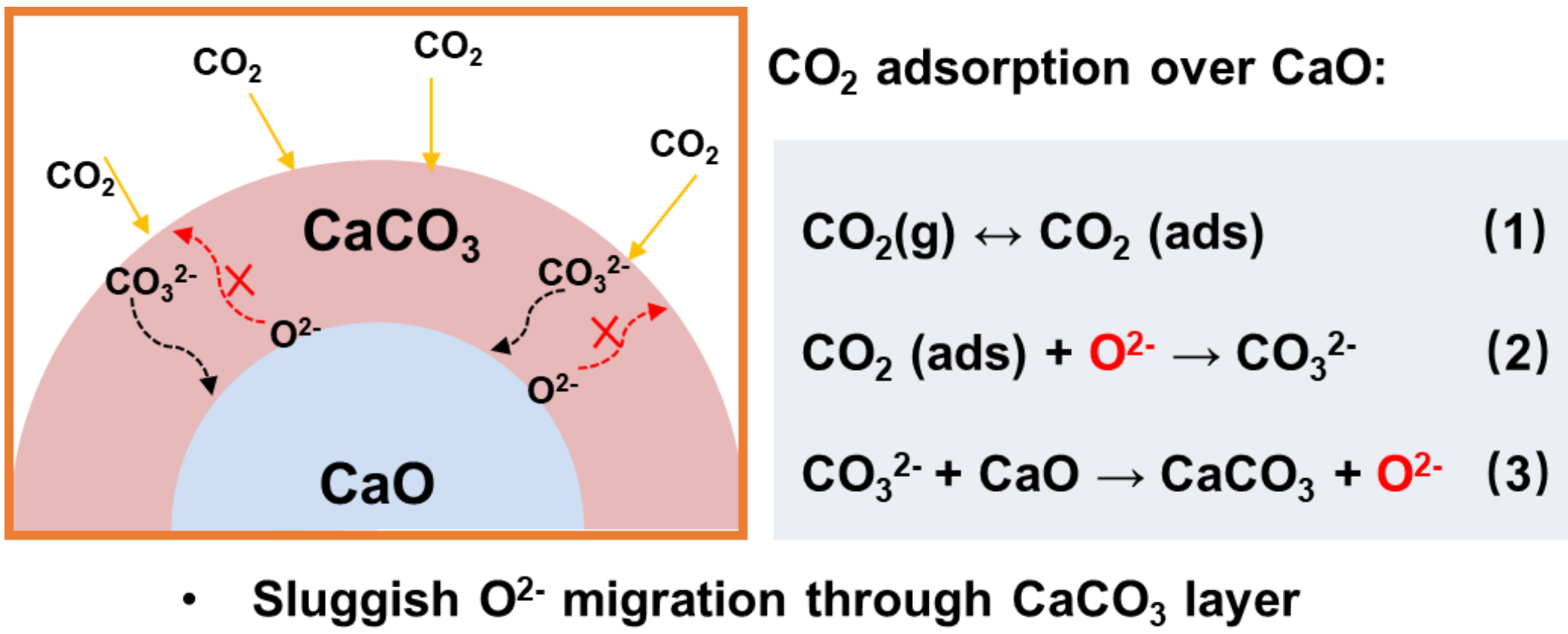

Figure R10 (Figure 5A) CO_2 adsorption mechanism of CaO-based adsorbents.

Notably, ABO_3 perovskite is a typical intrinsic oxygen-deficient crystalline, especially when the B site is partially substituted by another metal. The metal doping will induce the open BO_6 octahedral framework more distorted, which can be assessed

by the octahedron factor ($\mu=r_B/r_O$), the ratio of average radius of B cations over that of O anion. With increasing the amount of Al substitution, the μ of $LaNi_{1-x}Al_xO_3$ perovskite decreases from 0.400 to 0.391 (**Table S4**), revealing more lattice distortion and hence more O_Vs for facilitating O^{2-} migration. Therefore, we highly anticipate through one-pot sol-gel method, the $LaNi_{1-x}Al_xO_3/CaO$ DFMs can produce abundant O^{2-} migration channels.

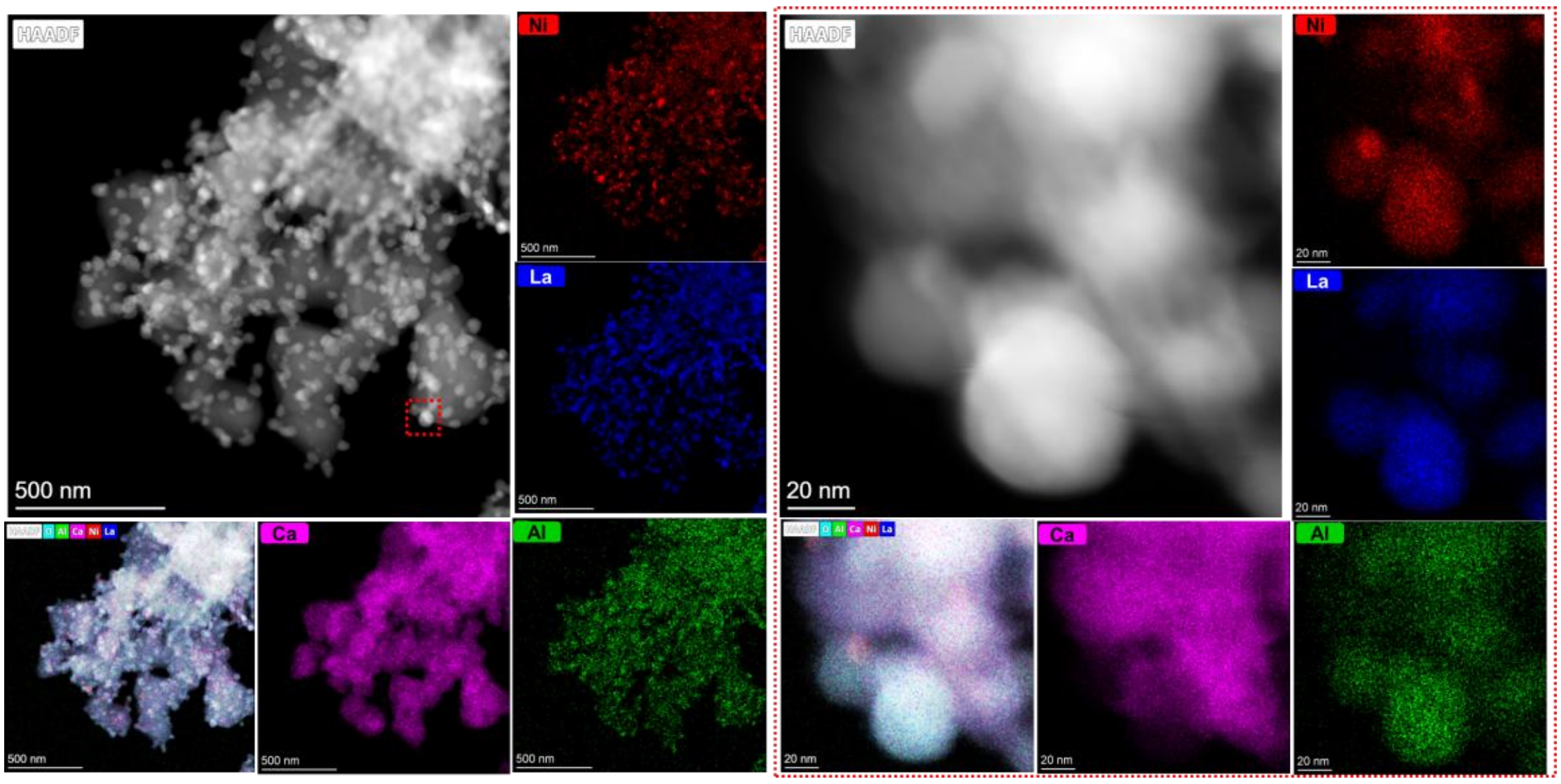

Figure R11(Figure S3) STEM-HAADF and EDS elemental mapping images of the as-synthesized $LaNi_{0.8}Al_{0.2}O_3/CaO$ DFM.

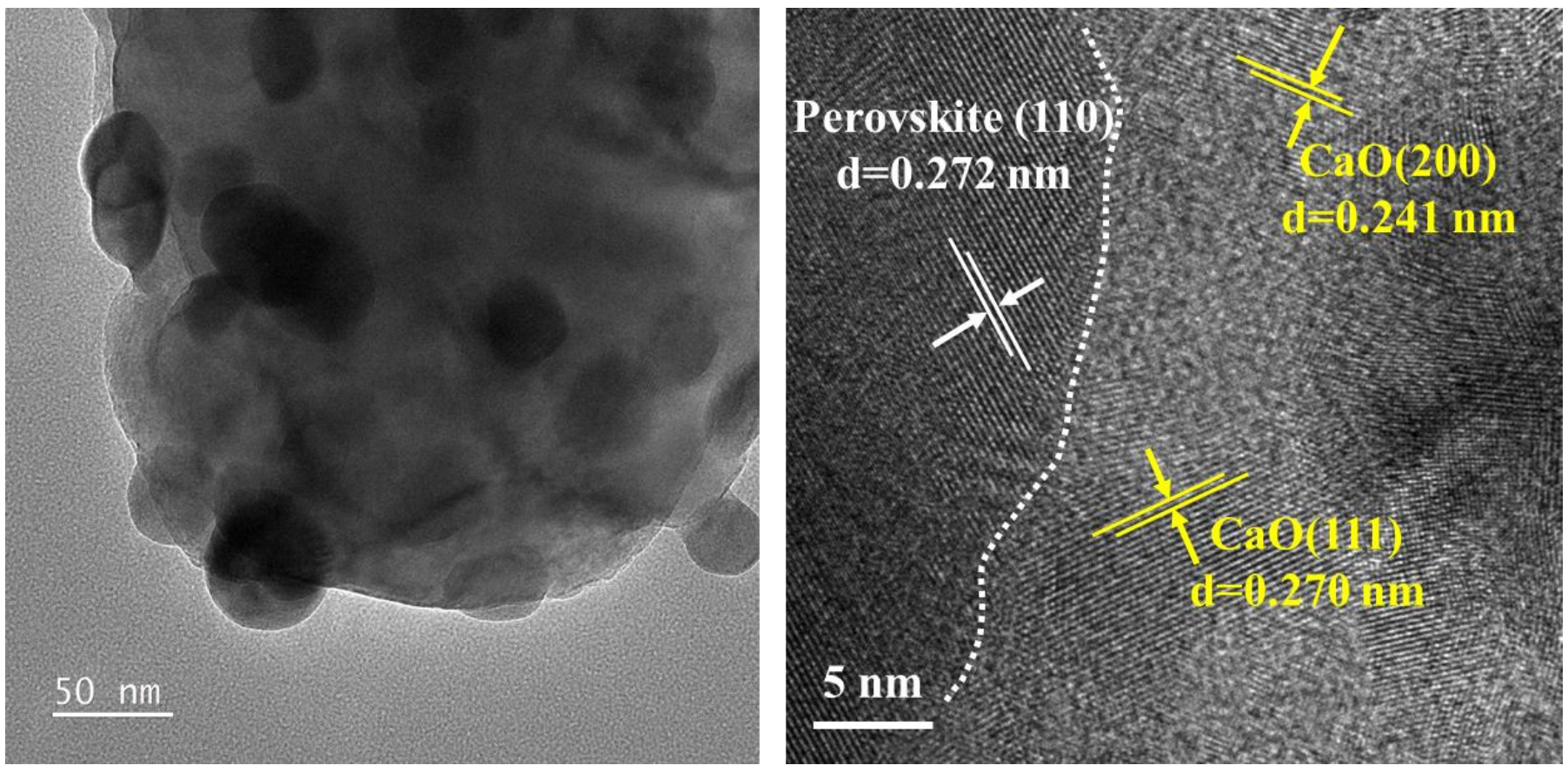

Figure R12(Figure S21) HRTEM images of as-synthesized $LaNi_{0.8}Al_{0.2}O_3/CaO$ DFM with intimately contacted heterogeneous interfaces.

The STEM-HAADF and EDS elemental mapping images in different scales all verify the well dispersed $LaNi_{1-x}Al_xO_3$ perovskite particles in CaO matrix (**Figure R11**). Meanwhile, the intimately contacted heterogeneous interfaces between $LaNi_{1-x}Al_xO_3$ perovskite and CaO are further verified by HRTEM image (**Figure R12**). The electron paramagnetic resonance (EPR) spectra of $LaNi_{0.5}Al_{0.5}O_3/CaO$ further exhibits the

strongest signal response at g = 2.004, positively correlated with the concentrated O_vs (**Figure R13**). Accordingly, the CO_2 adsorption mechanism in $LaNi_{1-x}Al_xO_3$/CaO DFM is demonstrated. As illustrated in **Figure R14**, the abundant O_vs original from $LaNi_{1-x}Al_xO_3$ can act as the driving force to induce reactive O^{2-} migration: that O^{2-} migrates to adjacent O_v, leaving a new O_v at the original lattice site and subsequently reoccupied by surrounding O^{2-}. In this way, O_vs are dynamically occupied and regenerated, providing O^{2-} transport channels for continuously accelerating CO_2 diffuse across the interface and react with inner CaO to sustain excellent CO_2 capture performance.

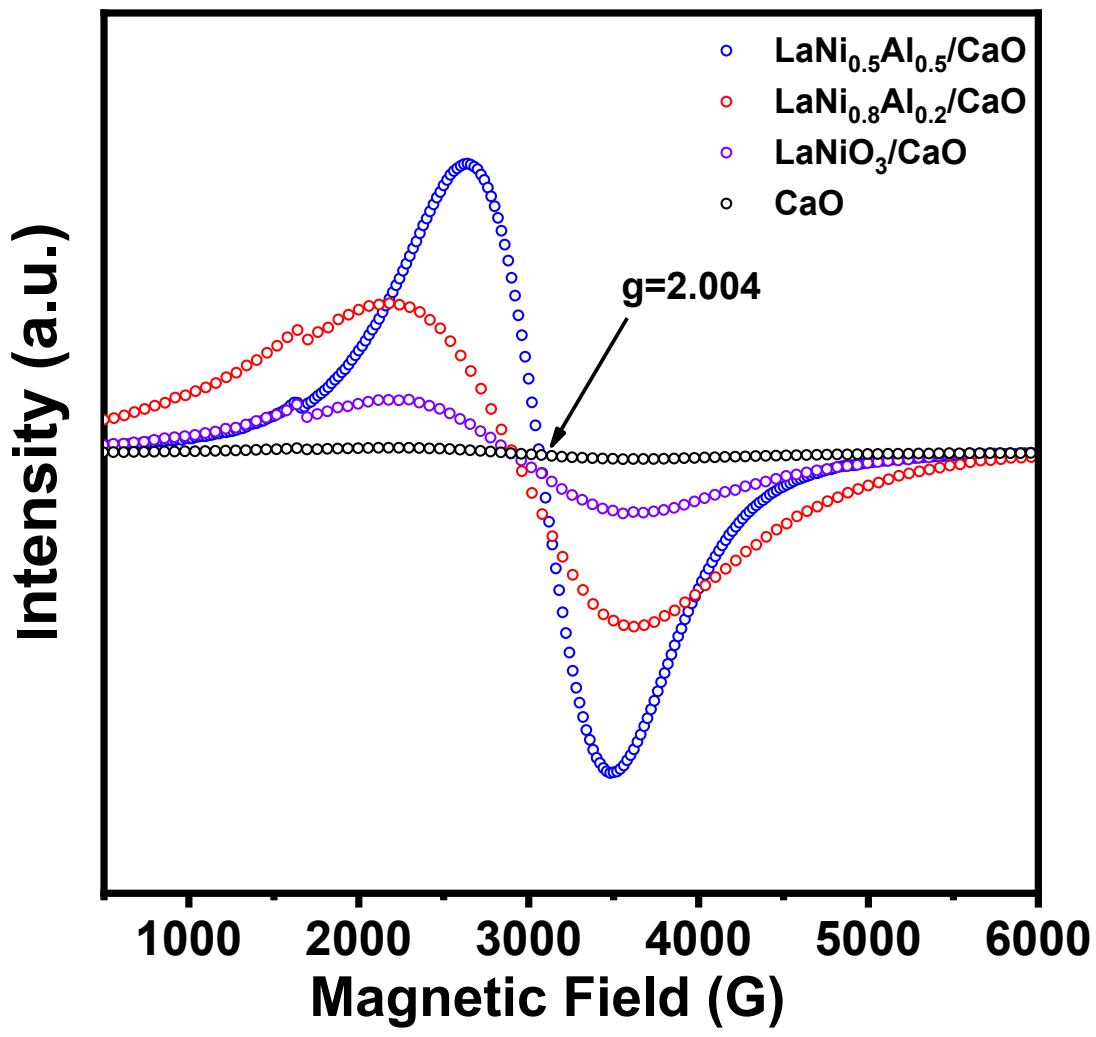

Figure R13 (Figure S9) EPR curves of CaO sorbent and $LaNi_{1-x}Al_xO_3$/CaO DFMs.

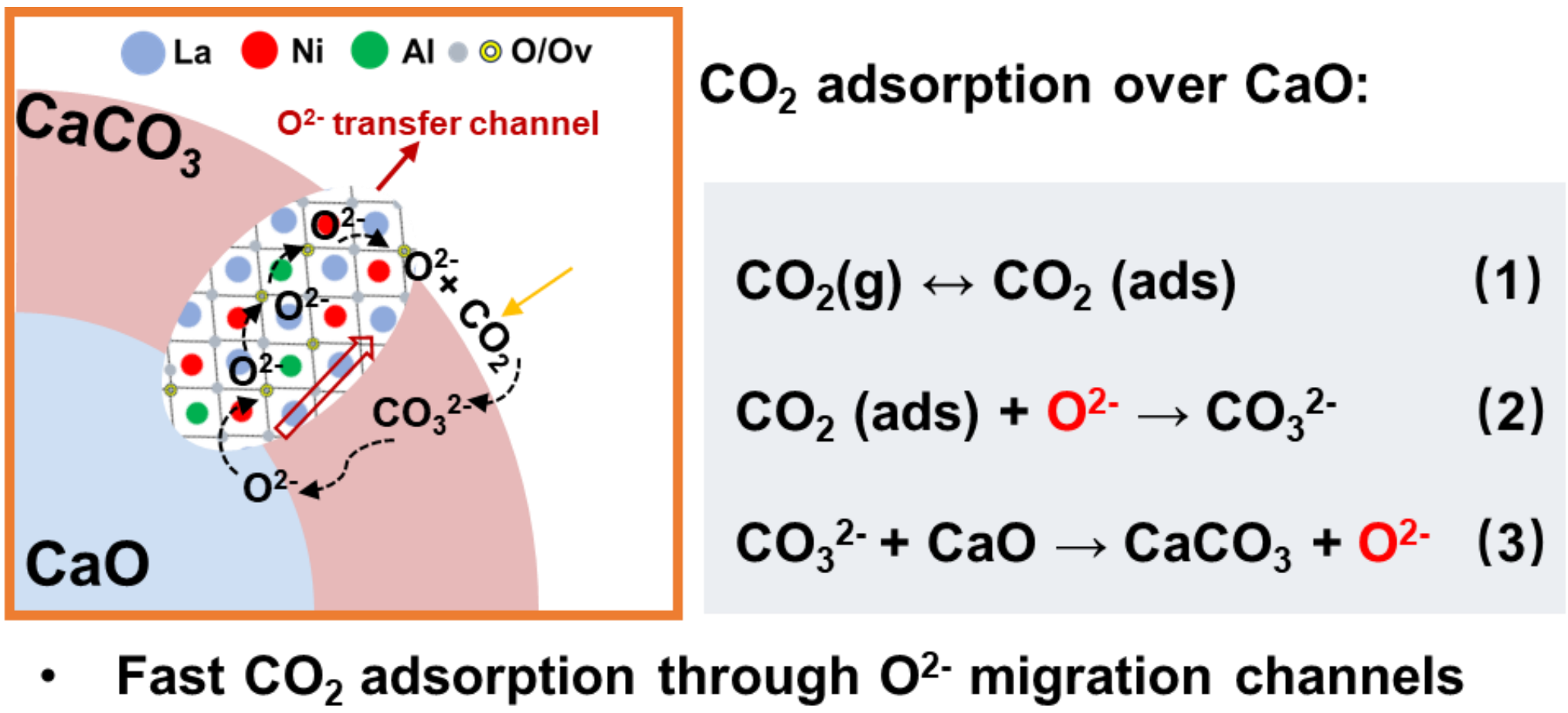

Figure R14 (Figure 5A) Schematic diagrams of the role of O^{2-} migration channels in DFMs for CO_2 adsorption.

As expected, the CO_2 adsorption rate increases with incorporation of Al-doping perovskites (**Figure R15a**). Each adsorption kinetic curves at 650 ℃ was well simulated by the double exponential model (**Table S5**). Notably, the initial fast chemical

adsorption (carbonation) rate constant (k_1) and subsequent CO_2 diffusion-controlled rate constant (k_2) of $LaNi_{0.5}Al_{0.5}O_3$/CaO (4.17 min^{-1}, 0.25 min^{-1}) are 3-6 times larger than those of CaO (0.72min^{-1}, 0.09min^{-1}), respectively. Importantly, we have identified a remarkable linear relationship between k_1(k_2) and the intensity of O_v EPR signal, verifying the concentration of O_v directly governs the O^{2-} migration barrier and accelerate CO_2 diffusion (**Figure R15b**).

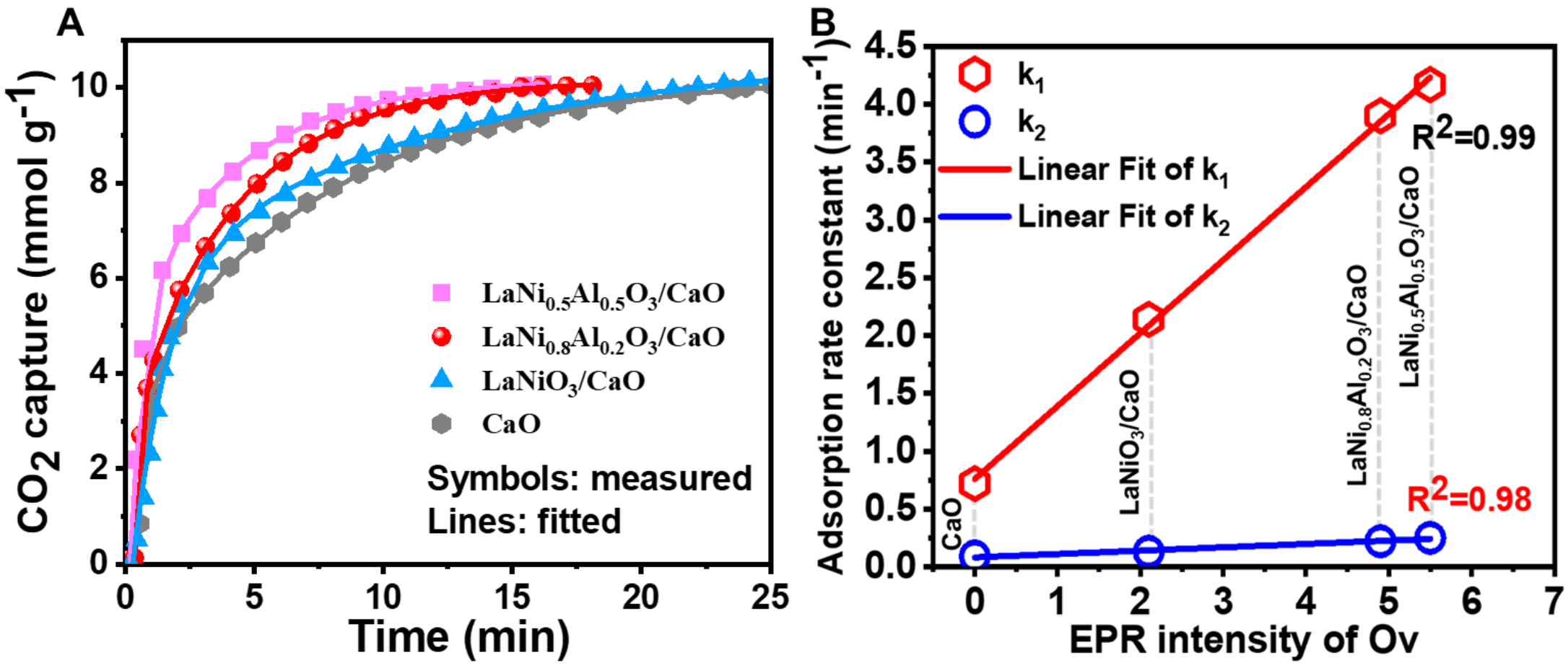

Figure R15 (Figure S22 and Figure 5C) (a) Experimental and kinetic fittings of the CO_2 adsorption on fresh CaO sorbent and $LaNi_{1-x}Al_xO_3$/CaO DFMs. (Test conditions: 650 °C, 10vol% CO_2 /2vol%O_2 /N_2). (b) the regression fitting between k_1(k_2) and the intensity of O_v EPR signal of CaO and $LaNi_{1-x}Al_xO_3$/CaO DFMs.

Therefore, we can conclude $LaNi_{1-x}Al_xO_3$ perovskite phase in DFMs boosts the CO_2 adsorption kinetics through providing abundant O^{2-} migration channels in CaO matrix. What's more, it also plays a synergistic role to enhance CO_2 adsorption stability through restraining the agglomeration of CaO grains by intimate interfacial contact.

Table S6 Kinetic parameters of CO_2 adsorption on CaO sorbent and Al-doped $LaNiO_3$/CaO DFMs at 650°C.

Samples	A1	A2	A3	k_1 / min^{-1}	k_2 / min^{-1}	R^2
CaO	10.68	-7.36	-4.78	0.72	0.09	0.997
$LaNiO_3$/CaO	10.27	-7.13	-6.77	2.14	0.13	0.999
$LaNi_{0.8}Al_{0.2}O_3$/CaO	10.15	-12.72	-7.40	3.90	0.22	0.999
$LaNi_{0.5}Al_{0.5}O_3$/CaO	10.17	-13.28	-6.89	4.17	0.25	0.999

Accordingly, in the revised manuscript, we have revised the original Figure 5A and added Figures R10 and R14, R11, R12, R13, and R15 as Figures 5A, S3, S21, S9, S22 and Figure 5D, and elucidated the oxygen vacancies contributions to the enhanced CO_2 adsorption on $LaNi_{1-x}Al_xO_3$/CaO DFM as the following.

(Page 17, Lines 284-299) "The conventional carbonation consists of three steps ① surface adsorption of CO_2, ② reaction with O^{2-} to generate CO_3^{2-}, and ③ ion exchange with CaO to form $CaCO_3$. Naturally, O^{2-} species will migrate outward to accommodate CO_2 molecules. However, such O^{2-} migration proceeds sluggishly owing to lack of driving force, resulting in the serious retarded CO_2 diffusion caused by the in-situ formed dense $CaCO_3$ layer over CaO (**Figure 5A**). It has been reported that oxygen vacancies (O_v) can facilitate the CO_2 adsorption via the O_v hopping mechanism [53–55], which accelerates the O^{2-} migration through the $CaCO_3$ product layer. Accordingly, introducing O_v in CaO-based adsorbent can serve as a promising strategy to address the challenge of sluggish CO_2 diffusion through the $CaCO_3$ layer.

Notably, with increasing the amount of Al substitution, the octahedron factor (μ) of $LaNi_{1-x}Al_xO_3$ perovskite in DFMs decreases from 0.400 to 0.391 (**Table S4**), revealing more lattice distortion and hence more O_vs. Thanks to the intimately contacted between $LaNi_{1-x}Al_xO_3$ perovskite and CaO (**Figure S21**), the abundant O_vs generated in $LaNi_{1-x}Al_xO_3$ can act as the driving force to induce reactive O^{2-} migration. Specifically, O^{2-} migrates to adjacent O_v, leaving a new O_v at the original lattice site and subsequently reoccupied by surrounding O^{2-}. In this way, O_vs are dynamically occupied and regenerated, providing O^{2-} transport channels for CO_2 to diffuse across the $CaCO_3$ layer and react with inner CaO."

(Page 18, Lines 305-313) "This enhanced CO_2 adsorption kinetic was illustrated by the double exponential model (**Eq. S1,** see in the supplementary information), that at 650 °C, the initial fast chemical adsorption (carbonation) rate constant (k_1) and subsequent CO_2 diffusion-controlled rate constant (k_2) of $LaNi_{0.5}Al_{0.5}O_3$/CaO (4.17 min^{-1}, 0.25 min^{-1}) are 3-6 times larger than those of CaO (0.72min^{-1}, 0.09min^{-1}), respectively (**Figure S22** and **Table S6**). Importantly, we have identified a remarkable linear relationship between k_1, or k_2 and the intensity of O_v EPR signal (**Figure 5C**) verifying the concentration of O_v directly governs the O^{2-} migration barrier and accelerate CO_2 diffusion. Therefore, $LaNi_{1-x}Al_xO_3$ perovskite phase in DFMs boosts the CO_2 adsorption kinetics through providing abundant O^{2-} migration channels in CaO matrix."

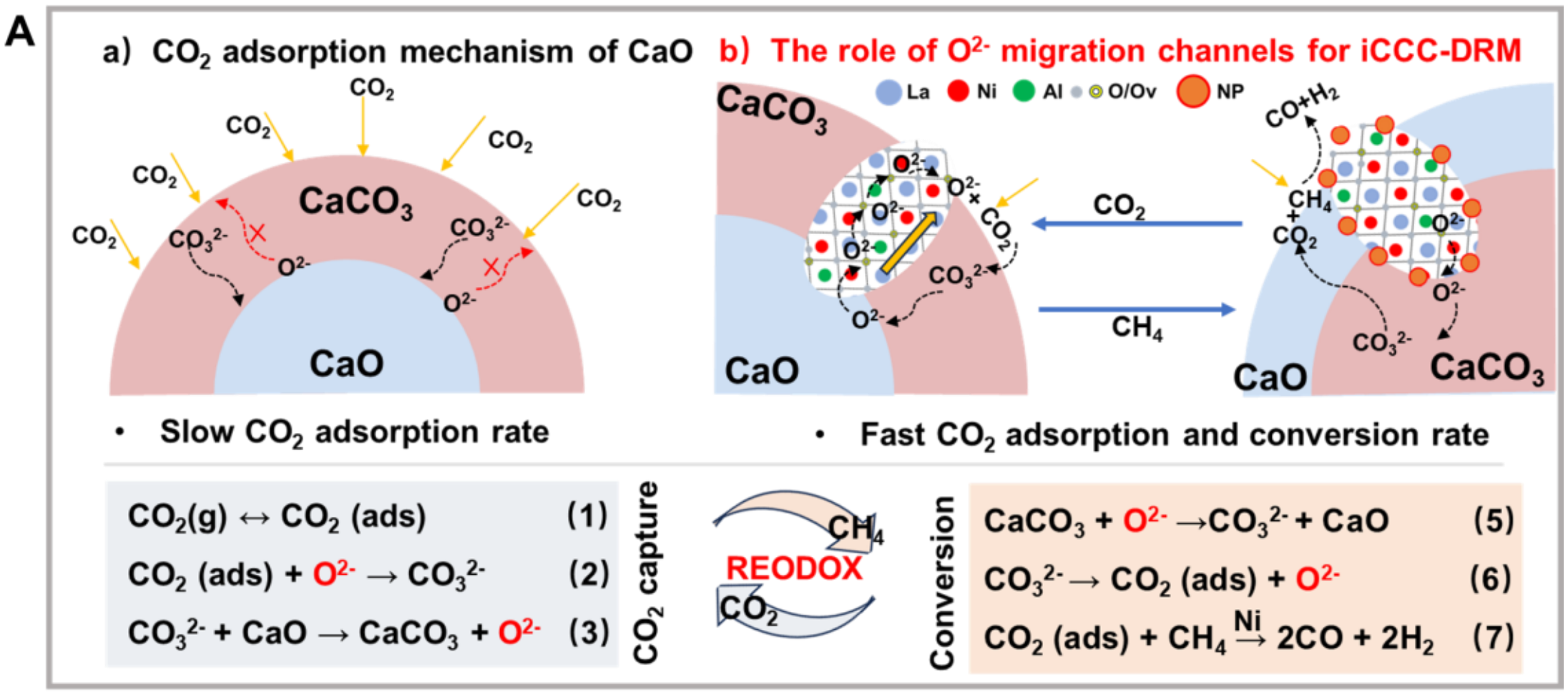

Figure 5A. Schematic diagrams of the role of O^{2-} migration channels in DFMs for iCCC-DRM.

"Question 3: *The manuscript would benefit from additional imaging and interfacial characterization to clarify how the perovskite is connected to the CaO grains. Larger-scale STEM-EDS imaging would help establish whether the interface architecture is truly responsible for the observed cycling behavior."*

Response: We thank the reviewer for this valuable suggestion. As illustrated in the response for Q1, the STEM-HAADF and EDS elemental mapping images in different scales all verify the well dispersed $LaNi_{1-x}Al_xO_3$ perovskite particles in CaO matrix (**Figure R22**). Meanwhile, the intimately contacted heterogeneous interfaces between $LaNi_{1-x}Al_xO_3$ perovskite and CaO are further verified by HRTEM image (**Figure R23**).

Moreover, as suggested, we have supplemented large-scale (200 nm) STEM-HADDF and EDS mapping images of the optimized $LaNi_{0.8}Al_{0.2}O_3$/CaO DFM during the cycling iCCC-DRM process. At both CO_2 capture and DRM conversion stages, the STEM-EDS images show that the $CaCO_3$(or CaO) and $LaNi_{1-x}Al_xO_3$ perovskite are strongly coupled with each other, successfully preventing CaO sintering during volumetric expansion. Notably, The EDS signals of La, Ni and Al are highly overlapped, suggesting that Ni nanoparticles are incorporated into the $LaNiO_3$ perovskite. However, the images in large-scale can hardly tell the exact dissolution-exsolution features of Ni NPs. Therefore, we supplied the images in the revised supporting information to clarify the overall morphology how the perovskite is connected to the CaO grains.

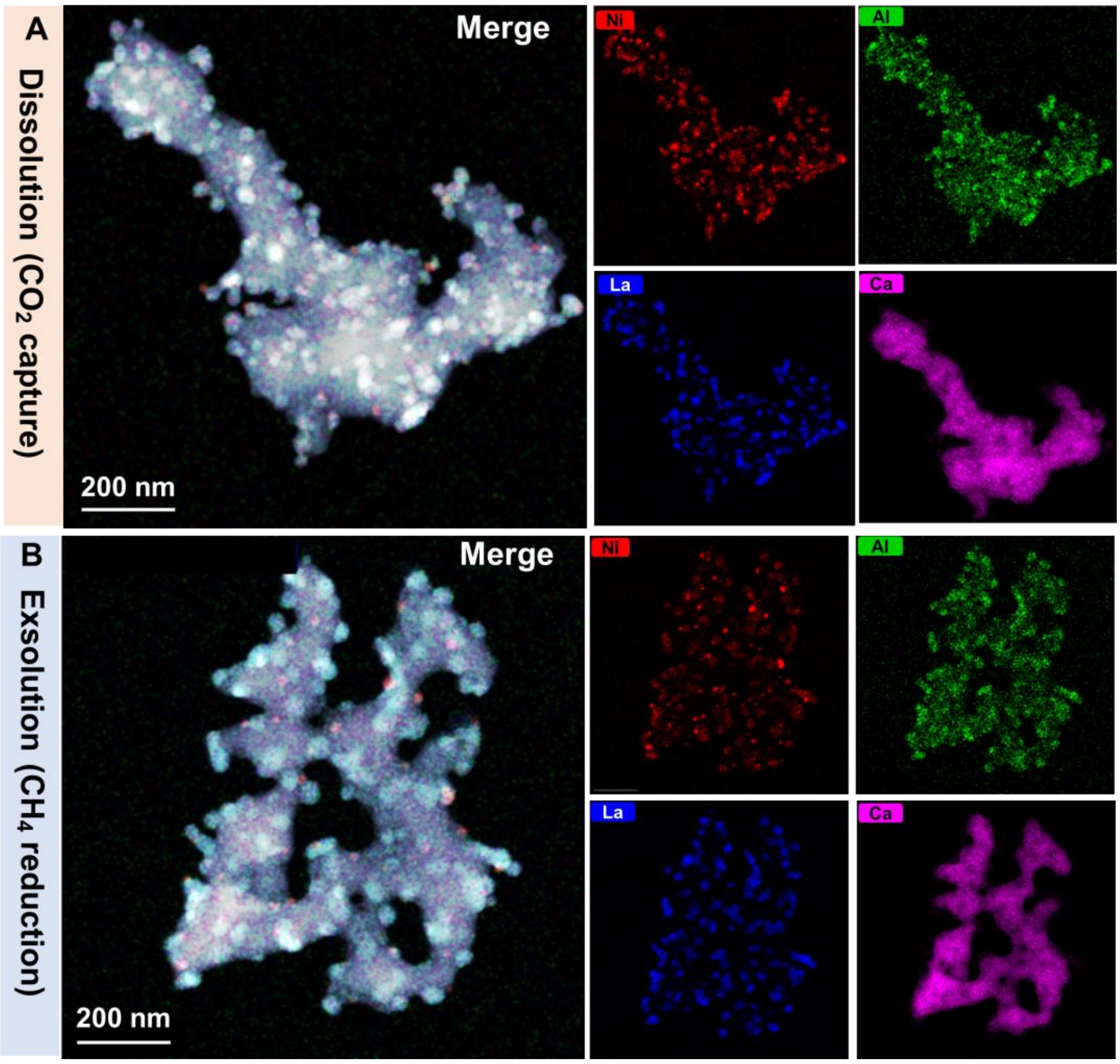

Figure R27 (Figures S7-S8) TEM-HADDF images of $LaNi_{0.8}Al_{0.2}O_3$/CaO DFM at the stages of (a) CO_2 adsorption and (b) subsequently DRM conversion by CH_4. Scale bar: 200 nm.

To demonstrate whether the interface architecture is truly responsible for the observed cycling behavior, the high-resolution (20 nm) STEM-HADDF and EDS mapping images (**Figure R27**) and HRTEM images of $LaNi_{0.8}Al_{0.2}O_3$/CaO DFM during the iCCC-DRM (**Figure R28**) have been supplied. Specifically, in the STEM images of reduced $LaNi_{0.8}Al_{0.2}O_3$/CaO after CO_2 capture condition (10vol%/2vol%O_2/N_2), the exsolved Ni nanoparticles were re-incorporated into the subsurface of $LaNi_{1-x}Al_xO_3$. However, some larger Ni NPs still existed in the perovskite due to the kinetic of re-dissolution. After CH_4 reduction, the STEM-HADDF images illustrated that the partial Ni of $LaNi_{0.8}Al_{0.2}O_3$/CaO was reduced and exsolved, and the exsolved Ni NPs were embedded in the host $LaNi_{1-x}Al_xO_3$ perovskite. It is expected that the in-situ exsolved Ni NPs formed from bulk-to-surface have a stronger interaction with $LaNi_{1-x}Al_xO_3$, which is truly responsible for maintaining small and dispersed Ni NPs on the

surface of CaO. HRTEM further confirmed the coexistence of three phases of Ni NPs, $LaNi_{1-x}Al_xO_3$ perovskite and CaO, resolving the distinct lattice fringes of 0.203, 0.272, and 0.240 nm, respectively (**Figure R29**). Therefore, it is concluded that the exsolved Ni NPs from the stable $LaNi_{1-x}Al_xO_3$ structure can be resolution after CO_2 capture (oxidative condition), and then exsolved after CH_4 reduction (reductive condition). Thus, such stability of $LaNi_{0.8}Al_{0.2}$/CaO can be attributed to the perovskite's high Tamman temperature (~800°C), and the spatial confinement of CaO domains by coherent lattice, which collectively prevent sintering and agglomeration during the redox iCCC-DRM process.

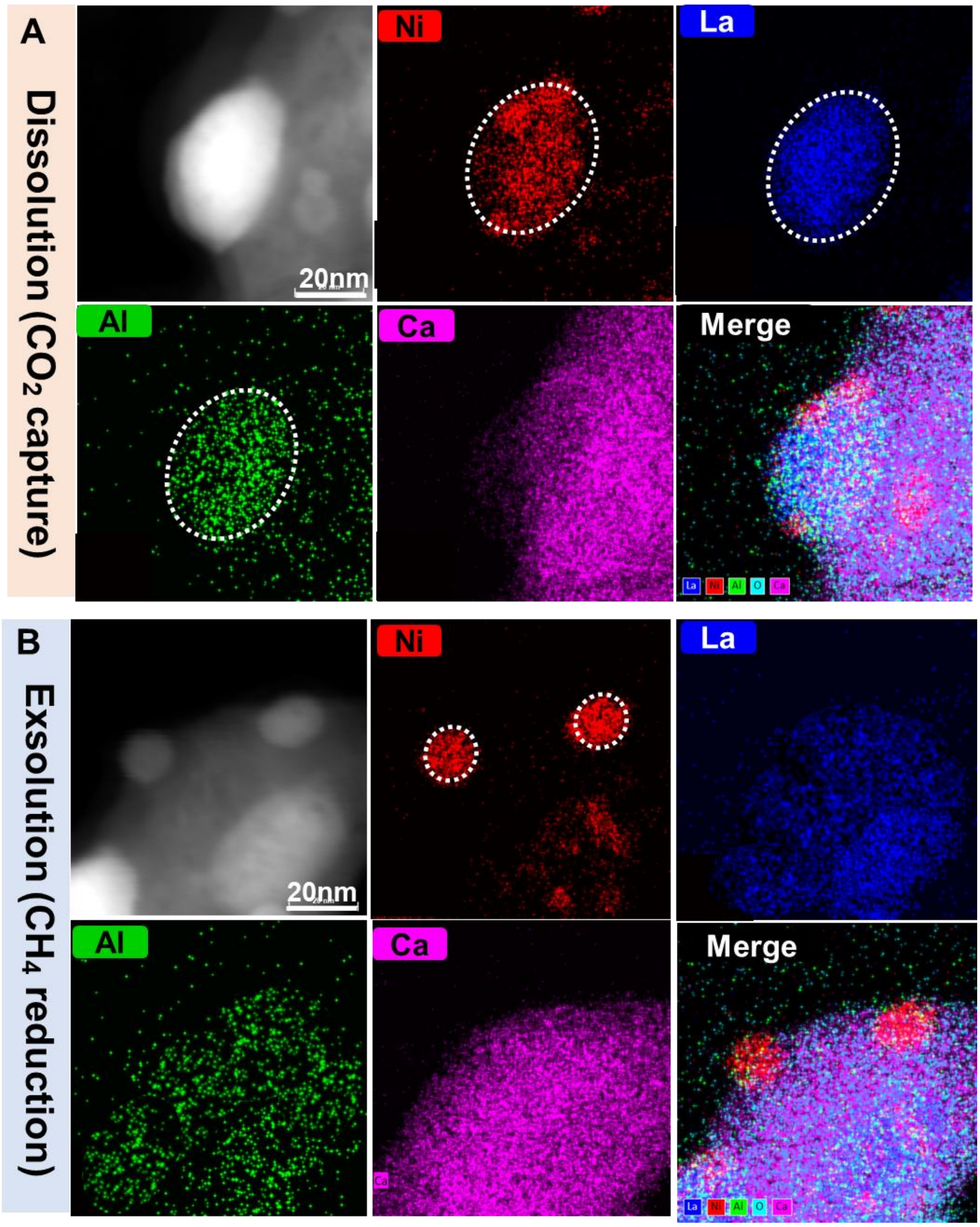

Figure R28(Figure 2D and 2G) STEM-HADDF images of $LaNi_{0.8}Al_{0.2}O_3$/CaO DFM

at the stages of (a) CO_2 adsorption and (b) subsequently DRM conversion by CH_4. Scale bar: 20 nm.

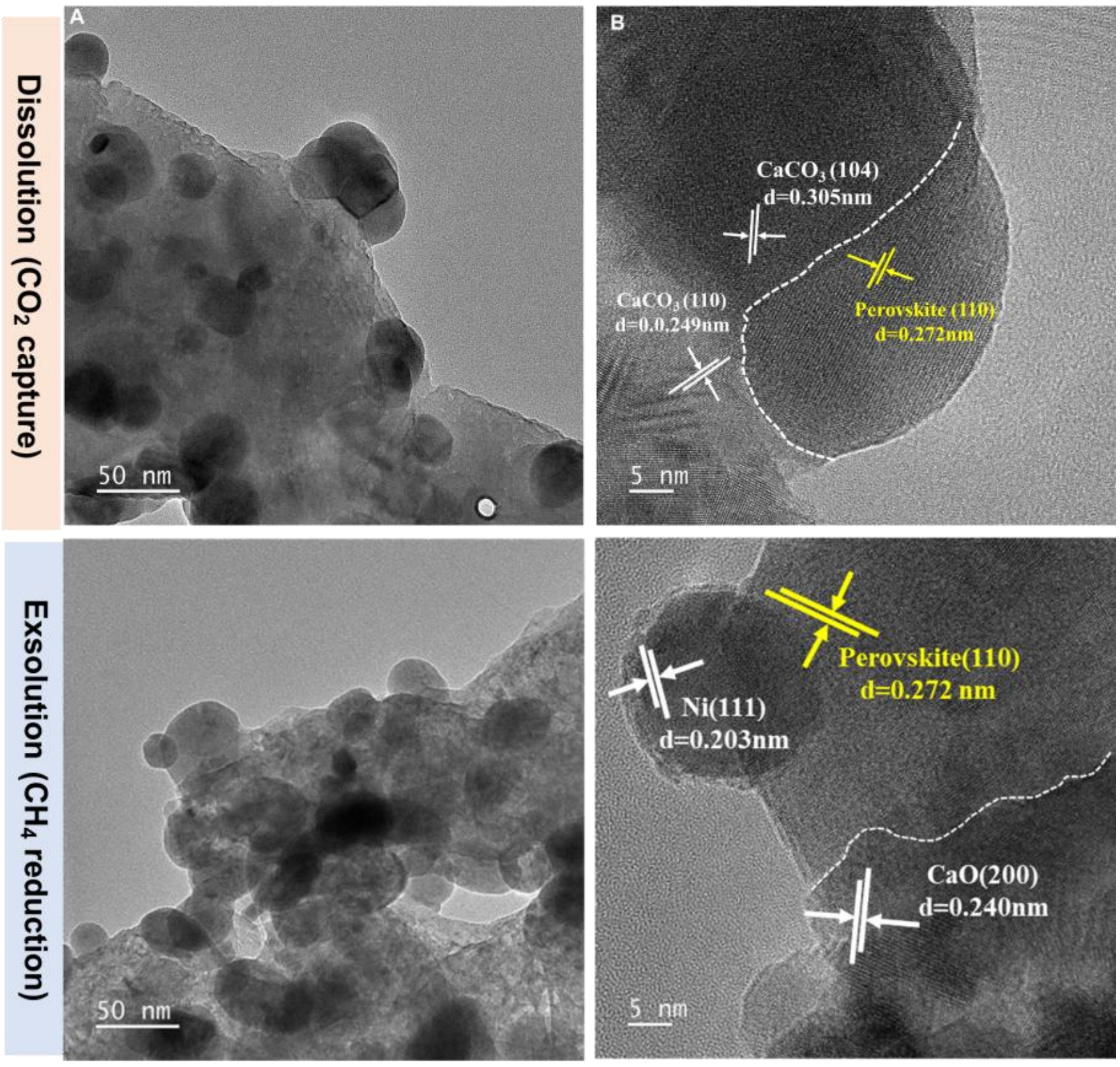

Figure R29 (Figure 2E and 2H) HRTEM images of $LaNi_{0.8}Al_{0.2}O_3$/CaO DFM at the stages of (a) CO_2 adsorption and (b) subsequently DRM conversion by CH_4.

Accordingly, these STEM-EDS images have been added in the Supporting Information, together with the corresponding discussion in the revised manuscript.

(Page 6, Lines 94-100) "…HAADF-STEM image shows that fresh $LaNi_{0.8}Al_{0.2}O_3$ perovskite particles are dispersed in CaO matrix, which will exert a spatial isolation effect to inhibit the sintering of CaO (**Figure S3).** While the EDS mapping images demonstrate the almost overlapped La, Ni, Al elements in $LaNi_{0.8}Al_{0.2}O_3$ perovskites. After the H_2 pretreatment (**Figure S4**), HRTEM images illustrate the well-maintained dispersion state of perovskite particles in $LaNi_{0.8}Al_{0.2}O_3$/CaO DFM (**Figure S5**). From the enlargement, we can see the exsolved Ni NPs (with lattice fringes of 0.203 nm) partially exsolve from the (110) surface (with the lattice of 0.272 nm) of $LaNi_{1-x}Al_xO_3$ perovskite matrix."

(Page 7, Lines 122-128) "Visually, the high-angle annular dark-field signal (HAADF) and energy dispersive spectrometer (EDS) elemental images provide clear evidence that Ni species are evenly distributed with La and Al in $CaCO_3$ matrix (**Figures 2D and S7**),

confirming the exsolved Ni NPs are re-incorporated into the host perovskite under the oxidative CO_2 atmosphere after the capture step. However, some larger Ni NPs still existed in the perovskite due to the kinetic of re-dissolution. Significantly, the $LaNi_{1-x}Al_xO_3$ perovskite particles are in intimate contact with $CaCO_3$ to form heterogeneous interfaces (**Figure 2E**), successfully preventing sintering and agglomeration of Ni NPs and CaO during volumetric expansion.

(Page 8, Lines 137-141) "This was visually evidenced by the HAADF and HRTEM images, that the exsolved Ni NPs with the size of about 15 nm can be explicitly found on the surface of $LaNi_{0.8}Al_{0.2}O_3$/CaO DFM (**Figure S8**), with the distinct lattice fringes of 0.203, 0.272, and 0.240 nm, corresponding to three phases of Ni NPs, $LaNi_{1-x}Al_xO_3$ perovskite and CaO coexisted after the conversion (**Figure 2G** and **2H**)."

Question 4: *"The conclusion that Ni is reversibly exsolving and redistributing because of* $LaAlO_3$ *is not well supported. Since exsolution is usually associated with reducible B-site cations, the rationale for* Al^{3+}*-driven reversible exsolution is not persuasive as currently presented. A more cautious interpretation may be that Ni is remaining more stably dispersed near the surface during cycling, rather than undergoing fully reversible exsolution/redistribution."*

Response: We thank the reviewer for raising this concern. we fully agree with the view proposed by the reviewer that the exsolution of metal particles is related to reducible B-site ions. The core purpose of Al doping in $LaNiO_3$ in this study is to preserve the structural stability of the perovskite lattice for the accommodation of Ni dissolution and exsolution through the metal-support interaction (MSI). It is crucial to emphasize that the core driving force for Ni NPs exsolution and resolution is the feasibility of the valence change of $Ni^0/Ni^{2+}/Ni^{3+}$ at the B-site induced by the alternating reductive-oxidative (redox) atmosphere (**Table S2**). Notably, the substitution of Al^{3+} loosens the bonding constraint of Ni^{3+} species in part and enhances its reducibility. Consequently, the Ni 3p XPS spectra (**Figure 3D**) demonstrates the molar ratio of Ni^{2+}/Ni^{3+} increases with Al doping, accordingly, the in-situ DRM conversion efficiencies of $LaNi_{1-x}Al_xO_3$/CaO (X=0.1, 0.2, 0.3, 0.5) with a similar high CO_2 capacity of about 10.0 mmol g_{DFM}^{-1}, particularly for methane conversion, show a volcano-type approach with the Al-doping level. Because too much higher x of Al-doping results in a significant decrease in exsolution of Ni NPs for CH_4 activation

Table S2 quasi-in situ high-resolution Ni 3p XPS measurements of $LaNi_{0.8}Al_{0.2}O_3$-CaO DFM during iCCC-DRM reaction

**Stage**	Ni^0	Ni^{2+}	Ni^{3+}
**H_2 reduction**	34.50%	36.50%	29.00%
**CO_2 capture**	0	67.40%	32.60%
**CH_4 reduction**	45.85%	17.70%	36.40%

As a control sample, we produced $LaNiO_3$/CaO DFM without Al-doping. As ABO_3 perovskite is a typical intrinsic oxygen-deficient crystalline, all the exsolution-resolution performance and mechanism are similar, but $LaNiO_3$/CaO DFM shows much lower CO_2 adsorption kinetics (**Figure R26a**) and conversion efficiency, compared with durable $LaNi_{0.8}Al_{0.2}O_3$/CaO. The CO_2 capture capacity of $LaNiO_3$/CaO DFM decrease to 6.7 mmol g_{DFM}^{-1} with 33% decline and the CH_4 conversion efficiency decreases to 43.0% from 71.7% after 25 cycles (**Figure R30**).

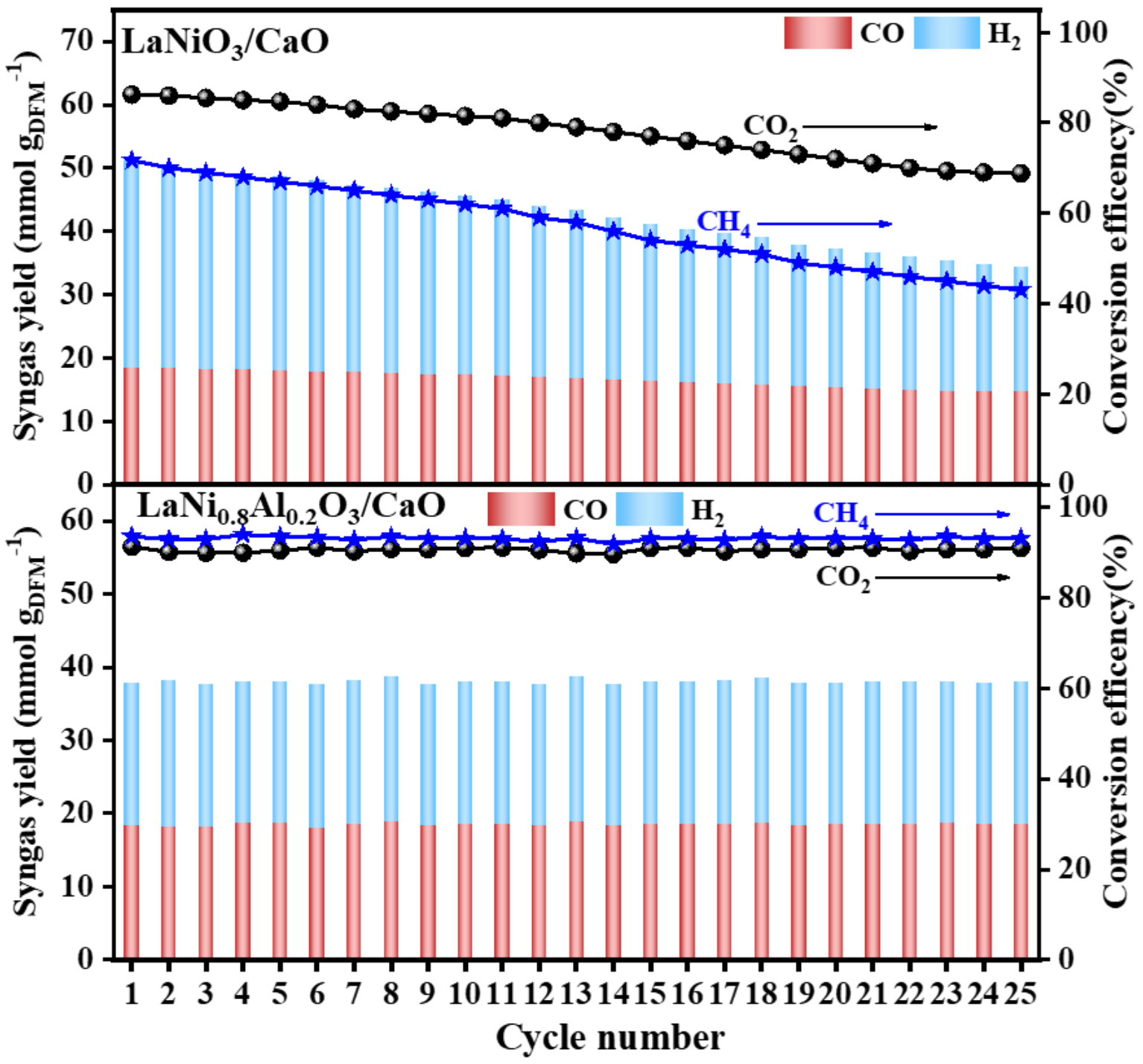

Figure R30 (Figure S1) Comparison of iCCC-DRM performance between $LaNiO_3$/CaO and $LaNi_{0.8}Al_{0.2}O_3$/CaO DFMs.

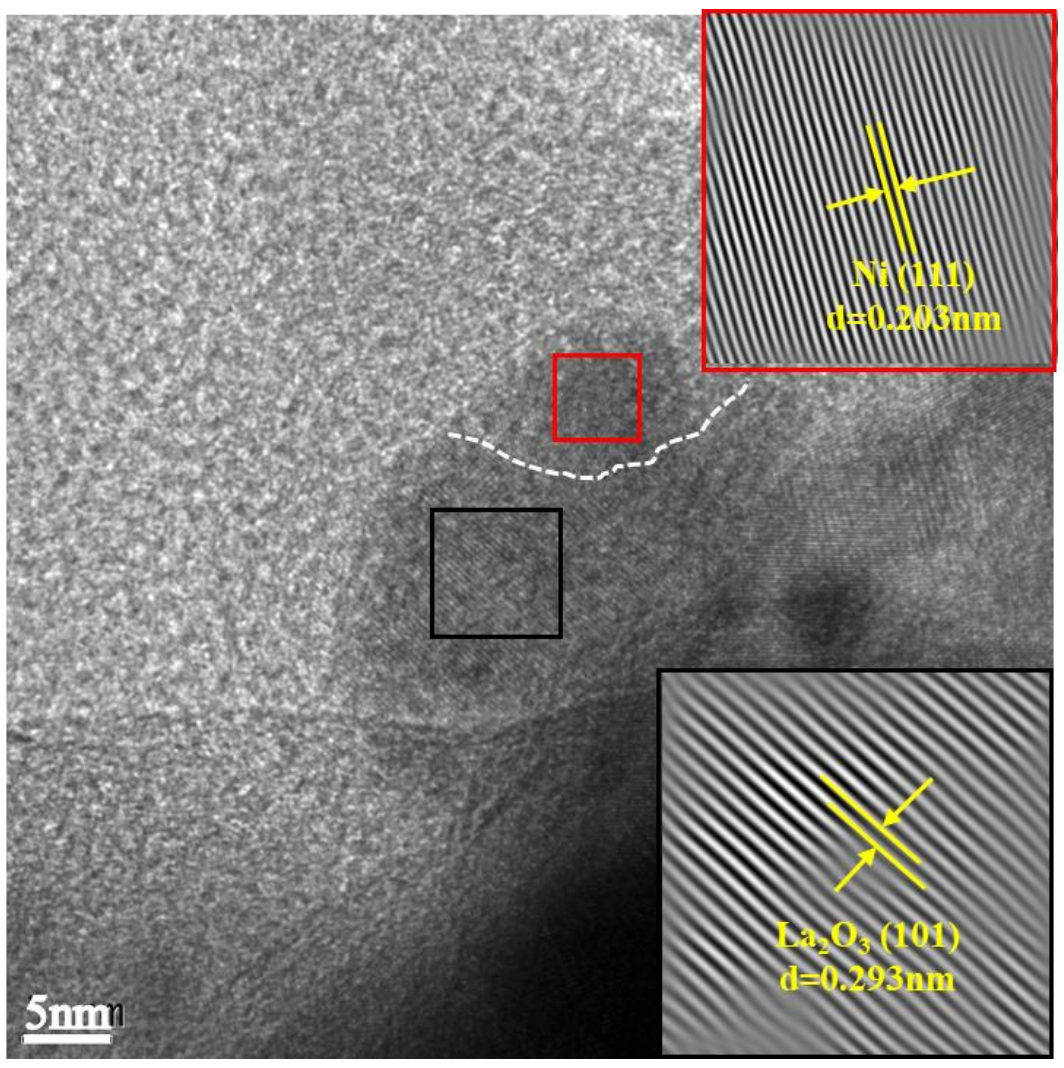

Figure R31(Figure S20) HRTEM images of $LaNiO_3$/CaO DFM after 25th iCCC-DRM cycles.

Specifically, after 25th iCCC-DRM cycles, the HRTEM images of $LaNiO_3$/CaO DFM revels separated Ni and La_2O_3 phases (**Figure R31**). This provides clear evidence that without Al-doping to support the perovskite skeleton, the exsolution of Ni^0 induced by CH_4 reduction will trigger the structural collapse. Even though Ni species can be re-oxidized during CO_2 capture stage, it can hardly reassemble with La_2O_3 species to reconstruct the perovskite crystal structure under iCCC-DRM reaction conditions (**Figure R32**). Therefore, we can see that the conclusion that Al-doping can significantly enhance the reversible exsolution-resolution is persuasive.

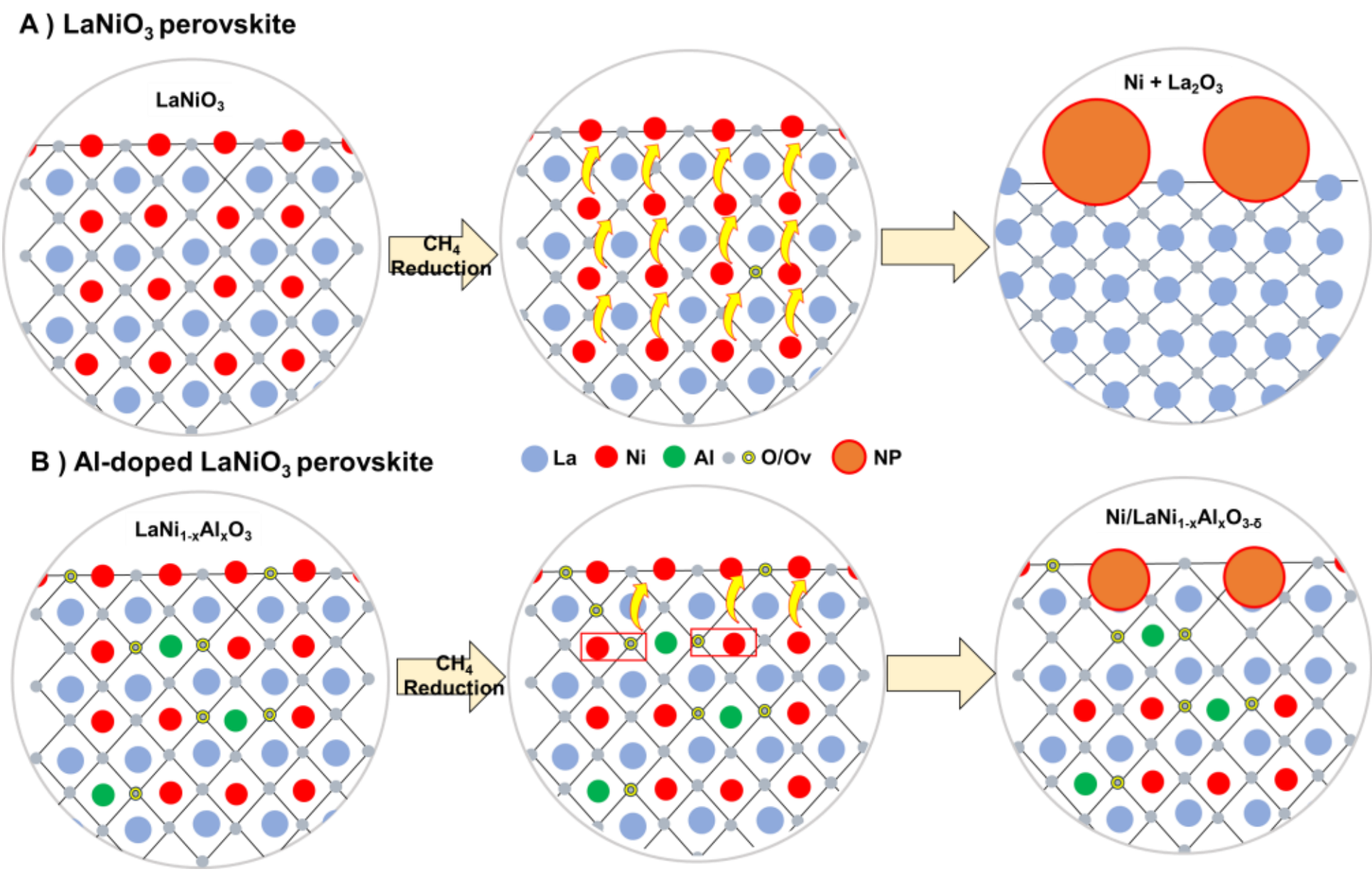

Figure R32 (Figure S27) Schematic illustration of the Ni exsolution process of $LaNiO_3$/CaO and Al-doped $LaNiO_3$/CaO (CaO/$CaCO_3$ transformation is omitted for

clarity)

We fully acknowledge that Ni is remained more stably dispersed near the surface during cycling, rather than undergoing fully reversible exsolution-resolution, since some large-sized Ni NPs cannot be completely reincorporated into the perovskite lattice. Accordingly, we have revised the overstated words that Ni reversible exsolution is driven by Al ions, by that Al doping mainly stabilizes the perovskite lattice to inhibit excessive Ni agglomeration, while Ni exsolution and re-integration are driven by B-site Ni valence change under alternating redox atmosphere. Relevant characterization data and reaction mechanism analysis of Ni valence change during the iCCC-DRM process have been supplemented to enhance the scientific and persuasiveness of the conclusions.

(Page 4, Lines 72-75) "Through heteroatomic Al-doping in the B-site of $LaNiO_3$, the preserved perovskite structure can enhance the MSI for high dispersion of Ni NPs, accommodate the reversible exsolution-resolution under alternating redox atmosphere, produce abundant oxygen vacancies (Ov) via the lattices Jahn-Teller distortion."

(Page 7, Lines 122-128) "Visually, the high-angle annular dark-field signal (HAADF) and energy dispersive spectrometer (EDS) elemental images provide clear evidence that Ni species are evenly distributed with La and Al in $CaCO_3$ matrix (**Figures 2D and S7**), confirming the exsolved Ni NPs are re-incorporated into the host perovskite under the oxidative CO_2 atmosphere after the capture step. However, some larger Ni NPs still existed in the perovskite due to the kinetic retardation during the re-dissolution. Significantly, the $LaNi_{1-x}Al_xO_3$ perovskite particles are in intimate contact with $CaCO_3$ to form heterogeneous interfaces (**Figure 2E**), successfully preventing sintering and agglomeration of Ni NPs and CaO during volumetric expansion.

(Page 8, Lines 137-141) "This was visually evidenced by the HAADF and HRTEM images, that the exsolved Ni NPs with the size of about 15 nm can be explicitly found on the surface of $LaNi_{0.8}Al_{0.2}O_3$/CaO DFM (**Figure S8**), with the distinct lattice fringes of 0.203, 0.272, and 0.240 nm, corresponding to three phases of Ni NPs, $LaNi_{1-x}Al_xO_3$ perovskite and CaO coexisted after the conversion (**Figure 2G** and **2H**)."

(Page 16, Lines 273-278)"Similarly, the LaNiO/CaO without proper Al-doping also exhibited unsatisfactory cyclic stability, and the CO_2 capture capacity of $LaNiO_3$/CaO DFM decreases to 6.7 mmol g^{-1} with 33% decline and the CH_4 conversion efficiency decreases from 71.7% to 43.0% after 25 cycles (**Figures S16- S18**), which can be caused by the Ni NPs sintering due to the collapse of the $LaNiO_3$ structure, and consequently a cascade of CaO sintering (**Figure S20**)."

(Page 24, Lines 373-383) " …Accordingly, the valence changes of Ni^0, Ni^{2+}, and Ni^{3+} at the B-site of $LaNi_{1-x}Al_xO_3$ induced by the alternating reductive-oxidative (redox) atmosphere is the core driving force for Ni NPs exsolution and resolution (**Table S2**). Without Al-doping to support the perovskite skeleton, the exsolution of Ni^0 induced by CH_4 reduction will trigger the structural collapse. Even though Ni species can be re-oxidized during CO_2 capture stage, it can hardly reassemble with La_2O_3 species to reconstruct the perovskite crystal structure under iCCC-DRM reaction conditions (**Figure S27a**). Thus, the core purpose of Al doping in $LaNiO_3$/CaO is to preserve the structural stability of the perovskite lattice for the accommodation of Ni dissolution and exsolution. The well matched CH_4 and adsorbed CO_2 activation owing to the proper MSI between the exsolved Ni NPs and $LaNi_{1-x}Al_xO_3$ perovskite sustains the durable perovskite structure for dynamic Ni NPs exsolution-dissolution during the reversible redox switching of iCCC-DRM, suppressing the notorious factor of catalyst NPs sintering and carbon deposition (**Figure S27b**)"

(Page 24, Lines 397-399) "From a crystallographic perspective, the substitution of Ni^{2+}/Ni^{3+} by smaller Al^{3+} in B-site of perovskite mainly stabilizes the perovskite lattice to inhibit excessive Ni agglomeration, while Ni exsolution and re-integration are driven by B-site Ni valence change under alternating redox atmosphere."

Question 5: *"minor point. Page 6: replace "dopped" with "doped"."*

Response: We appreciate the reviewer for reading our manuscript so carefully. We have carefully checked the language throughout the manuscript and made corrections in the revised manuscript.

References

1. Jo, S. & Gilliard-AbdulAziz, K. L. Self-Regenerative Ni-Doped $CaTiO_3$/CaO for Integrated CO_2 Capture and Dry Reforming of Methane. *Small* e2401156 (2024) .
2. Baamran, K., Lawson, S., Rownaghi, A. A. & Rezaei, F. Reactive Capture and Conversion of CO_2 into Hydrogen over Bifunctional Structured $Ce_{1-x}Co_xNiO_3$/Ca Perovskite-Type Oxide Monoliths. *JACS. Au.* **4**, 101–115 (2023).
3. Wei, H. *et al.* Carbon-resistant bifunctional catalyst composed of $LaFeO_3$ enhanced Ni-CaO for integrated CO_2 capture and conversion. *Carbon Capt. Sci. Technol.* 100358–100358 (2025).
4. Zhang, L., Zhou, Y., Cui, H., Cheng, Z. & Zhou, Z. Performance enhancement of Ni/CaO-Al2O3 dual-function materials for integrated CO_2 capture and conversion via dry reforming of methane. *Chem. Eng. Sci.* **320**, 122635 (2026).
5. Jo, S. B. *et al.* CO_2 green technologies in CO_2 capture and direct utilization processes: methanation, reverse water-gas shift, and dry reforming of methane. *Sustainable Energy Fuels* **4**, 5543–5549 (2020).
6. Hu, J., Hongmanorom, P., Galvita, V. V., Li, Z. & Kawi, S. Bifunctional Ni-Ca based material for integrated CO_2 capture and conversion via calcium-looping dry reforming. *Appl. Catal. B Environ.* **284**, 119734 (2021).
7. Hu, J. *et al.* Tandem distributing Ni into CaO framework for isothermal integration of CO_2 capture and conversion. *Chem. Eng. J.* **452**, 139460 (2023).
8. Han, R. *et al.* Two birds with one stone: MgO promoted Ni-CaO as stable and coke-resistant bifunctional materials for integrated CO2 capture and conversion. *Separation Purif. Technol.* **307**, 122808 (2023).
9. Jiang, R., Miao, Z. & Shen, L. Study on K-modified Ca-based dual-functional materials for carbon capture and in-situ methane dry reforming. *J. Energy Inst.* **117**, 101847 (2024).
10. Wang, F., Han, K., Xu, L., Yu, H. & Shi, W. Ni/SiO_2 Catalyst Prepared by Strong Electrostatic Adsorption for a Low-Temperature Methane Dry Reforming Reaction. *Ind. Eng. Chem. Res.* **60**, 3324–3333 (2021).
11. Hao, C. *et al.* Ce-induced synergistic effect in exsolved perovskite catalyst for highly efficient and robust methane dry reforming. *Nat Commun* **16**, 10630 (2025).
12. Danghyan, V., Kumar, A., Mukasyan, A. & Wolf, E. E. An active and stable NiOMgO solid solution based catalysts prepared by paper assisted combustion synthesis for the dry reforming of methane. *Appl. Catal. B Environ.* **273**, 119056 (2020).
13. Song, Z. *et al.* Improved Effect of Fe on the Stable NiFe/Al_2O_3 Catalyst in Low-Temperature Dry Reforming of Methane. *Ind. Eng. Chem. Res.* **59**, 17250–17258 (2020).
14. Wang, Q. *et al.* Electron-deficient Ni0 sites stabilized by dual Ni/$MgNiO_2$ interface enable efficient and durable dry reforming of methane. *Appl. Catal. B Environ.* **383**, 126105 (2026).

15. Wu, P. *et al.* Cooperation of Ni and CaO at Interface for CO_2 Reforming of CH_4: A Combined Theoretical and Experimental Study. *ACS Catal.* **9**, 10060–10069 (2019).
16. Liu, Y. *et al.* Embedding high loading and uniform Ni nanoparticles into silicalite-1 zeolite for dry reforming of methane. *Appl. Catal. B Environ.* **307**, 121202 (2022).
17. Jin, F. *et al.* Stable Trimetallic NiFeCu Catalysts with High Carbon Resistance for Dry Reforming of Methane. *ChemPlusChem* **85**, 1120–1128 (2020).
18. Daoura, O. *et al.* Mesocellular silica foam-based Ni catalysts for dry reforming of CH_4 (by CO_2). *J. CO_2. Util.* **24**, 112–119 (2018).
19. Cheng, F., Duan, X. & Xie, K. Dry Reforming of CH_4/$CO_2$by Stable Ni Nanocrystals on Porous Single-Crystalline MgO Monoliths at Reduced Temperature. *Angew Chem Int Ed* **60**, 18792–18799 (2021).
20. Wang, Q. *et al.* Interface engineering of oxygen-vacancy-rich MgO/Ni@NiAlO enables low-temperature coke-free methane dry reforming. *Chin. J. Catal.* **75**, 9–20 (2025).
21. Song, Y. *et al.* Dry reforming of methane by stable Ni–Mo nanocatalysts on single-crystalline MgO. *Science* **367**, 777–781 (2020).

Response to Reviewers

Reviewer #1

"*The authors have successfully addressed all of the concerns raised during the previous round of review. In particular, they performed the additional experiments that were suggested and provided direct comparisons with conventional catalyst systems, substantially strengthening the evidence supporting their claims. The responses to the reviewers' questions were accompanied by appropriate references and well-reasoned explanations, which I found convincing.*

With the inclusion of these additional datasets and analyses, I believe that the scientific rigor and completeness of the study have been significantly improved. The manuscript now meets the standard expected for publication in a high-profile Nature Communications journal. Therefore, I recommend acceptance of the manuscript in its current form."

Response: We thank the reviewer for his/her help in making it possible for our work to be published.

Reviewer #2

"*The manuscript was acceptable for publication. The authors addressed all of my concerns.*"

Response: We thank the reviewer for his/her help in making it possible for our work to be published.
